# Role of Stereochemistry on the Biological Activity of Nature-Inspired 3-Br-Acivicin Isomers and Derivatives

**DOI:** 10.3390/molecules28073172

**Published:** 2023-04-03

**Authors:** Andrea Galbiati, Aureliano Zana, Chiara Borsari, Marco Persico, Stefania Bova, Oleh Tkachuk, Alexandra Ioana Corfu, Lucia Tamborini, Nicoletta Basilico, Caterina Fattorusso, Stefano Bruno, Silvia Parapini, Paola Conti

**Affiliations:** 1 Department of Pharmaceutical Sciences, University of Milan, Via Mangiagalli 25, 20133 Milan, Italy; andrea.galbiati@philochem.ch (A.G.); aureliano.zana@philochem.ch (A.Z.); chiara.borsari@unimi.it (C.B.); ioana.corfu@unimi.it (A.I.C.); lucia.tamborini@unimi.it (L.T.); 2 Department of Pharmacy, University of Naples “Federico II”, Via D. Montesano 49, 80131 Naples, Italy; m.persico@unina.it (M.P.); olotuk.6@gmail.com (O.T.); caterina.fattorusso@unina.it (C.F.); 3 Department of Medicine and Surgery, University of Parma, 43124 Parma, Italy; stefania.bova@unipr.it; 4 Department of Biomedical, Surgical and Dental Sciences, University of Milan, Via Pascal 36, 20133 Milan, Italy; nicoletta.basilico@unimi.it; 5 Food and Drug Department, University of Parma, 43124 Parma, Italy; stefano.bruno@unipr.it; 6 Department of Biomedical Sciences for Health, University of Milan, Via Pascal 36, 20133 Milan, Italy; silvia.parapini@unimi.it

**Keywords:** stereochemistry, 3-Br-acivicin, *Plasmodium falciparum*, glyceraldehyde 3-phosphate dehydrogenase, multitarget, covalent inhibitors

## Abstract

Chiral natural compounds are often biosynthesized in an enantiomerically pure fashion, and stereochemistry plays a pivotal role in biological activity. Herein, we investigated the significance of chirality for nature-inspired 3-Br-acivicin (3-BA) and its derivatives. The three unnatural isomers of 3-BA and its ester and amide derivatives were prepared and characterized for their antimalarial activity. Only the (5*S*, α*S*) isomers displayed significant antiplasmodial activity, revealing that their uptake might be mediated by the L-amino acid transport system, which is known to mediate the acivicin membrane’s permeability. In addition, we investigated the inhibitory activity towards *Plasmodium falciparum* glyceraldehyde 3-phosphate dehydrogenase (*Pf*GAPDH) since it is involved in the multitarget mechanism of action of 3-BA. Molecular modeling has shed light on the structural and stereochemical requirements for an efficient interaction with *Pf*GAPDH, leading to covalent irreversible binding and enzyme inactivation. While stereochemistry affects the target binding only for two subclasses (**1a**–**d** and **4a**–**d**), it leads to significant differences in the antimalarial activity for all subclasses, suggesting that a stereoselective uptake might be responsible for the enhanced biological activity of the (5*S*, α*S*) isomers.

## 1. Introduction

Natural products (NPs) have historically played a major role in drug discovery [1] and have been pinpointed as privileged scaffolds for interacting with protein drug targets [2]. The unique chemical diversity and structural complexity of NPs allowed the expansion of the known chemical space explored by medicinal chemists [3]. The majority of NPs are chiral, and they are biosynthesized in an enantiomerically pure fashion [4]. Generally, stereochemistry has a crucial impact on drug action since it affects target binding, metabolism, and distribution. For different compounds classes, stereochemistry is the driver for potency and pharmacokinetics [5]. In addition, it has been shown to affect the protein transport systems, resulting in a stereospecific uptake of drugs, as described for β-lactam antibiotics [6].

**Figure 1:**
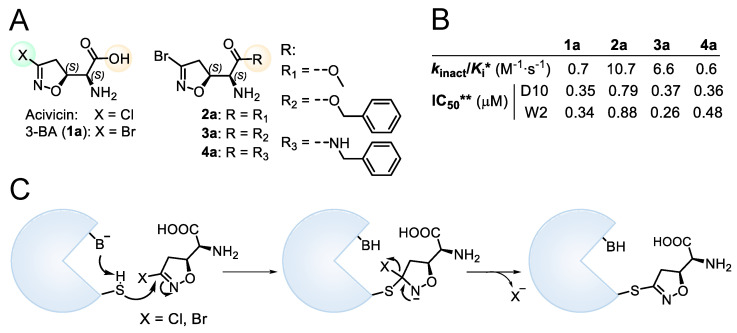
(**A**) Chemical structure of acivicin, 3-BA (**1a**) and 3-BA derivatives (**2a**–**4a**). (**B**) Biological data for compounds **1a**–**4a**. * Second-order rate constant used to characterize covalent binding of irreversible inhibitors to *Pf*GAPDH. ** IC_50_ towards *P. falciparum* D10 and W2 strains. (**C**) Proposed reaction mechanism for the 3-halo-4,5-dihydroisoxazole moiety.

The natural compound (5*S*, α*S*) acivicin (AT-125, Figure 1A), produced by *Streptomyces sviceus*, and its synthetic analogue 3-Br-acivicin (3-BA, **1a**, Figure 1A) have been described as L-glutamine analogues capable of irreversibly inhibiting several glutamine-dependent amidotransferases, including CTP synthetase (CTPS), carbamoyl phosphate synthetase and XMP aminase [7,8,9], and γ-glutamyl transpeptidase [10]. The acivicin/3-BA covalent mechanism of action involves the nucleophilic attack of an activated catalytic cysteine residue of the target enzyme to the Cl/Br-substituted C-3 of the 4,5-dihydroisoxazole ring (Figure 1C) [11,12]. Considering the inhibitory activity towards *Trypanosoma brucei* CTPS, 3-BA was three-fold more potent than acivicin, highlighting that the leaving group plays an important role in the irreversible mode of action. The differences in enzyme inhibition translated into enhanced in vitro and in vivo antitrypanosomal activity for 3-BA [13]. Considering different targets, we had previously reported that acivicin was inactive towards *Plasmodium falciparum* glyceraldehyde 3-phosphate dehydrogenase (*Pf*GAPDH), while 3-BA was able to irreversibly inhibit *Pf*GAPDH*,* a key enzyme of the *Plasmodium* glycolytic pathway [14]. In the erythrocytic stages, the malaria parasite relies heavily on *anaerobic glycolysis* for energy production [15], leading to the identification of glycolytic enzymes as promising targets for the development of antimalarial agents.

We had previously modified the amino acidic portion of **1a** and synthesized a series of analogues, keeping the stereochemistry of the natural product unchanged. The ester (**2a**, **3a**) and amide (**4a**) derivatives of 3-Br-acivicin were investigated for their antimalarial activity on *P. falciparum* cultures and for the ability to covalently inhibit *Pf*GAPDH. All compounds (**1a**–**4a**) showed antiparasitic activity against D10 (chloroquine sensitive) and W2 (chloroquine resistant) strains of *P. falciparum*, with IC_50_ being lower than 1 μM (Figure 1B). The methyl and benzyl esters displayed the highest second-order rate constant (*k*_inact_/*K*_i_) used to characterize the covalent binding of irreversible inhibitors to *Pf*GAPDH (**2a**: 10.7; **3a**: 6.6 M^−1^∙s^−1^; Figure 1B) [14,16]. These outcomes clearly indicated that the similarity with L-Gln was not essential for the inhibition of GAPDH.

Despite the fact that chemical modifications in the amino acidic portion of **1a** have been extensively explored [14,16,17,18], the effect of stereochemistry on the biological recognition processes, protein binding, and antiparasitic activity has not yet been investigated. Herein, we disclosed the synthesis of the unnatural isomers of **1a**–**4a**, and we explored the importance of stereochemistry for the in vitro antimalarial activity and the inhibitory activity towards recombinant *Pf*GAPDH, which is known to be involved in the multi-target mechanism of 3-BA.

## 2. Results and Discussion

### 2.1. Synthesis of Enantiomerically Pure Unnatural Isomers

**Scheme 1:**
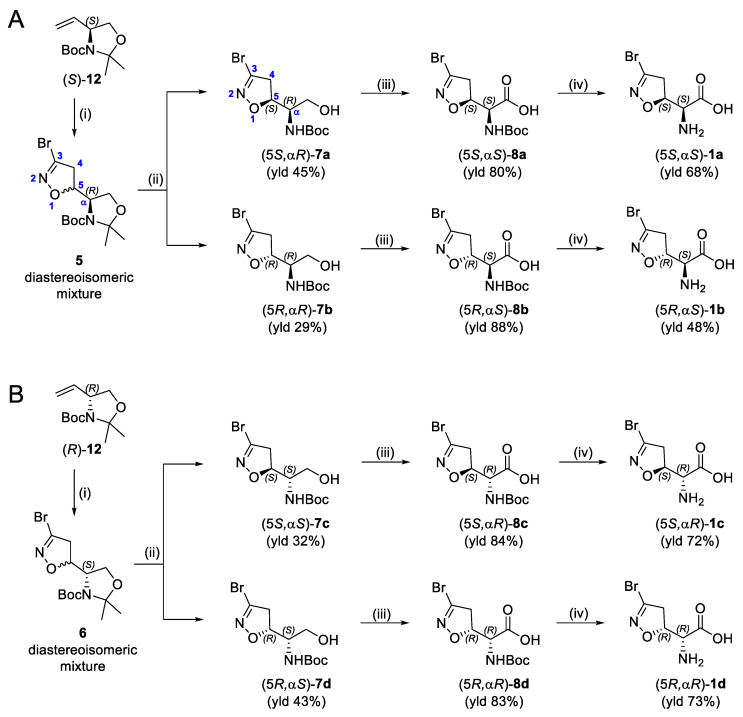
(**A**,**B**) Synthesis of compounds **1a**–**1b** (**A**) and **1c**–**1d** (**B**). Reagents and conditions: (i) according to Ref. [19]; (ii) AcOH/H_2_O 5:1, 40 °C, 48 h; (iii) TEMPO, Fe(NO_3_)_3_·9H_2_O, KCl, air flux, DCE, r.t., 24 h; (iv) (1) 15% TFA, DCM, r.t., 1 h; (2) column purification with Dowex Marathon C (H^+^ form) resin, 10%Py/H_2_O (eluent).

The synthesis of the enantiomerically pure target compounds started by using the chiral synthons (*S*)-**12** or (*R*)-**12**. The diastereoisomeric mixtures **5** and **6** were obtained through the 1,3-dipolar cycloaddition of bromonitrile oxide to alkenes (*S*)-**12** and (*R*)-**12**, respectively, as previously described [19]. Treatment of **5** with a 5:1 mixture of AcOH/H_2_O provided alcohols **7a** and **7b** (Scheme 1A) that were separated by flash chromatography. Analog conditions led to the synthesis of alcohols **7c** and **7d**, starting from **6** (Scheme 1B). Alcohols **7a**–**d** were oxidized to the corresponding carboxylic acids **8a**–**d** using Fe(NO_3_)_3_·9H_2_O, TEMPO, and KCl under an air flux as an O_2_ source [20]. This is a cleaner method compared to the traditional oxidation procedures used to convert alcohols into carboxylic acids, which typically involve the use of chromium-based reagents, and it allowed us to obtain the desired products in high yields (80–88%). The final amino acids **1a**–**d** were obtained after treatment of the *N*-Boc precursors (**8a**–**d**) with a 15% TFA solution in dichloromethane. The zwitterionic compounds were isolated after ion-exchange chromatography employing the DOWEX Marathon C (H^+^ form) resin.

**Scheme 2:**
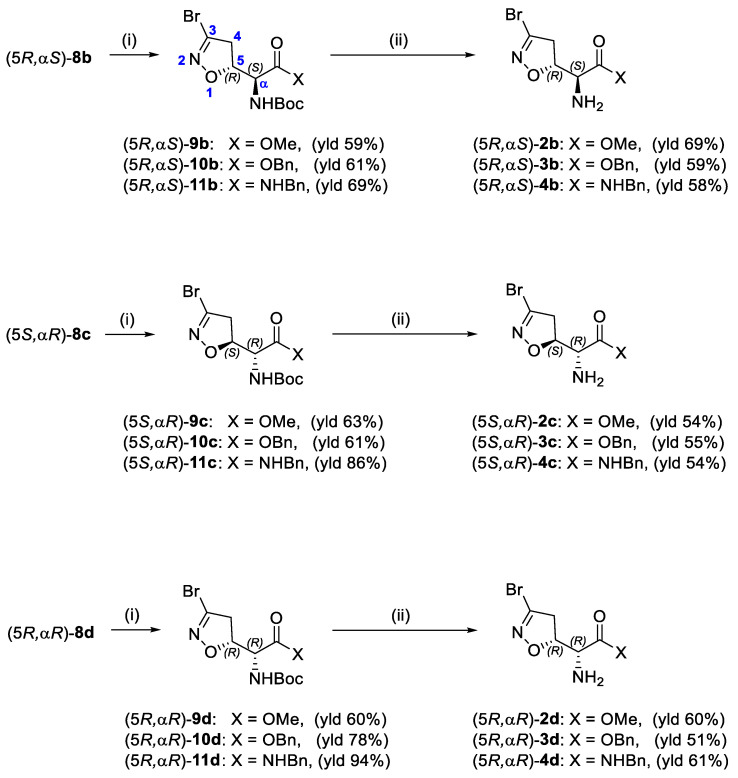
Synthesis of compounds **2b**–**d**, **3b**–**d**, and **4b**–**d**. Reagents and conditions: (i) 2N TMSCHN_2_ in hexane, PhMe/MeOH, r.t., 1 h (for **9**); BnBr, KHCO_3_, DMF, 50 °C, 1 h (for **10**); BnNH_2_, EDC·HCl, HOBt, dry THF, r.t., 2 h (for **11**); (ii) 15% TFA/DCM, r.t., 2–4 h.

Compounds **2b**–**d**, **3b**–**d**, and **4b**–**d** were synthesized following the procedure previously described for the (5*S*, α*S*) isomers **2a**, **3a**, and **4a** [16]. Briefly, the *N*-Boc-protected carboxylic acids **8b**–**d** were treated with trimethylsilyldiazomethane and methanol at room temperature to obtain the methyl esters **9b**–**d** (Scheme 2). The reaction of **8b**–**d** with benzyl bromide under basic conditions at 50 °C afforded the benzyl esters **10b**–**d**. The coupling reaction between the carboxylic acids **8b**–**d** and benzylamine using EDC hydrochloride and HOBt provided the amides **11b**–**d**. Intermediates **9b**–**d**, **10b**–**d**, and **11b**–**d** were treated with a 15% TFA solution in dichloromethane to cleave the Boc-protecting group and provide the free amines **2b**–**d**, **3b**–**d**, and **4b**–**d**, respectively (Scheme 2).

### 2.2. Antimalarial Activity against Plasmodium falciparum

All the diastereoisomers were investigated for their in vitro antiplasmodial activity. Phenotypic assays were performed against D10 (chloroquine sensitive) and W2 (chloroquine resistant) *P. falciparum* strains, using the parasite lactate dehydrogenase (pLDH) method and chloroquine (CQ) as the control. Considering all four compound subclasses (**1a**–**d**, **2a**–**d**, **3a**–**d**, **4a**–**d**), the compounds showing the natural configuration (5*S*, α*S*) were significantly more active than the corresponding enantiomers and diastereoisomers. **1a**–**4a** showed sub-micromolar IC_50_s against both *P. falciparum* strains (IC_50_ < 1 μM), with no significant differences between the two strains (D10 and W2, Table 1). While the natural (5*S*, α*S*) isomers (**1a**–**4a**) were potent antimalarial agents, the isomers possessing the (5*R*, α*R*) absolute configuration displayed a moderate antiplasmodial activity (1 < IC_50_ < 10 μM). The (5*S*, α*R*) and (5*R*, α*S*) absolute configurations resulted in a huge drop in the antimalarial potency, leading to poorly active compounds (Table 1).

For the methyl ester derivatives, the enantiomer of **2a** [(5*R*, α*R*)-**2d**] was approximately 10-fold less potent than **2a** towards *P. falciparum* D10 and W2 strains, whereas the diastereoisomers (**2b** and **2c**) were inactive towards both strains (IC_50_ > 15 μM, Table 1). The same trend was observed for the other subclasses (**1a**–**d**, **3a**–**d**, and **4a**–**d**), with the exception of **1c**, which presented an IC_50_ < 10 μM (Table 1). Thus, the stereochemistry led to significant differences in the antimalarial activity, with the natural isomers always being the most potent molecules. Since all the isomers in each subclass (**1a**–**d**, **2a**–**d**, **3a**–**d**, and **4a**–**d**) have the same clogD, the differences in antiplasmodial potency could not be correlated to a different passive diffusion across the cell membranes. The natural compound (5*S*, α*S*) acivicin is known to compete with amino acids for cellular uptake via transporters. Indeed, acivicin uptake is mediated by the L-amino acid transport system that is responsible for the Na^+^-independent transport of neutral amino acids [21,22]. The microenvironment of *P. falciparum*-infected human erythrocytes is controlled by two membranes. Normal mature erythrocytes are relatively impermeable to L-Gln, and therefore also acivicin. The influx of L-Gln and other amino acids is controlled by different transporter systems, each varying their substrate specificity [23,24]. Our results suggested that the stereochemistry might be relevant for recognition by the putative transporters, not only for the amino acid 3-BA (**1a**), but also for its ester and amide derivatives (**2a**–**4a**). Indeed, only the (5*S*, α*S*) natural isomers (**1a**–**4a**) showed a significant potency in inhibiting *P. falciparum* proliferation. To investigate whether the different antiplasmodial activities could be related also to a distinct interaction of the isomers with biological targets, we tested the inhibitory activity towards *Pf*GAPDH, which is a known target for **1a**–**4a** [16].

### 2.3. In Vitro Inhibitory Activity towards PfGAPDH

**Figure 2:**
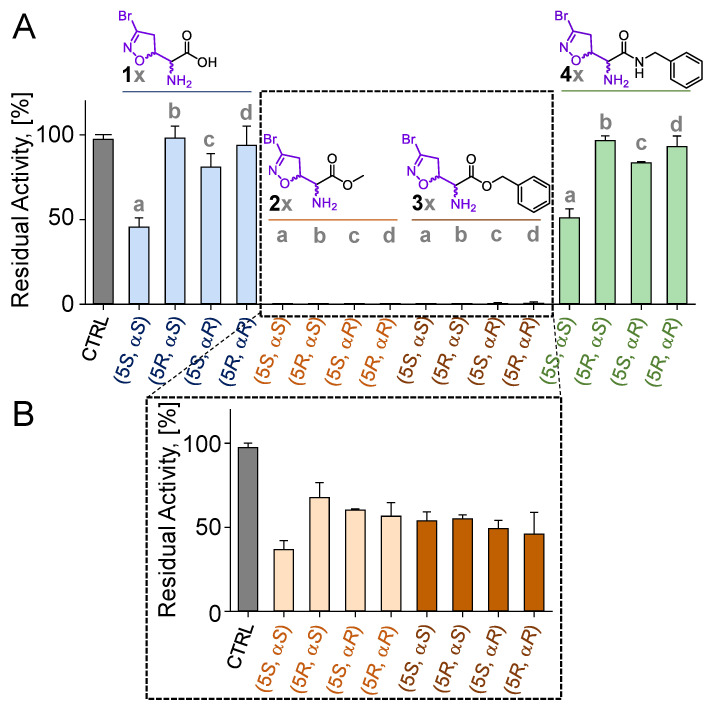
(**A**,**B**) Residual activity (%) of *Pf*GAPDH upon incubation with 100 µM of compounds **1a**–**d**, **2a**–**d**, **3a**–**d**, and **4a**–**d** for 3 h (**A**) or 50 µM of compounds **2a**–**d** and **3a**–**d** for 30 min (**B**). All experiments were performed at 25 °C in a buffered solution containing 10 mM of TEA, 5 mM of EDTA, and 10 mM of sodium arsenate, pH 7.6. *Pf*GAPDH was at 2 µM concentration. CTRL: activity of *Pf*GAPDH maintained under the same conditions in the absence of inhibitors. Experiments were performed in independent triplicates. Data are shown as mean ± SD.

3-Br-acivicin (**1a**) biological activity is correlated to its ability to inhibit multiple targets. Among these, *Pf*GAPDH had been previously suggested to be involved in the antimalarial effect of **1a** and other structurally related compounds [16]. For this reason, the four diastereoisomers of each compound were investigated for their ability to inhibit the *Pf*GAPDH activity. All molecules were screened at a concentration of 100 μM after a 3 h incubation time. While 3-Br-acivicin (**1a**) produced a *Pf*GAPDH inhibition higher than 50%, its enantiomer **1d** and diastereoisomers (**1b** and **1c**) were inactive (Figure 2A). A similar trend was observed for the amide-bearing isomers **4a** compared to **4b**–**d**. In contrast, all four isomers of the methyl (**2a**–**d**) and benzyl (**3a**–**d**) ester derivatives were able to fully inhibit *Pf*GAPDH at a 100 μM concentration, leading to a residual *Pf*GAPDH activity lower than 1% (Figure 2A). Therefore, compounds **2a**–**d** and **3a**–**d** were selected for a further investigation and tested at 50 µM for 30 min. All diastereoisomers (**2a**–**d** and **3a**–**d**) showed enzyme inhibition >50%, with the natural isomer **2a** being the most active (37% residual activity at 50 µM; Figure 2A).

The covalent mechanism of action of **2a** and **3a** had been already characterized though mass spectrometry, using undigested and trypsin-digested *Pf*GAPDH after incubation with the tested compounds [14]. We also proved the selective modification of the catalytic Cys153 with no involvement of other Cys residues [14]. In addition, the *k*_inact_/*K*_i_ ratio typically used to characterize the covalent binding of irreversible inhibitors to the target protein was measured for compounds **1a**–**4a** [16]. Therefore, we can assume that the unnatural isomers **2b**–**4b**, **2c**–**4c**, and **2d**–**4d** displayed the same irreversible mechanism of inhibition observed for their corresponding natural isomers (5*S*, α*S*) **1a**–**4a**.

### 2.4. Molecular Modeling

**Figure 3:**
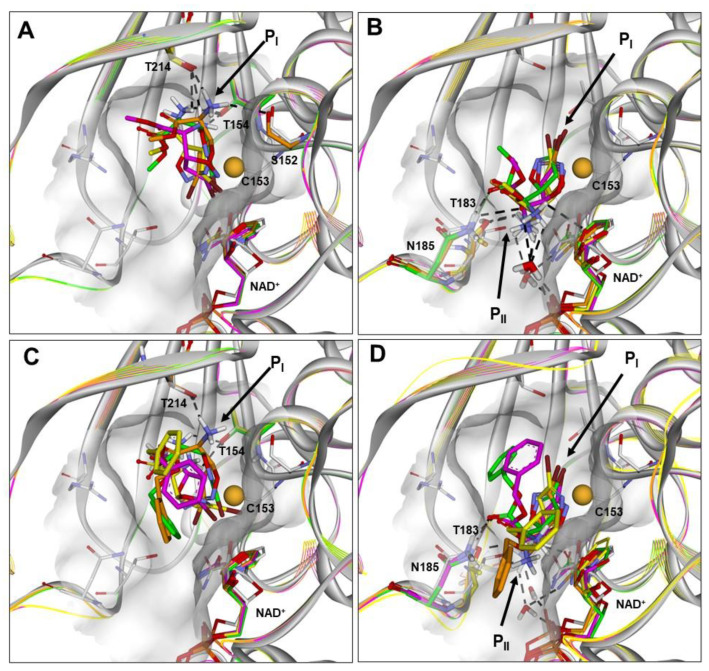
(**A**–**D**) Docked structures of *Pf*GAPDH in complex with: (i) **2a** (orange), **2b** (yellow), **2c** (green), and **2d** (magenta) approaching from the P_I_ site (BA1; **A**) or P_II_ site (BA2; **B**); (ii) **3a** (orange), **3b** (yellow), **3c** (green), and **3d** (magenta) approaching from the P_I_ site (BA1; **C**) or P_II_ site (BA2; **D**). All structures are superimposed on the starting *Pf*GAPDH conformation by fitting Cα atoms. The starting protein conformation (gray) is displayed as solid ribbons and the Connolly surface of the active site is shown. The backbone of the docked complexes is in line ribbons, key interaction residues are displayed and labeled, and heteroatoms are colored by atom type (N, blue; O, red; S, yellow; Br, brown). The van der Waals volume of the sulfur atom of C153 is scaled by 50%. Hydrogen atoms are omitted for clarity, except those involved in ligand–protein hydrogen bond interactions (black dashed lines).

**Figure 4:**
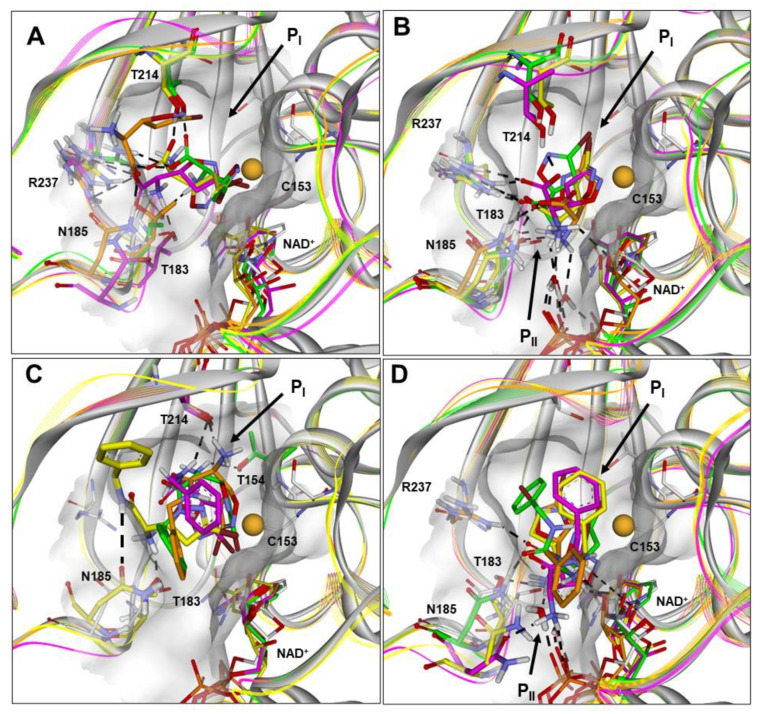
(**A**–**D**) Docked structures of *Pf*GAPDH in complex with: (i) **1a** (orange), **1b** (yellow), **1c** (green), and **1d** (magenta) approaching from the P_I_ site (BA1; **A**) or P_II_ site (BA2; **B**); (ii) **4a** (orange), **4b** (yellow), **4c** (green), and **4d** (magenta) approaching from the P_I_ site (BA1; **C**) or P_II_ site (BA2; **D**). All structures are superimposed on the starting *Pf*GAPDH conformation by fitting Cα atoms. The starting protein conformation (gray) is displayed as solid ribbons and the Connolly surface of the active site is shown. The backbone of the docked complexes is in line ribbons, key interaction residues are displayed and labeled, and heteroatoms are colored by atom type (N, blue; O, red; S, yellow; Br, brown). The van der Waals volume of the sulfur atom of C153 is scaled by 50%. Hydrogen atoms are omitted for clarity, except those involved in ligand–protein hydrogen bond interactions (black dashed lines).

Compounds **1a**–**1d**, **2b**–**2d**, **3b**–**3d**, and **4b**–**4d** were subjected to conformational analysis and flexible docking simulations in a complex with *Pf*GAPDH using the same computational procedure previously applied to **2a**, **3a**, and **4a** [16]. The present results (Tables S1–S38) were integrated with those previously obtained, analyzed, and related to the inhibitory activities of the compounds. The aim was to simulate the binding of the compounds to *Pf*GAPDH just before undergoing nucleophilic attack by the catalytic cysteine residue (C153). According to the flip–flop model proposed for the catalytic reaction mechanism of GAPDH, the substrate rotates around C153, moving the C-3 phosphate from a first (P_I_) to a second (P_II_) interaction site [25,26]. In line with these findings, different starting complexes were considered and two possible binding approaches to C153, named BA1 and BA2, were obtained for each compound, which corresponded to the approach from the P_I_ or P_II_ site, respectively (Figure 3 and Figure 4; Tables S15–S32).

The geometry and the conformational energy of the docked ligands were compared to those of the conformers obtained by the conformational analysis (Table S33). The solvent-accessible surface area (SASA) of the leaving group (bromine atom) was calculated (Table S34), and the ligand–protein interactions were analyzed (Tables S35–S38).

It resulted that the active isomers **2a**–**2d** could approach C153 assuming both binding modes. In BA1 (Figure 3A and Figure S1), the protonated amine function and the ester function interacts with the P_I_ site, while the bromine atom points towards NAD^+^.

In BA2 (Figure 3B and Figure S2), the protonated amine and the ester function are located at the P_II_ site, and the bromine atom is positioned at the P_I_ site pointing towards the active-site segment. Considering the orientation of the leaving group, this binding approach is favored by a larger solvent-accessible surface area (SASA) of the bromine atom (Table S34). However, assuming BA1 (Figure 3A and Figure S1), the bromine atom could leave the active site together with NAD_+_, similarly to the hydride ion of the substrate. The putative binding conformations of **2a**–**2d** resulted all within 5 kcal/mol from the global energy minimum (GM) (Table S33).

Similar results were obtained for the active diastereoisomers **3a**–**3d**, although they were characterized by a more hindered benzyl moiety compared to the methyl group of **2a**–**2d** (Figure 3C,D, Figures S3 and S4). In particular, assuming BA1, the phenyl ring is positioned in the large cleft between the S-loop (aa182–210) and the S7–S8 loop (aa122–130), being slightly differently oriented according to the stereochemistry of the C5 carbon (occupying the P_II_ site in the 5*S* diastereoisomers **3a** and **3c**; Figure 3C). On the other hand, in the BA2, the phenyl ring is positioned according to the stereochemistry of the Cα carbon being oriented toward the P_I_ site or the P_II_ site in the α*R* (**3c**, **3d**) and α*S* (**3a**, **3b**) diastereoisomers, respectively (Figure 3D).

On the other hand, the results obtained for **1a**–**1d** suggest that a salt–bridge interaction between the negatively charged carboxylic group of the ligands and R237 of *Pf*GAPDH (involved in phosphate binding and translocation) could be responsible for the drastic reduction in the inhibitory activity of **1b**–**1d**. Indeed, when the carboxylic group is positioned at the P_I_ site (BA1; Figure 4A and Figure S5), the salt–bridge with R237 is responsible for ensuring the electrophilic carbon of the 3-bromo-4,5-dihydroisoxazole ring (C3) stays away from the sulfur atom of C153 (>4 Å; **1b** and **1c**) or, in the case of **1d**, placing the 4,5-dihydroisoxazole nitrogen atom between the C3 carbon and the C153 sulfur atom. In any case, such ionic interaction hampers the approach to C153 and the formation of the carbon sulfur bond (~1.80 Å).

When the carboxylic group is positioned in the P_II_ site (BA2; Figure 4B and Figure S6), the diastereoisomers **1b**–**1d** still present the salt–bridge with R237 together with a charge-assisted hydrogen bond interaction with T214, drastically reducing the SASA of the bromine atom positioned at P_I_ (Table S34). Importantly, the carboxylic group of **1a** (the only active diastereoisomer) is not involved in any interaction with R237, and, accordingly, the SASA of the bromine atom is not reduced (Tables S34 and S35). In line with what was observed for the carboxylic group, for the amide derivatives **4b** and **4d**, the introduction of the NH group is responsible for additional interactions with the protein, which move the ligand away from C153 (Figure 4C,D; Tables S27–S30; Figures S7 and S8). As previously reported, this is also true for **4a** (the only active diastereoisomer), but only assuming the BA2 [16]. Finally, diastereoisomer **4c** docked conformations are highly distorted in both binding approaches (Figures S9 and S10) and show an energy difference from the global minimum conformer higher than 15 kcal/mol (Table S33). Therefore, the computational results are consistent with *Pf*GAPDH inhibitory activities and provide useful molecular models to drive future structural modifications.

## 3. Materials and Methods

### 3.1. Chemistry

#### 3.1.1. General

All reagents, solvents, and starting materials were purchased from Sigma-Aldrich (Italy), Fluorochem, or TCI Europe. The diastereoisomeric mixtures **5** and **6** were prepared as previously described [19]. Compounds **1a**, **2a**, **3a**, and **4a** were prepared from intermediate **8a** as previously described [13,14,16]. ^1^H NMR and ^13^C NMR spectra were recorded with a Varian Mercury 300 (300 MHz) spectrometer. NMR spectra were obtained in deuterated solvents, such as CDCl_3_ or D_2_O. The chemical shift (δ values) is reported in ppm and corrected to the signal of the deuterated solvents. Peak multiplicities are reported as: s (singlet), d (doublet), dd (doublet of doublets), ddd (doublet of doublet of doublets), t (triplet), dt (doublet of triplets), q (quartet), m (multiplet), and br (broad). Chemical shifts (δ) are expressed in ppm and coupling constants (*J*) in Hertz (Hz). Thin layer chromatography (TLC) plates were purchased from Sigma-Aldrich Italy (silica gel 60 F254 aluminum sheets, with fluorescence indicator 254 nm); a dilute alkaline solution of KMnO_4_ or a ninhydrin solution were used to visualize the compounds. Flash chromatography was performed using silica gel, with a pore size of 60 Å, and a mesh particle size of 230–400. Specific rotations ([α]_D_^T^) were calculated based on the optical rotation measurements obtained with a Jasco P1010 polarimeter coupled with a Haake N3-B thermostat.

#### 3.1.2. General Procedure A

To a stirred solution of the appropriate *tert*-butyl (1-(3-bromo-4,5-dihydroisoxazol-5-yl)-2-hydroxyethyl)carbamate precursor **7a**–**d** (1 eq) in DCE (0.8 mL/mmol), KCl (0.1 eq), Fe(NO_3_)_3_ × 9H_2_O (0.1 eq) and TEMPO (0.1 eq) were added. The reaction mixture was stirred under an air flow for 24 h, and the reaction progress was monitored by TLC. The solvent was evaporated, and purification using silica gel column chromatography afforded the compounds **8a**–**d**.

#### 3.1.3. General Procedure B

Compounds **8b**–**d** (1.0 eq) were treated with a 15% DCM solution of trifluoroacetic acid (10 eq) at 0 °C, and the solution was stirred at rt for 1 h. The volatiles were removed under vacuum and the crude was purified by ion-exchange chromatography using Dowex Marathon C (H^+^ form) resin, as well as elution with a 10% solution of pyridine in water. The solvent was evaporated under a reduced pressure to obtain the compounds **1b**–**d**.

#### 3.1.4. General Procedure C

TMSCHN_2_ 2.0 M in hexane (2 eq) was added dropwise to a cooled solution of **8b**–**d** (1 eq) in toluene (10 mL/mmol) and MeOH (0.4 mL/mmol). After stirring at room temperature for 1 h, the mixture was concentrated under reduced pressure, and purification using silica gel column chromatography afforded the compounds **9b**–**d**.

#### 3.1.5. General Procedure D

Compounds **8b**–**d** (1 eq) were dissolved in DMF (6 mL/mmol). Benzyl bromide (1.2 eq) and KHCO_3_ (1.2 eq) were added to the solution. The reaction mixture was stirred at 50 °C for 1 h. The reaction mixture was diluted with EtOAc and the organic layer was washed with 1N HCl, 5% NaHCO_3_, and brine. The organic layer was dried over anhydrous Na_2_SO_4_, filtered, and evaporated to reach dryness. Purification using silica gel column chromatography afforded the compounds **10b**–**d**.

#### 3.1.6. General Procedure E

Benzylamine (1 eq), EDC hydrochloride (1 eq), and HOBt (0.5 eq) were added to a solution of **8b**–**d** (1 eq) in dry THF (35 mL/mmol). The reaction mixture was stirred at room temperature for 2 h, then the solvent was removed under reduced pressure. Purification using silica gel column chromatography afforded the compounds **11b**–**d**.

#### 3.1.7. General Procedure F

The *N*-Boc precursor **9b**–**d**, **10b**–**d**, or **11b**–**d** (1 eq) was treated with a 15% DCM solution of trifluoroacetic acid (10 eq) at 0 °C. The resulting solution was stirred at rt for 4 h. The volatiles were removed under vacuum, 5% aqueous solution of NaHCO_3_ was added, and the aqueous layer was extracted with DCM. The organic layer was dried over anhydrous Na_2_SO_4_, filtered, and evaporated to dryness. Purification using silica gel column chromatography afforded the compounds **2b**–**d**, **3b**–**d**, or **4b**–**d**.

#### 3.1.8. *tert*-Butyl ((*R*)-1-((*S*)-3-bromo-4,5-dihydroisoxazol-5-yl)-2-hydroxyethyl)carbamate (**7a**) and *tert*-butyl ((*R*)-1-((*R*)-3-bromo-4,5-dihydroisoxazol-5-yl)-2-hydroxyethyl)carbamate (**7b**)

The diastereomeric mixture **5** (2.80 g, 8.02 mmol) was dissolved in a 5:1 mixture of AcOH/H_2_O (60 mL) and stirred at 40 °C for 48 h. The solvent evaporated, and the crude was dissolved in EtOAc and washed with water. Purification using silica gel column chromatography (cyclohexane/EtOAc 9:1) afforded I fraction **7a** (1.11 g, 3.61 mmol, 45% yield) and II fraction **7b** (719 mg, 2.33 mmol, 29% yield).

**7a**: white prisms (from *i*Pr_2_O), [α]_D_^20^ = +91.8 (c = 0.5, CHCl_3_). ^1^H NMR (300 MHz, CDCl_3_) δ 5.11 (br, 1H), 4.77 (dt, *J* = 8.7, 8.7 Hz, 1H), 3.99–3.88 (m, 1H), 3.82–3.67 (m, 2H), 3.29 (d, *J* = 8.7 Hz, 2H), 2.10 (br, 1H), and 1.45 (s, 9H). ^13^C NMR (75 MHz, CDCl_3_) δ 156.2, 138.5, 81.0, 80.5, 61.4, 54.2, 44.6, and 28.5 (3C). The analytical data were in agreement with the values obtained in the literature [13].

**7b**: white prisms (from *i*Pr_2_O), [α]_D_^20^ = −91.5 (c = 0.5, CHCl_3_). ^1^H NMR (300 MHz, CDCl_3_) δ 5.01–4.88 (m, 2H), 3.90–3.65 (m, 3H), 3.32 (dd, *J* = 17.5, 10.5 Hz, 1H), 3.21 (dd, *J* = 17.5, 8.4 Hz, 1H), 2.05 (br, 1H), 1.45 (s, 9H). ^13^C NMR (75 MHz, CDCl_3_) δ 156.5, 138.4, 80.9, 80.2, 62.3, 54.1, 43.9, 28.2 (3C).

#### 3.1.9. *tert*-Butyl ((*S*)-1-((*S*)-3-bromo-4,5-dihydroisoxazol-5-yl)-2-hydroxyethyl)carbamate (**7c**) and *tert*-butyl ((*S*)-1-((*R*)-3-bromo-4,5-dihydroisoxazol-5-yl)-2-hydroxyethyl)carbamate (**7d**)

The diastereomeric mixture **6** (2.80 g, 8.02 mmol) was dissolved in a 5:1 mixture of AcOH/H_2_O (60 mL) and stirred at 40 °C for 48 h. The solvent evaporated, and the crude was dissolved in EtOAc and washed with water. Purification using silica gel column chromatography (cyclohexane/EtOAc 9:1) afforded I fraction **7d** (1.07 g, 3.45 mmol, 43% yield) and II fraction **7c** (793 mg, 2.57 mmol, 32% yield).

**7c**: white prisms (from *i*Pr_2_O), [α]_D_^20^ = +90.1 (c = 0.5, CHCl_3_). The NMR data were consistent with those obtained for the corresponding enantiomer **7b**.

**7d**: white prisms (from *i*Pr_2_O), [α]_D_^20^ = −90.3 (c = 0.5, CHCl_3_). The NMR data were consistent with those obtained for the corresponding enantiomer **7a**.

#### 3.1.10. (*S*)-2-((*S*)-3-Bromo-4,5-dihydroisoxazol-5-yl)-2-((*tert*-butoxycarbonyl)amino)acetic acid (**8a**)

Compound **8a** was prepared according to general procedure A from intermediate **7a** (1.00 g, 3.23 mmol). Purification using silica gel column chromatography (cyclohexane/EtOAc 7:3 +1% AcOH) afforded compound **8a** as a white solid, which was recrystallized from *i*PrOH (836 mg, 2.59 mmol, 80% yield). [α]_D_^20^ = +169.2 (c = 0.1, CHCl_3_). ^1^H NMR (300 MHz, CDCl_3_) δ 5.45 (br, 1H), 5.00 (ddd, *J* = 11.0, 7.7, 3.9 Hz, 1H), 4.52 (dd, *J* = 8.0, 3.9, 1H), 3.30–3.60 (m, 2H), 1.45 (s, 9H). Proton of COOH not seen. ^13^C NMR (CDCl_3_) δ 172.2, 155.7, 138.6, 82.1, 81.5, 56.3, 44.3, 28.5 (3C). The analytical data were in agreement with the values obtained in the literature [13].

#### 3.1.11. (*S*)-2-((*R*)-3-Bromo-4,5-dihydroisoxazol-5-yl)-2-((*tert*-butoxycarbonyl)amino)acetic acid (**8b**)

Compound **8b** was prepared according to general procedure A from intermediate **7b** (700 mg, 2.26 mmol). Purification using silica gel column chromatography (cyclohexane/EtOAc 7:3 +1% AcOH) afforded compound **8b** as a white solid, which was recrystallized from *i*PrOH (643 mg, 1.99 mmol, 88% yield). [α]_D_^20^ = −95.0 (c = 0.1, CHCl_3_). ^1^H NMR (300 MHz, CDCl_3_) δ 5.31–5.22 (m, 2H), 4.58 (d, *J* = 9.3 Hz, 1H), 3.40 (dd, *J* = 17.7, 10.9 Hz, 1H), 3.25 (dd, *J* = 17.7, 7.7 Hz, 1H), 1.46 (s, 9H). Proton of COOH not seen. ^13^C NMR (75 MHz, Methanol-*d*_4_) δ 170.7, 157.1, 137.8, 81.4, 79.7, 55.8, 43.2, 27.2 (3C).

#### 3.1.12. (*R*)-2-((*S*)-3-Bromo-4,5-dihydroisoxazol-5-yl)-2-((*tert*-butoxycarbonyl)amino)acetic acid (**8c**)

Compound **8c** was prepared according to general procedure A from intermediate **7c** (700 mg, 2.26 mmol). Purification using silica gel column chromatography (cyclohexane/EtOAc 7:3 +1% AcOH) afforded compound **8c** as a white solid, which was recrystallized from *i*PrOH (614 mg, 1.90 mmol, 84% yield). [α]_D_^20^ = +98.0 (c = 0.1, CHCl_3_). The NMR data were consistent with those obtained for the corresponding enantiomer **8b**.

#### 3.1.13. (*R*)-2-((*R*)-3-Bromo-4,5-dihydroisoxazol-5-yl)-2-((*tert*-butoxycarbonyl)amino)acetic acid (**8d**)

Compound **8d** was prepared according to general procedure A from intermediate **7d** (1.00 g, 3.23 mmol). Purification using silica gel column chromatography (cyclohexane/EtOAc 7:3 +1% AcOH) afforded compound **8d** as a white solid, which was recrystallized from *i*PrOH (868 mg, 2.68 mmol, 83% yield). [α]_D_^20^ = −168.5 (c = 0.1, CHCl_3_). The NMR data were consistent with those obtained for the corresponding enantiomer **8a**.

#### 3.1.14. (*S*)-2-Amino-2-((*R*)-3-bromo-4,5-dihydroisoxazol-5-yl)acetic acid (**1b**)

Compound **1b** was prepared according to general procedure B from intermediate **8b** (200 mg, 0.62 mmol). **1b** was obtained as a white solid, which was recrystallized from MeOH/H_2_O (66 mg, 0.30 mmol, 48% yield). [α]_D_^20^ = −100.4 (c = 0.1, water). ^1^H NMR (300 MHz, D_2_O) δ 4.95 (ddd, *J* = 10.6, 7.5, 7.3 Hz, 1H), 3.77 (d, *J* = 7.3 Hz, 1H), 3.54 (dd, *J* = 18.3, 10.6 Hz, 1H), 3.40 (dd, *J* = 18.3, 7.5 Hz, 1H). Protons of NH_2_ and COOH not seen. ^13^C NMR (75 MHz, D_2_O) δ 170.6, 140.9, 79.5, 56.5, 44.5.

#### 3.1.15. (*R*)-2-Amino-2-((*S*)-3-bromo-4,5-dihydroisoxazol-5-yl)acetic acid (**1c**)

Compound **1c** was prepared according to general procedure B from intermediate **8c** (200 mg, 0.62 mmol). **1c** was obtained as a white solid, which was recrystallized from MeOH/H_2_O (99 mg, 0.45 mmol, 72% yield). [α]_D_^20^ = +100.7 (c = 0.1, water). The NMR data were consistent with those obtained for the corresponding enantiomer **1b**.

#### 3.1.16. (*R*)-2-Amino-2-((*R*)-3-bromo-4,5-dihydroisoxazol-5-yl)acetic acid (**1d**)

Compound **1d** was prepared according to general procedure B from intermediate **8d** (200 mg, 0.62 mmol). **1d** was obtained as a white solid, which was recrystallized from MeOH/H_2_O (101 mg, 0.45 mmol, 73% yield). [α]_D_^20^ = −170.4 (c = 0.1, water). ^1^H NMR (300 MHz, D_2_O) δ 5.15 (ddd, *J* = 11.0, 8.2, 3.3 Hz, 1H), 3.99 (d, *J* = 3.3 Hz, 1H), 3.52 (dd, *J* = 17.1, 11.0 Hz, 1H), 3.41 (dd, *J* = 17.1, 8.2 Hz, 1H). Protons of NH_2_ and COOH not seen. ^13^C NMR (75 MHz, D_2_O) δ 170.0, 140.9, 79.7, 56.1, 42.9. The NMR data were consistent with those reported in the literature for the corresponding enantiomer **1a** [13].

#### 3.1.17. Methyl (*S*)-2-((*R*)-3-bromo-4,5-dihydroisoxazol-5-yl)-2-((tert-butoxycarbonyl)amino)acetate (**9b**)

Compound **9b** was prepared according to general procedure C from intermediate **8b** (200 mg, 0.62 mmol). Purification using silica gel column chromatography (cyclohexane/EtOAc 8:2) afforded compound **9b** as a yellow oil (123 mg, 0.37 mmol, 59% yield). [α]_D_^20^ = −111.5 (c = 0.5, CHCl_3_). ^1^H NMR (300 MHz, CDCl_3_) δ 5.27–5.18 (m, 2H), 4.53 (dd, *J* = 9.4, 2.0 Hz, 1H), 3.81 (s, 3H), 3.36 (dd, *J* = 17.6, 10.8 Hz, 1H), 3.25 (dd, *J* = 17.6, 7.8 Hz, 1H), 1.46 (s, 9H). ^13^C NMR (75 MHz, CDCl_3_) δ 169.3, 156.2, 137.9, 81.4, 80. 8, 56.0, 53.0, 43.6, 28.2 (3C).

#### 3.1.18. Methyl (*R*)-2-((*S*)-3-bromo-4,5-dihydroisoxazol-5-yl)-2-((*tert*-butoxycarbonyl)amino)acetate (**9c**)

Compound **9c** was prepared according to general procedure C from intermediate **8c** (200 mg, 0.62 mmol). Purification using silica gel column chromatography (cyclohexane/EtOAc 8:2) afforded compound **9c** as a yellow oil (131 mg, 0.39 mmol, 63% yield). [α]_D_^20^ = +113.9 (c = 0.5, CHCl_3_). The NMR data were consistent with those obtained for the corresponding enantiomer **9b**.

#### 3.1.19. Methyl (*R*)-2-((*R*)-3-bromo-4,5-dihydroisoxazol-5-yl)-2-((*tert*-butoxycarbonyl)amino)acetate (**9d**)

Compound **9d** was prepared according to general procedure C from intermediate **8d** (200 mg, 0.62 mmol). Purification using silica gel column chromatography (cyclohexane/EtOAc 8:2) afforded compound **9d** as a yellow oil (125 mg, 0.37 mmol, 60% yield). [α]_D_^20^ = −156.4 (c = 0.5, CHCl_3_). ^1^H NMR (300 MHz, CDCl_3_) δ 5.50 (d, *J* = 8.0, 1H), 4.92 (ddd, *J* = 10.5, 7.2, 3.3 Hz, 1H), 4.42 (dd, *J* = 8.0, 3.3 Hz, 1H), 3.78 (s, 3H), 3.35–3.50 (m, 2H), 1.42 (s, 9H). ^13^C NMR (75 MHz, CDCl_3_) δ 169.3, 155.3, 138.2, 82.2, 80.9, 56.6, 53.2, 44.5, 28.4 (3C). The NMR data were consistent with those reported in the literature for the corresponding enantiomer **9a** [14].

#### 3.1.20. Benzyl (*S*)-2-((*R*)-3-bromo-4,5-dihydroisoxazol-5-yl)-2-((*tert*-butoxycarbonyl)amino)acetate (**10b**)

Compound **10b** was prepared according to general procedure D from intermediate **8b** (200 mg, 0.62 mmol). Purification using silica gel column chromatography (cyclohexane/EtOAc 9:1) afforded compound **10b** as a colorless oil (156 mg, 0.38 mmol, 61% yield). [α]_D_^20^ = −99.8 (c = 0.5, CHCl_3_). ^1^H NMR (300 MHz, CDCl_3_) δ 7.44–7.30 (m, 5H), 5.36–5.12 (m, 4H), 4.56 (dd, *J* = 9.5, 2.1 Hz, 1H), 3.34 (dd, *J* = 17.6, 10.7 Hz, 1H), 3.26 (dd, *J* = 17.6, 8.0 Hz, 1H), 1.45 (s, 9H). ^13^C NMR (75 MHz, CDCl_3_ δ 169.3, 155.6, 138.0, 135.5, 128.9 (2C), 128.7 (2C), 128.6, 82.4, 80.8, 68.1, 57.4, 44.8, 28.6 (3C).

#### 3.1.21. Benzyl (*R*)-2-((*S*)-3-bromo-4,5-dihydroisoxazol-5-yl)-2-((*tert*-butoxycarbonyl)amino)acetate (**10c**)

Compound **10c** was prepared according to general procedure D from intermediate **8c** (200 mg, 0.62 mmol). Purification using silica gel column chromatography (cyclohexane/EtOAc 9:1) afforded compound **10c** as a colorless oil (156 mg, 0.38 mmol, 61% yield). [α]_D_^20^ = +94.6 (c = 0.5, CHCl_3_). The NMR data were consistent with those obtained for the corresponding enantiomer **10b**.

#### 3.1.22. Benzyl (*R*)-2-((*R*)-3-bromo-4,5-dihydroisoxazol-5-yl)-2-((*tert*-butoxycarbonyl)amino)acetate (**10d**)

Compound **10d** was prepared according to general procedure D from intermediate **8d** (200 mg, 0.62 mmol). Purification using silica gel column chromatography (cyclohexane/EtOAc 9:1) afforded compound **10d** as a yellow oil (200 mg, 0.48 mol, 78% yield). [α]_D_^20^ = −136.2 (c = 0.5, CHCl_3_). ^1^H NMR (300 MHz, CDCl_3_) δ 7.30–7.40 (m, 5H), 5.55 (d, *J* = 6.0 Hz, 1H), 5.22 (d, *J* = 12.0 Hz, 1H), 5.14 (d, *J* = 12.0 Hz, 1H), 4.83 (ddd, *J* = 11.3, 6.9, 3.6 Hz, 1H), 4.50 (m, 1H), 3.42 (dd, *J* = 17.6, 6.9 Hz, 1H), 3.30 (dd, *J* = 17.6, 11.3 Hz, 1H), 1.42 (s, 9H). ^13^C NMR (75 MHz, CDCl_3_) δ 168.7, 155.3, 138.1, 134.9, 128.9 (2C), 128.9 (2C), 128.8, 82.3, 80.9, 68.3, 56.6, 44.4, 28.5 (3C). The NMR data were consistent with those reported in the literature for the corresponding enantiomer **10a** [14].

#### 3.1.23. *tert*-Butyl ((*S*)-2-(benzylamino)-1-((*R*)-3-bromo-4,5-dihydroisoxazol-5-yl)-2-oxoethyl)carbamate (**11b**)

Compound **11b** was prepared according to general procedure E from intermediate **8b** (200 mg, 0.62 mmol). Purification using silica gel column chromatography (cyclohexane/EtOAc 7:3) afforded compound **11b** as a colorless oil (176 mg, 0.43 mmol, 69% yield). [α]_D_^20^ = −97.7 (c = 1.0, CHCl_3_). ^1^H NMR (300 MHz, CDCl_3_) δ 7.42–7.20 (m, 5H), 6.60 (br, 1H), 5.43–5.19 (m, 2H), 4.57–4.27 (m, 3H), 3.37 (dd, *J* = 17.8, 11.1 Hz, 1H), 3.12 (dd, *J* = 17.8, 8.0 Hz, 1H), 1.44 (s, 9H). ^13^C NMR (75 MHz, CDCl_3_) δ 168.1, 155.8, 138.3, 137.4, 128.7 (2C), 127.7 (2C), 127.6, 80.8, 77.3, 56.2, 43.8, 43.6, 28.2 (3C).

#### 3.1.24. *tert*-Butyl ((*R*)-2-(benzylamino)-1-((*S*)-3-bromo-4,5-dihydroisoxazol-5-yl)-2-oxoethyl)carbamate (**11c**)

Compound **11c** was prepared according to general procedure E from intermediate **8c** (200 mg, 0.62 mmol). Purification using silica gel column chromatography (cyclohexane/EtOAc 7:3) provided compound **11c** as a colorless oil (219 mg, 0.53 mmol, 86% yield). [α]_D_^20^ = +100.1 (c = 1.0, CHCl_3_). The NMR spectral data were in agreement with those of compound **11b**.

#### 3.1.25. *tert*-Butyl ((*R*)-2-(benzylamino)-1-((*R*)-3-bromo-4,5-dihydroisoxazol-5-yl)-2-oxoethyl)carbamate (**11d**)

Compound **11d** was prepared according to general procedure E from intermediate **8d** (200 mg, 0.62 mmol). Purification using silica gel column chromatography (cyclohexane/EtOAc 7:3) afforded compound **11d** as a colorless oil (240 mg, 0.58 mmol, 94% yield). [α]_D_^20^ = −138.7 (c = 1.0, CHCl_3_). ^1^H NMR (300 MHz, CDCl_3_) δ 7.38–7.22 (m, 5H), 6.54 (t, *J* = 5.7 Hz, 1H), 5.48 (d, *J* = 7.8 Hz, 1H), 4.74 (ddd, *J* = 10.5, 8.3, 6.6 Hz, 1H), 4.48 (d, *J* = 5.7 Hz, 2H), 4.26 (dd, *J* = 8.3, 7.8 Hz, 1H), 3.52 (dd, *J* = 17.7, 6.6 Hz, 1H), 3.32 (dd, *J* = 17.7, 10.5 Hz, 1H), 1.44 (s, 9H). ^13^C NMR (75 MHz, CDCl_3_) δ 168.4, 155.9, 138.8, 137.4, 128.7 (2C), 127.6 (2C), 127.6, 81.8, 77.2, 55.7, 44.3, 43.8, 28.2 (3C). The NMR data were consistent with those reported in the literature for the corresponding enantiomer **11a** [16].

#### 3.1.26. Methyl (*S*)-2-amino-2-((*R*)-3-bromo-4,5-dihydroisoxazol-5-yl)acetate (**2b**)

Compound **2b** was prepared according to general procedure F from intermediate **9b** (100 mg, 0.30 mmol, 1.0 eq). Purification using silica gel column chromatography (100% EtOAc) afforded compound **2b** as a pale-yellow oil (49 mg, 0.20 mmol, 69% yield). [α]_D_^20^ = −87.0 (c = 0.5, CHCl_3_). ^1^H NMR (300 MHz, CDCl_3_) δ 5.07 (ddd, *J* = 10.7, 7.9, 3.4 Hz, 1H), 3.76 (s, 3H), 3.47 (d, *J* = 3.4 Hz, 1H), 3.40 (dd, *J* = 17.2, 7.9 Hz, 1H), 3.29 (dd, *J* = 17.2, 10.7 Hz, 1H), 1.70 (br, 2H). ^13^C NMR (75 MHz, CDCl_3_) δ 172.8, 137.9, 82.2, 57.2, 52.6, 44.0.

#### 3.1.27. Methyl (*R*)-2-amino-2-((*S*)-3-bromo-4,5-dihydroisoxazol-5-yl)acetate (**2c**)

Compound **2c** was prepared according to general procedure F from intermediate **9c** (100 mg, 0.30 mmol, 1.0 eq). Purification using silica gel column chromatography (100% EtOAc) afforded compound **2c** as a pale-yellow oil (38 mg, 0.16 mmol, 54% yield). [α]_D_^20^ = +184.3 (c = 0.5, CHCl_3_). The NMR data were consistent with those obtained for the corresponding enantiomer **2b**.

#### 3.1.28. Methyl (*R*)-2-amino-2-((*R*)-3-bromo-4,5-dihydroisoxazol-5-yl)acetate (**2d**)

Compound **2d** was prepared according to general procedure F from intermediate **9d** (100 mg, 0.30 mmol, 1.0 eq). Purification using silica gel column chromatography (100% EtOAc) afforded compound **2d** as a colorless oil (42 mg, 0.18 mmol, 60% yield). [α]_D_^20^ = −97.1 (c = 0.5, CHCl_3_). ^1^H NMR (300 MHz, CDCl_3_) δ 4.97 (ddd, *J* = 10.7, 8.0, 3.5 Hz, 1H), 3.90 (d, *J* = 3.5 Hz, 1H), 3.75 (s, 3H), 3.31 (dd, *J* = 17.3, 8.0 Hz, 1H), 3.19 (dd, *J* = 17.3, 10.7 Hz, 1H), 1.60 (br, 2H). ^13^C NMR (75 MHz, CDCl_3_) δ 171.7, 137.9, 82.6, 56.0, 52.5, 42.3. The NMR data were consistent with those reported in the literature for the corresponding enantiomer **2a** [14].

#### 3.1.29. Benzyl (*S*)-2-amino-2-((*R*)-3-bromo-4,5-dihydroisoxazol-5-yl)acetate (**3b**)

Compound **3b** was prepared according to general procedure F from intermediate **10b** (100 mg, 0.24 mmol, 1.0 eq). Purification using silica gel column chromatography (100% EtOAc) afforded compound **3b** as an orange oil (45 mg, 0.14 mmol, 59% yield). [α]_D_^20^ = −121.0 (c = 0.5, CHCl_3_). ^1^H NMR (300 MHz, CDCl_3_) δ 7.42–7.33 (m, 5H), 5.23 (s, 2H), 5.14 (ddd, *J* = 10.9, 7.9, 3.4 Hz, 1H), 3.53 (d, *J* = 3.4 Hz, 1H), 3.43 (dd, *J* = 17.2, 7.9 Hz, 1H), 3.29 (dd, *J* = 17.2, 10.9 Hz, 1H), 1.64 (br, 2H). ^13^C NMR (75 MHz, CDCl_3_) δ 172.3, 137.9, 135.2, 128.7 (2C), 128.6, 128.4 (2C), 82.2, 67.5, 57.4, 44.0.

#### 3.1.30. Benzyl (*R*)-2-amino-2-((*S*)-3-bromo-4,5-dihydroisoxazol-5-yl)acetate (**3c**)

Compound **3c** was prepared according to general procedure F from intermediate **10c** (100 mg, 0.24 mmol, 1.0 eq). Purification using silica gel column chromatography (100% EtOAc) afforded compound **3c** as an orange oil (42 mg, 0.13 mmol, 55% yield). [α]_D_^20^ **=** +122.9 (c = 0.5, CHCl_3_). The NMR data were consistent with those obtained for the corresponding enantiomer **3b**.

#### 3.1.31. Benzyl (*R*)-2-amino-2-((*R*)-3-bromo-4,5-dihydroisoxazol-5-yl)acetate (**3d**)

Compound **3d** was prepared according to general procedure F from intermediate **10d** (100 mg, 0.24 mmol, 1.0 eq). Purification using silica gel column chromatography (100% EtOAc) afforded compound **3d** as an orange oil (39 mg, 0.12 mmol, 51% yield). [α]_D_^20^ = −62.0 (c = 0.5, CHCl_3_). ^1^H NMR (300 MHz, CDCl_3_) δ 7.43–7.33 (m, 5H), 5.22 (d, *J* = 12.0 Hz, 1H), 5.18 (d, *J* = 12.0 Hz, 1H), 5.00 (ddd, *J* = 10.7, 8.0, 3.9 Hz, 1H), 3.98 (d, *J* = 3.9 Hz, 1H), 3.31 (dd, *J* = 17.3, 8.0 Hz, 1H), 3.13 (dd, *J* = 17.3, 10.7 Hz, 1H), 1.86 (br, 2H). ^13^C NMR (75 MHz, CDCl_3_) δ 171.1, 137.9, 135.0, 128.8 (2C), 128.7, 128.5 (2C), 82.5, 67.4, 56.1, 42.2. The NMR data were consistent with those reported in the literature for the corresponding enantiomer **3a** [14].

#### 3.1.32. (*S*)-2-Amino-*N*-benzyl-2-((*R*)-3-bromo-4,5-dihydroisoxazol-5-yl)acetamide (**4b**)

Compound **4b** was prepared according to general procedure F from intermediate **11b** (150 mg, 0.36 mmol, 1 eq). Purification using silica gel column chromatography (100% EtOAc) afforded compound **4b** as a white solid (66 mg, 0.21 mmol, 58% yield). m.p. = 122–123 °C. [α]_D_^20^ = −112.7 (c = 1.0, CHCl_3_). ^1^H NMR (300 MHz, CDCl_3_) δ 7.70 (br, 1H), 7.38–7.21 (m, 5H), 5.00 (dt, *J* = 9.4, 5.9 Hz, 1H), 4.52–4.39 (m, 2H), 3.51 (d, *J* = 5.9 Hz, 1H), 3.38 (d, *J* = 9.4 Hz, 2H), 1.86 (br, 2H). ^13^C NMR (75 MHz, CDCl_3_) δ 171.1, 138.4, 138.0, 128.7 (2C), 127.6 (2C), 127.5, 82.5, 57.6, 44.2, 43.3.

#### 3.1.33. (*R*)-2-Amino-*N*-benzyl-2-((*S*)-3-bromo-4,5-dihydroisoxazol-5-yl)acetamide (**4c**)

Compound **4c** was prepared according to general procedure F from intermediate **11c** (150 mg, 0.36 mmol, 1 eq). Purification using silica gel column chromatography (100% EtOAc) provided compound **4c** as a white solid (61 mg, 0.20 mmol, 54% yield). [α]_D_^20^ = +110.2 (c = 1.0, CHCl_3_). The NMR data were consistent with those obtained for the corresponding enantiomer **4b**.

#### 3.1.34. (*R*)-2-Amino-*N*-benzyl-2-((*R*)-3-bromo-4,5-dihydroisoxazol-5-yl)acetamide (**4d**)

Compound **4d** was prepared according to general procedure F from intermediate **11d** (150 mg, 0.36 mmol, 1 eq). Purification using silica gel column chromatography (100% EtOAc) and recrystallization from iPrOH/n-hexane afforded compound **4c** as colorless needles (69 mg, 0.22 mmol, 61% yield). [α]_D_^20^ = −79.0 (c = 1.0, CHCl_3_). ^1^H NMR (300 MHz, CDCl_3_) δ 7.41–7.23 (m, 6H), 5.09 (dt, *J* = 9.6, 4.8 Hz, 1H), 4.44 (d, *J* = 6.3 Hz, 2H), 3.80 (d, *J* = 4.8 Hz, 1H), 3.22 (d, *J* = 9.6 Hz, 2H), 1.49 (br, 2H). ^13^C NMR (75 MHz, CDCl_3_) δ 170.6, 138.1, 137.8, 128.8 (2C), 127.7 (2C), 127.7, 82.8, 56.4, 43.3, 41.8. The NMR data were consistent with those reported in the literature for the corresponding enantiomer **4a** [16].

### 3.2. Molecular Modeling

Molecular modeling calculations were performed on the CPU/GPU hybrid High-Performance Computing Cluster (10 Twin servers, for a total of 560 Intel^®^ Xeon^®^ Gold processors (128 GB RAM), 64 AMD^®^ EPYC^®^ processors and 2 GPU NVIDIA^®^ Tesla^®^ V100) and on the High-Performance Computing Cluster (6 Twin servers for a total of 12 nodes each equipped with Intel^®^ Xeon^®^ QuadCore E5520 CPU, 36 GB RAM). The molecular modeling graphics were carried out on a personal computer equipped with an Intel (R) Core (TM) i7-8700 processor and SGI Octane 2 workstations.

The apparent pKa and clogD values (pH 7.4) of compounds **1a**–**1d**, **2a**–**2d**, **3a**–**3d**, and **4a**–**4d** were calculated using the ACD/pKa GALAS algorithm of ACD/Percepta software (ACD/Percepta, Advanced Chemistry Development, Inc., Toronto, ON, Canada, 2017, http://www.acdlabs.com (accessed on 5 September 2022). Then, the percentage of neutral/ionized forms was computed at pH 7.2 (cytoplasm pH value) using the Handerson−Hasselbalch equation.

#### 3.2.1. Conformational Analysis

The molecular models of the new compounds of **1a**–**1d**, **2b**–**2d**, **3b**–**3d**, and **4b**–**4d** were built (Small Molecule tool of Discovery Studio 2017; Dassault Systèmes BIOVIA, San Diego, CA, USA, 2017), atomic potentials and charges were assigned using the CFF forcefield [27]. The resulting structures were subjected to molecular mechanic (MM) energy minimization (ε = 80 * r) until the maximum RMS derivative was less than 0.001 kcal/Å, using the conjugate gradient as the minimization algorithm [28]. The obtained conformers were used as starting structures for the subsequent systematic conformational analysis (Search Small Molecule Conformations; Discovery Studio 2017). The conformational space was sampled by systematically varying the rotatable bonds sp3-sp3 and sp3-sp2 with an increment of 60°. The RMSD cutoff for the structure selection was set to 0.01 (Å). Finally, to ensure a wide variance of the input structures to be successively fully minimized, an energy threshold value of 10^6^ kcal/mol was used as the selection criteria. The generated structures were then subjected to MM energy minimization (CFF forcefield; ε = 80 * r) until the maximum RMS derivative was less than 0.001 kcal/Å, using the conjugate gradient as the minimization algorithm. Finally, the resulting conformers were ranked by their potential energy values (i.e., ΔE from the global energy minimum). The conformers within 5 kcal/mol from the global minimum were classified on the basis of dihedral angle values.

#### 3.2.2. Docking Studies

Docking calculations were performed by using as a protein structure the previously developed atomic model of *Pf*GAPDH [16]. Although in the docking simulation all the systems were perturbed by a Monte Carlo/minimization procedure, the docking procedure formally requires a reasonable starting structure. In order to define the starting conformation of the new compounds, all the conformers within 5 kcal/mol from the global minimum were placed in the GAPDH catalytic site considering the two binding modes of the glyceraldehyde 3-phosphate analogue 2-(2-phosphono-ethyl)-acrylic acid 4-nitro-phenyl ester (PDB ID: 1ML3). The conformations with the lowest potential energy that did not show a significant steric overlap with catalytic-site amino acids were selected as the starting conformations for the docking calculations.

A docking procedure, which considers all the systems as flexible (i.e., ligand and protein), was applied. Flexible docking was achieved using the Affinity module in the Insight 2005 suite, setting the SA_Docking procedure [29] and using the cell multipole method for non-bonded interactions [30]. The docking procedure included a Monte-Carlo(MC)-based conformational search of the ligand within the active site of *Pf*GAPDH. During the first step, starting from the previously obtained roughly docked structures, the ligand was moved by a random combination of translation, rotation, and torsional changes to sample both the conformational space of the ligand and its orientation with respect to the protein (MxRChange = 3 Å; MxAngChange = 180°). During this step, the van der Waals (vdW) and Coulombic terms were scaled to a factor of 0.1 to avoid very severe divergences in the vdW and Coulombic energies. If the energy of a complex structure resulting from the random moves of the ligand was higher by the energy tolerance parameter than the energy of the last accepted structure, it was not accepted for minimization. To ensure a wide variance of the input structures to be successively minimized, an energy tolerance value of 10^6^ kcal/mol from the previous structure was used. After the energy minimization step (conjugate gradient; 2500 iterations; ε = 1), the energy test, with an energy range of 50 kcal/mol, and a structure similarity check (rms tolerance = 0.3 kcal/Å) was applied to select the 20 acceptable structures. Each subsequent structure was generated from the last accepted structure.

All *Pf*GAPDH atoms were left free to move during the entire course of the docking calculations, whereas, in order to avoid unrealistic results, a tethering restraint was applied on the structurally conserved regions (SCRs) of the protein. To identify the SCRs, the *Pf*GAPDH sequence was analyzed using the structure prediction and sequence analysis server PredictProtein (http://www.predictprotein.org/ (accessed on 12 September 2022). In *Pf*GAPDH, 8 α-helix and 16 β-sheet secondary structures were predicted to be highly conserved (α1, aa13−23; α2, aa40−48; α3, aa105−113; α4, aa155−167; α5, aa198−207; α6, aa214−227; α7, aa258–270; α8, aa323–335 β1, aa4–8; β2, aa29–34; β3, aa58−62; β4, aa66−70; β5, aa73−79; β6, aa93–98; β7, aa118–123; β8, aa131–136; β9, aa146–149; β10, aa171–182; β11, aa234–238; β12, aa244–252; β13, aa275–278; β14, aa296–299; β15, aa303–306; β16, aa310–317). Within the identified SCRs, the distance between the backbone hydrogen bond donors and acceptors in the α-helices was restrained within 2.5 Å. On the other hand, the φ and ψ torsional angles of the β-sheets were restrained within −119° and +113°, or −139° and +135°, respectively, according to the parallel or anti-parallel structure. According to the reliability index values obtained from the secondary structure prediction analysis, we applied restraints with a quadratic form and the following set of force constants: (i) 1 kcal/mol/Å^2^ (maximum force: 10 kcal/mol/Å^2^) for reliability index values from 0 to 3, (ii) 10 kcal/mol/Å^2^ (maximum force: 100 kcal/mol/Å^2^) for reliability index values from 4 to 6, and (iii) 100 kcal/mol/Å^2^ (maximum force: 1000 kcal/mol/Å^2^) for reliability index values from 7 to 9. Moreover, in order to investigate the first approach of our compounds to the catalytic site before the nucleophilic attack, a tethering restraint was applied on: (i) the hydrogen bond between the catalytic residues C153 and H180 (constrained within 2.5 Å using a force constant of 100 (kcal/mol)/Å) and (ii) the distance between the electrophilic carbon of the 3-bromo-4,5-dihydroisoxazole ring and the sulfur atom of C153 (constrained within 3.4 Å using a force constant of 100 (kcal/mol)/Å according to the data present in the literature) [31,32].

For each compound, the resulting complexes were superimposed on the starting structure by fitting all the Cα atoms, and the Cα RMSD of each residue and its average value were calculated. Then, the complexes were again superimposed on the starting structure by fitting the Cα atoms of the residues characterized by an average value of RMSD ≤ 0.2 Å. Considering this latter superimposition, the Cα RMSD of the catalytic residues and the RMSD of NAD^+^ were calculated. The χ1 torsion angle of C153 and the geometric criteria of the hydrogen bond between C153 and H180 were also evaluated for each generated complex. In particular, the angle D-H-A and X-D-A of this hydrogen bond was calculated, assuming as D the sulfur atom of C153, as A the Nτ hydrogen atom of H180, and as X the Cβ of C153.

Finally, for each generated complex, the non-bonded interaction energy (vdW and electrostatic energy contribution; group-based method [33]; CUT_OFF = 100; ε = 2 * r; Discover_3 Module of Insight 2005) was calculated. In our previous publication, the docked complexes of **2a**, **3a**, and **4a** were not analyzed using these criteria [16]. Accordingly, to properly compare the results obtained for new diastereoisomers with those obtained with **2a**, **3a**, and **4a**, the latter were also included in this analysis.

For each docking calculation, the complex with the most favorable interaction energy was characterized by (i) the Cα RMSD of C152 and H179 with respect to the starting structure ≤ 3 Å; (ii) the gauche(-) conformation (from −30° to −90°) of the torsion angle χ1 of C153 (i.e., the conformation needed to establish the hydrogen bond with H180); and (iii) the angles of the hydrogen bond between C153 and H180 > 90° [34] were selected.

The selected docked complexes were then subjected to MM energy minimization, applying only the restraint on the hydrogen bond between the catalytic residues C153 and H180 (RMS derivative < 0.5 kcal/Å; Steepest Descent algorithm; ε = 80 * r; Module Discover; Insight 2005). The optimized complexes were again filtered by using, in addition to the above reported criteria, the distance between the electrophilic carbon C3 of the 3-bromo-4,5-dihydroisoxazole ring and the sulfur atom of C153 ≤ 4 Å.

The complex with the most favorable interaction energy meeting the filtering criteria was chosen as the structure representing the most probable calculated approach of the compounds to the catalytic cysteine of *Pf*GAPDH. The quality of the selected docked complexes was checked using Procheck structure evaluator software [35].

### 3.3. Biological Assays

#### 3.3.1. Expression and Purification of *Pf*GAPDH

Recombinant His-tagged *Pf*GAPDH was produced in *Escherichia coli*, as has already been described [14].

#### 3.3.2. Enzyme Assays

The GAPDH activity was evaluated using a modified version of the Ferdinand assay in a buffered solution containing 10 mM of TEA, 10 mM of sodium arsenate, 5 mM of EDTA, 1.5 mM of NAD^+^, and 2.2 of mM DL-glyceraldehyde 3-phosphate, as has already been described [14]. GAPDH was added at a final concentration of 33 nM, and the NADH formation was monitored at 340 nm using a Cary4000 spectrophotometer (Agilent Technologies) with the cell holder maintained at 25 °C.

#### 3.3.3. Parasite Growth and Drug Susceptibility Assay

The CQ-sensitive (D10) and CQ-resistant (W2) strains of *P. falciparum* were sustained in vitro, as described by Trager and Jensen [36,37]. All the strains were cultured at 5% hematocrit (human type A-positive red blood cells) in RPMI 1640 (EuroClone, Celbio) medium with the addition of 1% AlbuMax (Invitrogen, Milan, Italy), 0.01% hypoxanthine, 20 mM of Hepes Buffer, and 2 mM of glutamine. Parasites were maintained at 37 °C in a standard gas mixture consisting of 1% O_2_, 5% CO_2_, and 94% N_2_. For the drug sensitivity assay, the compounds were dissolved in DMSO and then diluted with medium to achieve the required concentrations (final DMSO concentration < 1%, which is nontoxic to the parasite). The drugs were placed in 96-well flat-bottom microplates (COSTAR), and serial dilutions were made. Asynchronous cultures with a parasitemia of 1–1.5% and 1% final hematocrit were added into the plates and incubated for 72 h at 37 °C. The parasite growth was determined spectrophotometrically (OD650) by measuring the activity of the parasite lactate dehydrogenase (pLDH), according to a modified version of Makler’s method in control and drug-treated cultures [38]. The antiplasmodial activity was expressed as 50% inhibitory concentrations (IC_50_). Each IC_50_ value is the mean ± standard deviation of at least three separate experiments performed in duplicate.

## 4. Conclusions

Herein, we investigated the importance of stereochemistry for the biological activity of nature-inspired 3-Br-acivicin and its derivatives. For each molecule, the four isomers were prepared and evaluated in vitro towards *P. falciparum*. Only the (5*S*, α*S*) natural isomers showed a significant antimalarial activity, suggesting that their uptake might be mediated by the L-amino acid transport system. Considering the *Pf*GAPDH inhibition, the most potent compounds turned out to be the ester derivatives **2a**–**2d** and **3a**–**3d**, with no significant differences among the diastereoisomers. In contrast, stereochemistry affected the target binding for the other two subclasses (**1a**–**1d** and **4a**–**4d**). A clear correlation between the *Pf*GAPDH inhibitory activity and the antimalarial potency was not observed; therefore, additional targets might be involved in the biological effects. The racemic form of the 3-bromo-4,5-dihydroisoxazole scaffold had been exploited in chemoproteomic profiling methods to engage reactive cysteine residues in the human proteome [39]. Our enantiomerically pure 3-bromo-4,5-dihydroisoxazole derivatives represent valuable tools for proteomic experiments on malaria parasite *P. falciparum*, aiming to reveal new ligandable sites in proteins and to guide a future structure-based drug design.

## Figures and Tables

**Table 1:** Chemical structures, clogD, and IC_50_s against *P. falciparum* D10 and W2 strains.

Structure	Compound	clogD ^a^	*P. falciparum* D10 IC_50_ (µM) ^b^	*P. falciparum* W2 IC_50_ (µM) ^b^
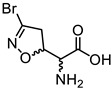	(5*S*, α*S*)-**1a**	0.02	0.35 ± 0.08	0.34 ± 0.12
	(5*R*, α*S*)-**1b**		23.54 ± 0.31	24.75 ± 0.90
	(5*S*, α*R*)-**1c**		7.49 ± 1.48	8.47 ± 2.06
	(5*R*, α*R*)-**1d**		8.79 ± 1.12	10.18 ± 1.75
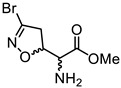	(5*S*, α*S*)-**2a**	0.04	0.79 ± 0.21	0.88 ± 0.23
	(5*R*, α*S*)-**2b**		17.14 ± 5.91	17.18 ± 7.39
	(5*S*, α*R*)-**2c**		40.67 ± 16.20	48.18 ± 14.81
	(5*R*, α*R*)-**2d**		8.27 ± 1.05	7.42 ± 3.54
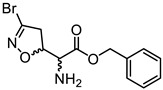	(5*S*, α*S*)-**3a**	1.41	0.37 ± 0.12	0.26 ± 0.05
	(5*R*, α*S*)-**3b**		32.65 ± 18.10	34.85 ± 5.30
	(5*S*, α*R*)-**3c**		19.35 ± 4.34	16.51 ± 8.11
	(5*R*, α*R*)-**3d**		4.31 ± 0.51	4.50 ± 1.82
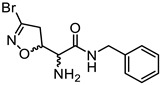	(5*S*, α*S*)-**4a**	0.76	0.36 ± 0.11	0.48 ± 0.18
	(5*R*, α*S*)-**4b**		18.20 ± 1.15	18.20 ± 0.93
	(5*S*, α*R*)-**4c**		19.35 ± 2.15	19.35 ± 3.36
	(5*R*, α*R*)-**4d**		8.55 ± 1.95	8.91 ± 3.40

^a^ The clogD values (pH 7.4) were calculated by using ACD/pKa GALAS algorithm of ACD/Percepta software (ACD/Percepta, Advanced Chemistry Development, Inc., Toronto, ON, Canada, 2017, http://www.acdlabs.com (accessed on 5 September 2022). ^b^ pLDH method; data are the mean of three independent experiments ± SD; D10: CQ-susceptible *P. falciparum* strain; W2: CQ-resistant *P. falciparum* strain.

## Data Availability

Not applicable.

## Supplementary Materials

The following supporting information can be downloaded at: https://www.mdpi.com/article/10.3390/molecules28073172/s1. Table S1: clogD7.4, pKa values and percentage of ionic forms at pH 7.2; Tables S2–S38: Experimental data from molecular modelling studies; Figures S1–S10: Visual representation of the docking results; ^1^H and ^13^C NMR spectra of final compounds.
