# Supplementary material for: Role of Stereochemistry on the Biological Activity of Nature-Inspired 3-Br-Acivicin Isomers and Derivatives
Source: Molecules. 2023 Apr 3;28(7):3172. doi: 10.3390/molecules28073172 (PMC10095986; doi:10.3390/molecules28073172)

SUPPORTING INFORMATION

Role of Stereochemistry on the Biological Activity of Nature-Inspired 3-Br-Acivicin Isomers and Derivatives

Andrea Galbiati [1,†], Aureliano Zana [1,†], Chiara Borsari [1], Marco Persico [2], Stefania Bova [3], Oleh Tkachuk [2], Alexandra Ioana Corfu [1], Lucia Tamborini [1], Nicoletta Basilico [4], Caterina Fattorusso [2], Stefano Bruno [5], Silvia Parapini [6] and Paola Conti [1,*]

Table of Content

Table S1. clog$D_{7.4}$, pK_a values and percentage of ionic forms at pH 7.2....S4

Table S2. ΔE_{GM} values (kcal/mol) and torsional angle values (degrees) of **1a** considering MM conformers within 5 kcal/mol from the global minimum....S5

Table S3. ΔE_{GM} values (kcal/mol) and torsional angle values (degrees) of **1b** considering MM conformers within 5 kcal/mol from the global minimum....S6

Table S4. ΔE_{GM} values (kcal/mol) and torsional angle values (degrees) of **1c** considering MM conformers within 5 kcal/mol from the global minimum....S7

Table S5. ΔE_{GM} values (kcal/mol) and torsional angle values (degrees) of **1d** considering MM conformers within 5 kcal/mol from the global minimum....S8

Table S6. ΔE_{GM} values (kcal/mol) and torsional angle values (degrees) of **2b** considering MM conformers within 5 kcal/mol from the global minimum....S9

Table S7. ΔE_{GM} values (kcal/mol) and torsional angle values (degrees) of **2c** considering MM conformers within 5 kcal/mol from the global minimum....S10

Table S8. ΔE_{GM} values (kcal/mol) and torsional angle values (degrees) of **2d** considering MM conformers within 5 kcal/mol from the global minimum....S11

Table S9. ΔE_{GM} values (kcal/mol) and torsional angle values (degrees) of **3b** considering MM conformers within 5 kcal/mol from the global minimum....S12

Table S10. ΔE_{GM} values (kcal/mol) and torsional angle values (degrees) of **3c** considering MM conformers within 5 kcal/mol from the global minimum....S13

Table S11. ΔE_{GM} values (kcal/mol) and torsional angle values (degrees) of **3d** considering MM conformers within 5 kcal/mol from the global minimum....S14

Table S12. ΔE_{GM} values (kcal/mol) and torsional angle values (degrees) of **4b** considering MM conformers within 5 kcal/mol from the global minimum....S15

Table S13. ΔE_{GM} values (kcal/mol) and torsional angle values (degrees) of **4c** considering MM conformers within 5 kcal/mol from the global minimum....S16

Table S14. ΔE_{GM} values (kcal/mol) and torsional angle values (degrees) of **4d** considering MM conformers within 5 kcal/mol from the global minimum....S17

Table S15. Non-bond interaction energies (kcal/mol), Cα RMSD (Å) of C153 and H180 calculated with respect to the starting position. χ1 torsion angle of C153 (degrees) and angle values of the hydrogen bond between C153 and H180 (degrees) of the **1a**/*Pf*GAPDH complexes obtained by Monte Carlo/Minimization procedure....S18

Table S16. Non-bond interaction energies (kcal/mol), Cα RMSD (Å) of C153 and H180 calculated with respect to the starting position. χ1 torsion angle of C153 (degrees) and angle values of the hydrogen bond between C153 and H180 (degrees) of the **1b**/*Pf*GAPDH complexes....S19

Table S17. Non-bond interaction energies (kcal/mol), Cα RMSD (Å) of C153 and H180 calculated with respect to the starting position. χ1 torsion angle of C153 (degrees) and angle values of the hydrogen bond between C153 and H180 (degrees) of the **1c**/*Pf*GAPDH complexes......S20

Table S18. Non-bond interaction energies (kcal/mol), Cα RMSD (Å) of C153 and H180 calculated with respect to the starting position. χ1 torsion angle of C153 (degrees) and angle values of the hydrogen bond between C153 and H180 (degrees) of the **1d**/*Pf*GAPDH complexes......S21

Table S19. Non-bond interaction energies (kcal/mol), Cα RMSD (Å) of C153 and H180 calculated with respect to the starting position. χ1 torsion angle of C153 (degrees) and angle values of the hydrogen bond between C153 and H180 (degrees) of the **2a**/*Pf*GAPDH complexes......S22

Table S20. Non-bond interaction energies (kcal/mol), Cα RMSD (Å) of C153 and H180 calculated with respect to the starting position. χ1 torsion angle of C153 (degrees) and angle values of the hydrogen bond between C153 and H180 (degrees) of the **2b**/*Pf*GAPDH complexes......S23

Table S21. Non-bond interaction energies (kcal/mol), Cα RMSD (Å) of C153 and H180 calculated with respect to the starting position. χ1 torsion angle of C153 (degrees) and angle values of the hydrogen bond between C153 and H180 (degrees) of the **2c**/*Pf*GAPDH complexes......S24

Table S22. Non-bond interaction energies (kcal/mol), Cα RMSD (Å) of C153 and H180 calculated with respect to the starting position. χ1 torsion angle of C153 (degrees) and angle values of the hydrogen bond between C153 and H180 (degrees) of the **2d**/*Pf*GAPDH complexes......S25

Table S23. Non-bond interaction energies (kcal/mol), Cα RMSD (Å) of C153 and H180 calculated with respect to the starting position. χ1 torsion angle of C153 (degrees) and angle values of the hydrogen bond between C153 and H180 (degrees) of the **3a**/*Pf*GAPDH complexes......S26

Table S24. Non-bond interaction energies (kcal/mol), Cα RMSD (Å) of C153 and H180 calculated with respect to the starting position. χ1 torsion angle of C153 (degrees) and angle values of the hydrogen bond between C153 and H180 (degrees) of the **3b**/*Pf*GAPDH complexes......S27

Table S25. Non-bond interaction energies (kcal/mol), Cα RMSD (Å) of C153 and H180 calculated with respect to the starting position. χ1 torsion angle of C153 (degrees) and angle values of the hydrogen bond between C153 and H180 (degrees) of the **3c**/*Pf*GAPDH complexes......S28

Table S26. Non-bond interaction energies (kcal/mol), Cα RMSD (Å) of C153 and H180 calculated with respect to the starting position. χ1 torsion angle of C153 (degrees) and angle values of the hydrogen bond between C153 and H180 (degrees) of the **3d**/*Pf*GAPDH complexes......S29

Table S27. Non-bond interaction energies (kcal/mol), Cα RMSD (Å) of C153 and H180 calculated with respect to the starting position. χ1 torsion angle of C153 (degrees) and angle values of the hydrogen bond between C153 and H180 (degrees) of the **4a**/*Pf*GAPDH complexes......S30

Table S28. Non-bond interaction energies (kcal/mol), Cα RMSD (Å) of C153 and H180 calculated with respect to the starting position. χ1 torsion angle of C153 (degrees) and angle values of the hydrogen bond between C153 and H180 (degrees) of the **4b**/*Pf*GAPDH complexes......S31

Table S29. Non-bond interaction energies (kcal/mol), Cα RMSD (Å) of C153 and H180 calculated with respect to the starting position. χ1 torsion angle of C153 (degrees) and angle values of the hydrogen bond between C153 and H180 (degrees) of the **4c**/*Pf*GAPDH complexes......S32

Table S30. Non-bond interaction energies (kcal/mol), Cα RMSD (Å) of C153 and H180 calculated with respect to the starting position. χ1 torsion angle of C153 (degrees) and angle values of the hydrogen bond between C153 and H180 (degrees) of the **4d**/*Pf*GAPDH complexes......S33

Table S31. Non-bond interaction energies (kcal/mol), Cα RMSD (Å) of C153 and H180 calculated with respect to the starting position, χ1 torsion angle of C153(degrees), and angles of the hydrogen bond between C153 and H180 (degrees) of the best docked complexes after unrestrained structure optimization......S34

Table S32. Summary of Procheck results obtained for the selected docked complexes......................................S35

Table S33. Calculated RMSD (Å) of the ligand conformation in the docked complexes with respect to the conformers obtained by the conformational analysis. The energy difference (ΔE) from the global minimum energy conformer (GM) is also reported...S36

Table S34. Solvent accessible surface area (SASA) of bromine for the selected docked complexes....................S37

Table S35. Ligand-residue interactions of the *Pf*GAPDH/**1a**, *Pf*GAPDH/**1b**, *Pf*GAPDH/**1c**, and *Pf*GAPDH/**1d** docked complexes...S38

Table S36. Ligand-residue interactions of the *Pf*GAPDH/**2a**, *Pf*GAPDH/**2b**, *Pf*GAPDH/**2c**, and *Pf*GAPDH/**2d** docked complexes...S39

Table S37. Ligand-residue interactions of the *Pf*GAPDH/**3a**, *Pf*GAPDH/**3b**, *Pf*GAPDH/**3c**, and *Pf*GAPDH/**3d** docked complexes...S40

Table S38. Ligand-residue interactions of the *Pf*GAPDH/**4a**, *Pf*GAPDH/**4b**, *Pf*GAPDH/**4c**, and *Pf*GAPDH/**4d** docked complexes...S41

Figure S1. Docked complexes of **2a** (orange), **2b** (yellow), **2c** (green) and **2d** (magenta) bound to P_I site of PfGAPDH...S42

Figure S2. Docked complexes of **2a** (orange), **2b** (yellow), **2c** (green) and **2d** (magenta) bound to P_{II} site of PfGAPDH...S43

Figure S3. Docked complexes of **3a** (orange), **3b** (yellow), **3c** (green) and **3d** (magenta) bound to P_I site of PfGAPDH...S44

Figure S4. Docked complexes of **3a** (orange), **3b** (yellow), **3c** (green) and **3d** (magenta) bound to P_{II} site of PfGAPDH……………………………………………………………………………………………..S45

Figure S5. Docked complexes of **1a** (orange), **1b** (yellow), **1c** (green) and **1d** (magenta) bound to P_I site of *Pf*GAPDH...S46

Figure S6. Docked complexes of **1a** (orange), **1b** (yellow), **1c** (green) and **1d** (magenta) bound to P_{II} site of *Pf*GAPDH...S47

Figure S7. Docked complexes of **4a** (orange), **4b** (yellow), **4c** (green) and **4d** (magenta) bound to P_I site of PfGAPDH...S48

Figure S8. Docked complexes of **4a** (orange), **4b** (yellow), **4c** (green) and **4d** (magenta) bound to P_{II} site of PfGAPDH...S49

Figure S9. Superimposition of the two **4c** conformations obtained from docking studies on the corresponding local energy minimum conformer obtained from conformational analysis...S50

Figure S10. Superimposition of the two **4c** conformations obtained from docking studies on the global energy minimum conformer obtained from the conformational analysis...S51

^{1}H and ^{13}C NMR spectra of final compounds...S52

Table S1. $clogD_{7.4}$, pK_a values and percentage of ionic forms at pH 7.2.

Compd	$clogD_{7.4}$	pK_a	Prevalent Ionic Form (%)
**1a-1d**	0.02	2.0 ± 0.4<br>9.3 ± 0.4	ZW(100)
**2a-2d**	0.04	7.2 ± 0.4	N(49)<br>P(51)
**3a-3d**	1.41	7.3 ± 0.4	N(47)<br>P(53)
**4a-4d**	0.76	7.4 ± 0.4<br>12.1 ± 1.0	N (39)<br>P(61)

Table S2. ΔE_{GM} values (kcal/mol) and torsional angle values (degrees) of **1a** considering MM conformers within 5 kcal/mol from the global minimum.

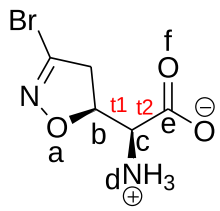

**Frame**	**ΔE_{GM} (kcal/mol)**	**Torsional Angles (°)**	
		**τ1**[a]	**τ2**[b]
1	0	80	2
2	0.41	176	-8
3	1.51	-62	2

[a]τ1: abcd; [b]τ2: dcef

Table S3. ΔE_{GM} values (kcal/mol) and torsional angle values (degrees) of **1b** considering MM conformers within 5 kcal/mol from the global minimum.

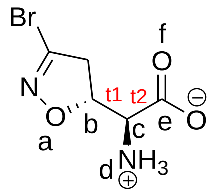

Frame	ΔE_{GM} (kcal/mol)	Torsional Angles (°)	
		**τ1**[a]	**τ2**[b]
1	0	72	-5
2	1.92	-173	5
3	4.13	-53	3

[a]τ1: abcd; [b]τ2: dcef

Table S4. ΔE_{GM} values (kcal/mol) and torsional angle values (degrees) of **1c** considering MM conformers within 5 kcal/mol from the global minimum.

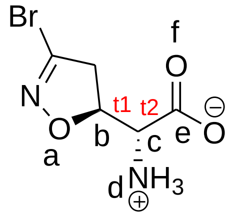

Frame	ΔE_{GM} (kcal/mol)	Torsional Angles (°)	
		**τ1**[a]	**τ2**[b]
1	0	-72	4
11	1.92	173	-4
23	4.13	53	-3

[a]τ1: abcd; [b]τ2: dcef

Table S5. ΔE_{GM} values (kcal/mol) and torsional angle values (degrees) of **1d** considering MM conformers within 5 kcal/mol from the global minimum.

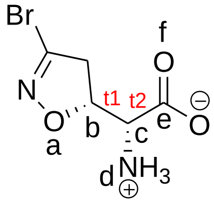

Frame	ΔE_{GM} (kcal/mol)	Torsional Angles (°)	
		**τ1**[a]	**τ2**[b]
1	0	-80	-2
13	0.41	-176	10
25	1.51	62	-2

[a]τ1: abcd; [b]τ2: dcef

Table S6. ΔE_{GM} values (kcal/mol) and torsional angle values (degrees) of **2b** considering MM conformers within 5 kcal/mol from the global minimum.

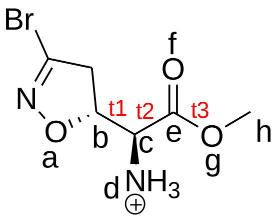

Frame	ΔE_{GM} (kcal/mol)	Torsional Angles (°)		
		**τ1**[a]	**τ2**[b]	**τ3**[c]
1	0	-62	-90	-1
2	0.26	-176	-96	-1
3	0.76	62	-95	-1
4	0.79	-63	95	1
5	1.53	-178	84	1
6	1.99	63	99	1

[a]τ1: abcd; [b]τ2: dcef; [c]τ3: fegh

Table S7. ΔE_{GM} values (kcal/mol) and torsional angle values (degrees) of **2c** considering MM conformers within 5 kcal/mol from the global minimum.

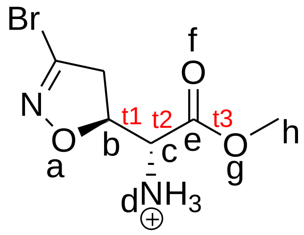

Frame	ΔE_{GM} (kcal/mol)	Torsional Angles (°)		
		**τ1**[a]	**τ2**[b]	**τ3**[c]
1	0	62	90	1
2	0.26	176	96	1
3	0.76	-62	95	1
4	0.79	63	-95	-1
5	1.53	178	-84	-1
6	1.99	-63	-99	-1

[a]τ1: abcd; [b]τ2: dcef; [c]τ3: fegh

Table S8. ΔE_{GM} values (kcal/mol) and torsional angle values (degrees) of **2d** considering MM conformers within 5 kcal/mol from the global minimum.

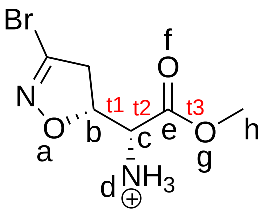

Frame	ΔE_{GM} (kcal/mol)	Torsional Angles (°)		
		**τ1**[a]	**τ2**[b]	**τ3**[c]
1	0	-179	89	1
2	0.16	-66	89	1
3	0.94	-178	-96	-1
4	1.09	-65	-97	-2
5	1.26	61	95	1
6	2.41	62	-94	-1

[a]τ1: abcd; [b]τ2: dcef; [c]τ3: fegh

Table S9. ΔE_{GM} values (kcal/mol) and torsional angle values (degrees) of **3b** considering MM conformers within 5 kcal/mol from the global minimum.

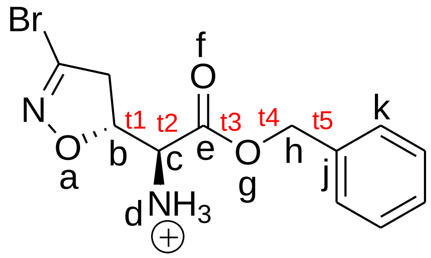

Frame	ΔE_{GM} (kcal/mol)	Torsional Angles (°)				
		**τ1**[a]	**τ2**[b]	**τ3**[c]	**τ4**[d]	**τ5**[e]
1	0	-178	82	3	171	-88
2	0.22	-62	-89	-1	-179	-90
3	0.48	-63	-86	-3	-82	106
4	0.48	-176	-96	-1	-179	90
5	0.67	-64	94	2	176	93
6	0.80	-62	-90	-1	85	-105
7	0.81	-179	100	7	96	-91
8	0.83	-64	96	4	84	75
9	0.86	-176	-92	-2	-82	-74
10	0.98	-176	-97	0	84	-106
11	1.02	62	-95	-1	-179	90
12	1.14	-174	-54	-8	-76	-71
13	1.50	-63	95	1	-86	-75
14	1.54	62	-95	0	84	75
15	1.58	62	-93	-1	-84	-75
16	1.77	63	99	2	177	-87
17	1.90	-177	78	0	-89	103
18	2.33	64	106	4	85	73
19	2.65	62	98	1	-86	105

[a]τ1: abcd; [b]τ2: dcef; [c]τ3: fegh; [d]τ4: eghj; [e]τ5: ghjk

Table S10. ΔE_{GM} values (kcal/mol) and torsional angle values (degrees) of **3c** considering MM conformers within 5 kcal/mol from the global minimum.

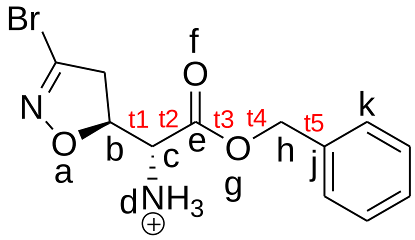

Frame	ΔE_{GM} (kcal/mol)	Torsional Angles (°)				
		τ1[a]	τ2[b]	τ3[c]	τ4[d]	τ5[e]
1	0	178	-82	-3	-171	-91
2	0.22	62	89	1	179	-90
3	0.48	63	86	3	82	74
4	0.48	176	96	1	179	-90
5	0.67	64	-94	-2	-176	-93
6	0.80	62	90	1	-85	105
7	0.81	179	-100	-7	-96	91
8	0.83	64	-96	-4	-84	-75
9	0.86	176	92	2	82	74
10	0.98	176	97	0	-84	106
11	1.02	-62	95	1	179	-90
12	1.14	174	54	8	76	71
13	1.50	63	-95	-1	86	-105
14	1.54	-62	95	0	-85	105
15	1.58	-62	93	1	84	75
16	1.77	-63	-100	-2	-178	-93
17	1.90	177	-78	0	89	76
18	2.33	-64	-106	-4	-85	-73
19	2.65	-62	-98	-1	86	-105

[a]τ1: abcd; [b]τ2: dcef; [c]τ3: fegh; [d]τ4: eghj; [e]τ5: ghjk

Table S11. ΔE_{GM} values (kcal/mol) and torsional angle values (degrees) of **3d** considering MM conformers within 5 kcal/mol from the global minimum.

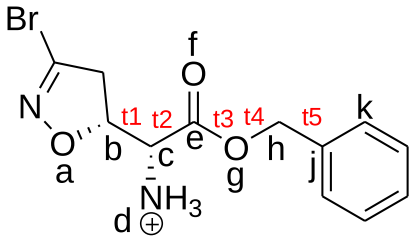

Frame	ΔE_{GM} (kcal/mol)	Torsional Angles (°)				
		**τ1**[a]	**τ2**[b]	**τ3**[c]	**τ4**[d]	**τ5**[e]
1	0	-66	-104	-5	-93	95
2	0.08	-64	-91	-4	-168	88
3	0.26	-67	71	6	77	71
4	0.37	-179	89	1	179	-90
5	0.51	-66	89	1	179	-90
6	0.80	-179	87	2	83	74
7	0.93	-179	89	1	-85	-75
8	0.98	-178	-94	-2	-177	-93
9	1.03	-66	90	1	-84	106
10	1.27	-177	-97	-5	-83	-72
11	1.65	61	95	1	179	90
12	1.76	-64	-94	-2	87	-105
13	1.78	-178	-95	-1	86	75
14	2.17	61	95	0	-84	-74
15	2.21	61	93	1	84	75
16	2.22	62	-95	-2	-178	87
17	2.90	62	-100	-3	-86	-75
18	3.16	62	-92	0	86	75

[a]τ1: abcd; [b]τ2: dcef; [c]τ3: fegh; [d]τ4: eghj; [e]τ5: ghjk

Table S12. ΔE_{GM} values (kcal/mol) and torsional angle values (degrees) of **4b** considering MM conformers within 5 kcal/mol from the global minimum.

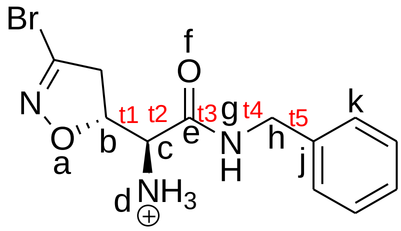

Frame	ΔE_{GM} (kcal/mol)	Torsional Angles (°)				
		**τ1**[a]	**τ2**[b]	**τ3**[c]	**τ4**[d]	**τ5**[e]
1	0	-66	-88	0	-179	-90
2	0.62	61	-90	-1	179	-90
3	0.87	178	-93	-1	179	90
4	1.02	177	87	2	165	95
5	1.65	-66	98	0	174	93
6	1.85	63	100	1	178	-87
7	2.20	-178	-45	-6	-95	-92

[a]τ1: abcd; [b]τ2: dcef; [c]τ3: fegh; [d]τ4: eghj; [e]τ5: ghjk

Table S13. ΔE_{GM} values (kcal/mol) and torsional angle values (degrees) of **4c** considering MM conformers within 5 kcal/mol from the global minimum.

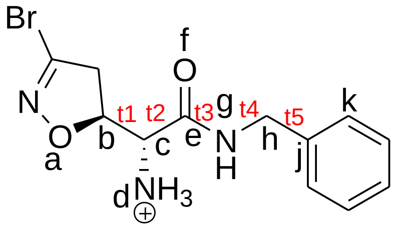

Frame	ΔE_{GM} (kcal/mol)	Torsional Angles (°)				
		**τ1**[a]	**τ2**[b]	**τ3**[c]	**τ4**[d]	**τ5**[e]
1	0	66	88	0	179	90
2	0.62	-61	90	1	-179	90
3	0.87	-178	93	1	-179	-90
4	1.02	-177	-87	-2	-165	-95
5	1.65	66	-98	0	-174	-93
6	1.85	-63	-100	-1	-178	87
7	2.20	178	45	6	95	92

[a]τ1: abcd; [b]τ2: dcef; [c]τ3: fegh; [d]τ4: eghj; [e]τ5: ghjk

Table S14. ΔE_{GM} values (kcal/mol) and torsional angle values (degrees) of **4d** considering MM conformers within 5 kcal/mol from the global minimum.

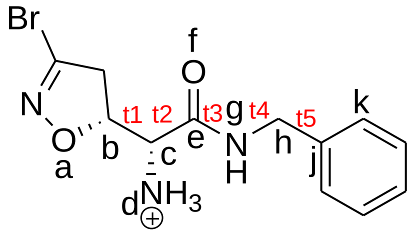

**Frame**	**ΔE_{GM} (kcal/mol)**	**Torsional Angles (°)**				
		**τ1**[a]	**τ2**[b]	**τ3**[c]	**τ4**[d]	**τ5**[e]
1	0	-175	88	1	180	90
2	0.31	-65	85	1	179	-90
3	0.47	-64	-95	-2	-151	-94
4	0.73	-66	68	5	100	-83
5	1.13	67	91	0	-179	-90
6	1.44	-174	-96	-1	-176	-92
7	2.68	66	-101	0	-176	87

[a]τ1: abcd; [b]τ2: dcef; [c]τ3: fegh; [d]τ4: eghj; [e]τ5: ghjk

Table S15. Non-bond interaction energies (kcal/mol), Cα RMSD (Å) of C153 and H180 calculated with respect to the starting position. $\chi 1$ torsion angle of C153 (degrees) and angle values of the hydrogen bond between C153 and H180 (degrees) of the **1a**/*Pf*GAPDH complexes obtained by Monte Carlo/Minimization procedure.

Complex	Binding approach	Nonbond interaction energies (kcal/mol)	RMSD (C153)	RMSD (H180)	χ_1 (C153)	Angle value of the hydrogen bond between C153 and H180	
						DHA[a]	XDA[a]
**1**[b]	BA1	-12.787	0.24	0.47	-68.15	107.27	133.35
**2**	BA1	-0.490	0.35	0.54	-63.70	102.57	112.40
**3**	BA1	-4.136	1.24	0.57	-53.26	122.06	70.29
**4**	BA1	2.226	2.66	0.57	-41.90	139.49	69.51
**5**	BA1	-6.721	1.66	0.57	-40.81	118.89	74.26
**6**	BA1	24.204	6.87	1.47	92.35	124.04	67.38
**7**[b]	BA2	-7.784	0.12	0.52	-63.39	128.94	140.45
**8**	BA2	-1.749	0.21	0.58	-73.08	108.80	130.48
**9**	BA2	1.988	0.83	0.54	-93.17	138.56	109.63
**10**	BA2	-5.467	1.08	0.52	-89.71	135.43	87.03
**11**	BA2	-7.428	1.36	0.48	-92.59	125.57	84.99
**12**	BA2	27.011	4.95	0.88	77.68	135.22	74.64
**13**	BA2	17.364	4.68	0.85	85.56	116.79	66.86
**14**	BA2	18.129	4.67	0.97	69.39	135.49	67.55

[a] D: S_γ of C153; A: N_τ of H180; H: H_γ of C153; X: Cβ of C153. [b]Selected complex.

Table S16. Non-bond interaction energies (kcal/mol), Cα RMSD (Å) of C153 and H180 calculated with respect to the starting position. χ1 torsion angle of C153 (degrees) and angle values of the hydrogen bond between C153 and H180 (degrees) of the **1b**/PfGAPDH complexes obtained by Monte Carlo/Minimization procedure.

Complex	Binding approach	Nonbond interaction energies (kcal/mol)	RMSD (C153)	RMSD (H180)	χ_1 (C153)	Angle value of the hydrogen bond between C153 and H180	
						DHA[a]	XDA[a]
**1**[b]	BA1	-15.430	0.12	0.57	-62.57	113.40	127.96
**2**	BA1	4.574	0.27	0.59	-54.73	138.45	99.78
**3**	BA1	0.424	0.94	0.56	-70.10	139.76	74.75
**4**	BA1	-3.611	0.53	0.56	-60.66	134.21	90.44
**5**	BA1	-2.849	0.39	0.59	-59.05	130.99	99.42
**6**	BA1	31.598	4.51	0.83	-35.77	89.01	109.12
**7**	BA1	18.838	4.86	0.99	-31.67	93.82	117.46
**8**	BA1	1.636	4.83	0.99	67.56	146.63	74.65
**9**[b]	BA2	-9.844	0.14	0.48	-62.82	112.25	133.74
**10**	BA2	4.410	0.33	0.72	-48.31	128.56	123.95
**11**	BA2	8.788	0.64	0.64	-66.20	161.22	92.79
**12**	BA2	-2.394	0.78	0.34	-79.24	139.30	70.09
**13**	BA2	-3.040	1.24	0.34	-90.68	127.06	68.91
**14**	BA2	35.219	5.05	1.00	80.26	133.52	63.68
**15**	BA2	12.227	4.95	0.63	78.26	132.08	65.68
**16**	BA2	9.355	4.68	0.74	71.70	136.04	69.73

[a] D: S_γ of C153; A: N_τ of H180; H: H_γ of C153; X: Cβ of C153. [b]Selected complex.

Table S17. Non-bond interaction energies (kcal/mol), Cα RMSD (Å) of C153 and H180 calculated with respect to the starting position. χ1 torsion angle of C153 (degrees) and angle values of the hydrogen bond between C153 and H180 (degrees) of the **1c**/PfGAPDH complexes obtained by Monte Carlo/Minimization procedure.

Complex	Binding approach	Nonbond interaction energies (kcal/mol)	RMSD (C153)	RMSD (H180)	χ_1 (C153)	Angle value of the hydrogen bond between C153 and H180	
						DHA[a]	XDA[a]
**1** [b]	BA1	-12.473	0.16	0.51	-61.97	112.26	133.82
**2**	BA1	25.650	0.60	0.65	-79.73	153.77	108.07
**3**	BA1	2.237	1.12	0.85	-59.28	156.62	77.83
**4**	BA1	9.163	1.06	0.78	-61.29	147.94	82.80
**5**	BA1	12.473	1.05	0.65	-51.53	135.93	68.53
**6**	BA1	28.982	4.40	1.27	88.50	116.18	64.38
**7**	BA1	13.199	4.61	1.28	86.16	132.40	64.25
**8**	BA1	8.818	4.57	1.24	83.79	135.51	66.00
**9** [b]	BA2	-11.082	0.12	0.53	-64.58	112.87	126.63
**10**	BA2	-4.452	0.11	0.54	-74.39	129.48	112.02
**11**	BA2	40.107	0.48	0.61	-65.18	114.27	104.23
**12**	BA2	5.146	0.23	0.82	-62.67	144.60	102.67
**13**	BA2	1.933	0.56	0.88	-76.39	132.48	94.82

[a] D: S_γ of C153; A: N_τ of H180; H: H_γ of C153; X: Cβ of C153. [b]Selected complex.

Table S18. Non-bond interaction energies (kcal/mol), Cα RMSD (Å) of C153 and H180 calculated with respect to the starting position. $\chi 1$ torsion angle of C153 (degrees) and angle values of the hydrogen bond between C153 and H180 (degrees) of the **1d**/PfGAPDH complexes obtained by Monte Carlo/Minimization procedure.

Complex	Binding approach	Nonbond interaction energies (kcal/mol)	RMSD (C153)	RMSD (H180)	χ_1 (C153)	Angle value of the hydrogen bond between C153 and H180	
						DHA[a]	XDA[a]
**1**[b]	BA1	-10.032	0.19	0.50	-61.41	114.39	137.94
**2**	BA1	-0.579	1.26	0.69	-69.90	130.46	76.79
**3**	BA1	-2.138	1.28	0.58	-82.20	135.07	71.07
**4**	BA1	20.777	1.90	0.56	-84.31	118.36	72.20
**5**	BA1	26.030	1.35	0.52	-63.35	116.10	79.77
**6**	BA1	21.442	4.62	1.26	85.17	99.81	69.98
**7**	BA1	-2.022	4.86	1.27	97.63	103.16	68.51
**8**	BA1	50.778	4.84	1.54	75.62	103.10	72.74
**9**[b]	BA2	-8.062	0.15	0.48	-61.26	110.53	134.50
**10**	BA2	-6.465	0.25	0.53	-61.79	111.60	117.97
**11**	BA2	-0.278	0.64	0.57	-102.38	148.38	88.39
**12**	BA2	39.124	2.48	0.48	-78.28	111.23	79.34
**13**	BA2	8.782	2.13	0.51	-45.91	87.30	106.77
**14**	BA2	39.445	5.05	1.05	80.54	135.87	64.80
**15**	BA2	14.472	5.11	1.06	79.34	134.48	70.07
**16**	BA2	12.328	4.98	0.96	81.39	134.87	69.80

[a] D: S_γ of C153; A: N_τ of H180; H: H_γ of C153; X: Cβ of C153. [b]Selected complex.

Table S19. Non-bond interaction energies (kcal/mol), Cα RMSD (Å) of C153 and H180 calculated with respect to the starting position. χ1 torsion angle of C153 (degrees) and angle values of the hydrogen bond between C153 and H180 (degrees) of the **2a**/PfGAPDH complexes obtained by Monte Carlo/Minimization procedure.

Complex	Binding approach	Nonbond interaction energies (kcal/mol)	RMSD (C153)	RMSD (H180)	χ_1 (C153)	Angle value of the hydrogen bond between C153 and H180	
						DHA[a]	XDA[a]
**1**[b]	BA1	-6.456	0.25	0.48	-67.86	112.40	130.28
**2**	BA1	5.676	0.70	0.48	-76.21	127.64	80.80
**3**	BA1	6.487	1.56	0.41	-90.21	135.60	67.45
**4**	BA1	13.313	1.10	0.40	-83.08	117.94	77.10
**5**	BA1	-3.426	0.99	0.41	-71.81	109.15	88.62
**6**	BA1	79.200	4.58	0.94	65.75	155.62	80.66
**7**	BA1	28.746	4.48	0.70	65.07	149.25	80.05
**8**	BA1	37.448	4.83	1.07	70.27	138.64	83.55
**9**[b]	BA2	-4.059	0.16	0.55	-60.84	111.51	131.29
**10**	BA2	7.461	0.29	0.60	-60.91	126.66	104.97
**11**	BA2	-0.304	0.84	0.58	-79.90	137.01	79.58
**12**	BA2	-3.161	1.06	0.53	-73.20	134.21	72.41
**13**	BA2	4.445	1.81	0.47	-92.22	129.26	70.39
**14**	BA2	25.054	4.24	0.74	78.99	127.04	70.10
**15**	BA2	12.736	5.02	1.16	84.64	141.16	70.81
**16**	BA2	17.483	4.88	1.68	58.29	162.73	81.67

[a] D: S_γ of C153; A: N_τ of H180; H: H_γ of C153; X: Cβ of C153. [b]Selected complex.

Table S20. Non-bond interaction energies (kcal/mol), Cα RMSD (Å) of C153 and H180 calculated with respect to the starting position. χ1 torsion angle of C153 (degrees) and angle values of the hydrogen bond between C153 and H180 (degrees) of the **2b**/PfGAPDH complexes obtained by Monte Carlo/Minimization procedure.

Complex	Binding approach	Nonbond interaction energies (kcal/mol)	RMSD (C153)	RMSD (H180)	χ_1 (C153)	Angle value of the hydrogen bond between C153 and H180	
						DHA[a]	XDA[a]
**1**[b]	BA1	-8.959	0.15	0.57	-61.04	112.14	130.64
**2**	BA1	-2.976	0.43	0.49	-87.28	138.17	93.15
**3**	BA1	2.077	1.12	0.42	-77.66	135.52	73.21
**4**	BA1	4.585	0.91	0.41	-71.72	113.99	88.43
**5**	BA1	-0.023	1.65	0.41	-74.59	137.36	67.07
**6**	BA1	22.756	5.29	0.95	72.96	131.14	62.80
**7**	BA1	14.245	5.06	0.94	74.52	135.71	65.98
**8**	BA1	16.293	5.33	0.99	71.71	132.16	65.38
**9**[b]	BA2	-4.535	0.14	0.47	-62.47	112.81	134.00
**10**	BA2	-3.613	0.26	0.50	-65.00	118.19	105.84
**11**	BA2	1.014	1.75	0.60	-61.81	126.48	68.89
**12**	BA2	8.990	1.31	0.57	-64.04	142.28	70.25
**13**	BA2	4.985	1.19	0.57	-63.93	143.07	74.20
**14**	BA2	13.091	4.92	1.09	83.92	115.52	68.70
**15**	BA2	10.630	5.02	1.12	93.69	118.68	70.10
**16**	BA2	33.552	4.80	1.17	75.98	139.08	69.67

[a] D: S_γ of C153; A: N_τ of H180; H: H_γ of C153; X: Cβ of C153. [b]Selected complex.

Table S21. Non-bond interaction energies (kcal/mol), Cα RMSD (Å) of C153 and H180 calculated with respect to the starting position. $\chi 1$ torsion angle of C153 (degrees) and angle values of the hydrogen bond between C153 and H180 (degrees) of the **2c**/PfGAPDH complexes obtained by Monte Carlo/Minimization procedure.

Complex	Binding approach	Nonbond interaction energies (kcal/mol)	RMSD (C153)	RMSD (H180)	χ_1 (C153)	Angle value of the hydrogen bond between C153 and H180	
						DHA[a]	XDA[a]
**1**[b]	BA1	-8.864	0.22	0.46	-67.21	105.57	133.92
**2**	BA1	3.768	0.26	0.50	-71.01	147.94	110.36
**3**	BA1	2.970	0.96	0.46	-75.69	127.68	79.40
**4**	BA1	-0.563	1.72	0.48	-62.89	126.04	69.45
**5**	BA1	26.933	1.10	0.45	-81.72	122.28	84.73
**6**	BA1	25.561	4.76	0.93	-86.11	114.79	92.87
**7**	BA1	18.299	5.08	1.12	19.62	153.27	81.06
**8**	BA1	19.095	4.77	1.12	51.30	136.12	67.21
**9**[b]	BA2	-4.923	0.21	0.53	-62.36	112.02	131.03
**10**	BA2	7.479	0.16	0.53	-68.48	118.48	124.83
**11**	BA2	17.047	0.42	0.53	-72.22	140.10	122.65
**12**	BA2	2.026	1.26	0.40	-88.57	148.12	88.42
**13**	BA2	18.105	1.85	0.39	-68.73	162.04	106.87
**14**	BA2	25.608	4.89	0.91	74.95	142.11	70.07
**15**	BA2	7.730	5.33	0.98	78.32	141.48	70.86
**16**	BA2	23.577	5.10	0.97	73.48	141.95	71.77

[a] D: S_γ of C153; A: N_τ of H180; H: H_γ of C153; X: Cβ of C153. [b]Selected complex.

Table S22. Non-bond interaction energies (kcal/mol), Cα RMSD (Å) of C153 and H180 calculated with respect to the starting position. χ1 torsion angle of C153 (degrees) and angle values of the hydrogen bond between C153 and H180 (degrees) of the **2d**/PfGAPDH complexes obtained by Monte Carlo/Minimization procedure.

**Complex**	**Binding approach**	**Nonbond interaction energies (kcal/mol)**	**RMSD (C153)**	**RMSD (H180)**	**χ_1 (C153)**	**Angle value of the hydrogen bond between C153 and H180**	
						**DHA[a]**	**XDA[a]**
**1[b]**	BA1	-4.669	0.25	0.49	-59.01	112.63	140.69
**2**	BA1	6.707	0.43	0.57	-52.56	138.01	103.76
**3**	BA1	-3.328	0.55	0.53	-68.38	146.55	87.23
**4**	BA1	-1.624	0.42	0.52	-64.00	139.37	95.65
**5**	BA1	1.640	0.73	0.48	-90.42	145.59	75.79
**6**	BA1	27.515	4.85	0.95	73.94	134.68	66.24
**7**	BA1	26.893	5.09	1.09	68.07	138.51	66.86
**8[b]**	BA2	-4.486	0.18	0.47	-61.75	110.95	136.36
**9**	BA2	31.233	0.80	0.44	-84.66	162.98	95.30
**10**	BA2	1.998	0.44	0.45	-67.82	121.66	134.07
**11**	BA2	-2.483	0.92	0.79	-73.33	157.78	101.95
**12**	BA2	6.849	1.54	1.10	-54.08	109.08	88.33
**13**	BA2	48.071	5.45	1.49	95.08	102.02	78.27
**14**	BA2	11.591	5.37	1.53	88.93	81.57	89.24
**15**	BA2	5.159	5.39	1.56	87.53	84.45	97.62

[a] D: S_γ of C153; A: N_τ of H180; H: H_γ of C153; X: Cβ of C153. [b]Selected complex.

Table S23. Non-bond interaction energies (kcal/mol), Cα RMSD (Å) of C153 and H180 calculated with respect to the starting position. χ1 torsion angle of C153 (degrees) and angle values of the hydrogen bond between C153 and H180 (degrees) of the **3a**/PfGAPDH complexes obtained by Monte Carlo/Minimization procedure.

**Complex**	**Binding approach**	**Nonbond interaction energies (kcal/mol)**	**RMSD (C153)**	**RMSD (H180)**	**χ_1 (C153)**	**Angle value of the hydrogen bond between C153 and H180**	
						**DHA[a]**	**XDA[a]**
**1**[b]	BA1	-7.548	0.29	0.50	-67.97	114.51	132.53
**2**	BA1	-1.630	0.38	0.54	-64.30	102.85	102.43
**3**	BA1	8.123	1.05	0.59	-62.61	124.13	77.21
**4**	BA1	-0.096	3.27	0.57	-57.42	132.03	67.75
**5**	BA1	3.763	2.94	0.52	-59.42	130.96	70.23
**6**	BA1	43.707	5.51	0.95	69.97	143.27	66.76
**7**[b]	BA2	-8.943	0.15	0.55	-61.13	110.89	131.72
**8**	BA2	-3.476	0.85	0.54	-95.77	127.36	91.87
**9**	BA2	-2.418	1.07	0.53	-80.34	121.48	84.06
**10**	BA2	21.830	2.04	0.40	-49.19	132.99	64.39
**11**	BA2	20.578	1.91	0.58	-25.09	159.16	86.19
**12**	BA2	65.453	5.00	1.13	62.44	124.73	59.36
**13**	BA2	57.282	5.61	1.80	59.74	142.64	91.85
**14**	BA2	-4.548	5.65	1.98	75.59	146.11	91.45

[a] D: S_γ of C153; A: N_τ of H180; H: H_γ of C153; X: Cβ of C153. [b]Selected complex.

Table S24. Non-bond interaction energies (kcal/mol), Cα RMSD (Å) of C153 and H180 calculated with respect to the starting position. χ1 torsion angle of C153 (degrees) and angle values of the hydrogen bond between C153 and H180 (degrees) of the **3b**/PfGAPDH complexes obtained by Monte Carlo/Minimization procedure.

Complex	Binding approach	Nonbond interaction energies (kcal/mol)	RMSD (C153)	RMSD (H180)	χ_1 (C153)	Angle value of the hydrogen bond between C153 and H180	
						DHA[a]	XDA[a]
**1**[b]	BA1	-8.083	0.24	0.52	-60.68	111.09	133.00
**2**	BA1	-0.558	0.63	0.47	-68.85	140.51	71.77
**3**	BA1	-0.526	0.88	0.47	-70.07	128.52	71.76
**4**	BA1	0.225	1.10	0.45	-85.75	133.74	66.31
**5**	BA1	1.173	1.30	0.41	-90.55	133.66	66.61
**6**	BA1	29.900	4.59	0.90	78.83	129.83	65.49
**7**	BA1	36.515	5.56	1.25	70.78	148.90	68.16
**8**[b]	BA2	-6.955	0.13	0.48	-62.85	113.08	132.95
**9**	BA2	0.974	0.37	0.49	-69.05	139.27	86.60
**10**	BA2	1.253	1.52	0.38	-111.84	132.92	66.31
**11**	BA2	1.591	1.90	0.38	-81.44	133.04	64.63
**12**	BA2	1.498	1.89	0.37	-72.64	135.22	66.03
**13**	BA2	62.130	4.94	0.89	26.06	136.89	61.01

[a] D: S_γ of C153; A: N_τ of H180; H: H_γ of C153; X: Cβ of C153. [b]Selected complex.

Table S25. Non-bond interaction energies (kcal/mol), Cα RMSD (Å) of C153 and H180 calculated with respect to the starting position. $\chi 1$ torsion angle of C153 (degrees) and angle values of the hydrogen bond between C153 and H180 (degrees) of the **3c**/PfGAPDH complexes obtained by Monte Carlo/Minimization procedure.

Complex	Binding approach	Nonbond interaction energies (kcal/mol)	RMSD (C153)	RMSD (H180)	χ_1 (C153)	Angle value of the hydrogen bond between C153 and H180	
						DHA[a]	XDA[a]
**1**[b]	BA1	-11.956	0.24	0.46	-66.78	103.72	136.77
**2**	BA1	0.996	1.39	0.41	-85.51	129.86	70.54
**3**	BA1	-3.224	1.51	0.41	-71.88	132.36	63.85
**4**	BA1	29.703	1.51	0.47	-56.12	135.55	74.01
**5**	BA1	38.053	1.29	0.57	-38.40	117.06	89.62
**6**	BA1	30.459	5.13	1.31	98.11	74.23	82.45
**7**	BA1	53.717	5.86	1.74	112.96	80.81	73.87
**8**	BA1	65.850	6.00	1.63	102.52	77.31	77.33
**9**[b]	BA2	-10.615	0.24	0.58	-60.98	111.57	134.81
**10**	BA2	5.234	0.89	1.05	-98.93	135.11	79.96
**11**	BA2	23.086	0.33	0.82	-80.92	127.01	106.79
**12**	BA2	10.481	0.42	0.89	-56.38	111.45	109.75
**13**	BA2	22.524	0.49	0.80	-83.60	132.03	114.42
**14**	BA2	17.192	5.31	1.17	87.69	110.85	69.75
**15**	BA2	19.364	5.63	1.28	74.89	109.19	67.83
**16**	BA2	13.948	5.90	1.39	69.12	134.83	63.66

[a] D: S_γ of C153; A: N_τ of H180; H: H_γ of C153; X: Cβ of C153. [b]Selected complex.

Table S26. Non-bond interaction energies (kcal/mol), Cα RMSD (Å) of C153 and H180 calculated with respect to the starting position. χ1 torsion angle of C153 (degrees) and angle values of the hydrogen bond between C153 and H180 (degrees) of the **3d**/PfGAPDH complexes obtained by Monte Carlo/Minimization procedure.

Complex	Binding approach	Nonbond interaction energies (kcal/mol)	RMSD (C153)	RMSD (H180)	χ_1 (C153)	Angle value of the hydrogen bond between C153 and H180	
						DHA[a]	XDA[a]
**1**[b]	BA1	-9.268	0.15	0.61	-62.84	115.65	139.87
**2**	BA1	-1.073	0.21	0.58	-75.02	123.19	96.00
**3**	BA1	-6.940	1.36	0.52	-69.37	122.49	78.16
**4**	BA1	21.722	3.31	0.63	-77.28	149.44	74.05
**5**	BA1	8.731	3.11	0.60	-72.15	142.00	69.76
**6**	BA1	39.745	5.13	0.97	74.02	131.98	90.36
**7**	BA1	3.301	4.44	0.78	57.21	124.20	113.72
**8**	BA1	38.717	5.11	1.32	64.48	128.34	139.87
**9**[b]	BA2	-7.732	0.27	0.47	-60.21	110.37	126.20
**10**	BA2	-2.542	0.44	0.43	-96.65	132.75	112.43
**11**	BA2	-4.840	1.06	0.44	-68.46	118.23	76.28
**12**	BA2	9.679	1.48	0.46	-67.88	122.01	72.56
**13**	BA2	-0.422	1.29	0.46	-64.36	130.04	74.71
**14**	BA2	30.203	7.06	1.06	-38.67	88.26	65.49
**15**	BA2	54.048	6.67	0.97	-56.33	85.89	79.58

[a] D: S_γ of C153; A: N_τ of H180; H: H_γ of C153; X: Cβ of C153. [b]Selected complex.

Table S27. Non-bond interaction energies (kcal/mol), Cα RMSD (Å) of C153 and H180 calculated with respect to the starting position. χ1 torsion angle of C153 (degrees) and angle values of the hydrogen bond between C153 and H180 (degrees) of the **4a**/PfGAPDH complexes obtained by Monte Carlo/Minimization procedure.

Complex	Binding approach	Nonbond interaction energies (kcal/mol)	RMSD (C153)	RMSD (H180)	χ_1 (C153)	Angle value of the hydrogen bond between C153 and H180	
						DHA[a]	XDA[a]
**1** [b]	BA1	-7.193	0.30	0.49	-67.75	118.81	133.97
**2**	BA1	-1.133	0.19	0.50	-63.78	110.87	123.77
**3**	BA1	7.825	1.32	0.45	-150.23	137.88	65.33
**4**	BA1	11.784	1.92	0.48	-88.28	136.90	65.28
**5**	BA1	0.382	2.19	0.42	-57.18	115.90	70.80
**6**	BA1	31.489	5.98	2.40	66.50	132.38	120.08
**7**	BA1	27.649	6.21	2.27	87.23	147.46	112.23
**8**[b]	BA2	-8.592	0.15	0.55	-61.44	111.23	131.51
**9**	BA2	-1.674	0.80	0.40	-83.05	138.26	73.16
**10**	BA2	-2.000	0.71	0.38	-69.12	123.52	77.95
**11**	BA2	8.792	1.06	0.40	-96.26	125.25	76.87
**12**	BA2	0.468	2.32	0.41	-79.71	139.48	69.07
**13**	BA2	33.031	5.01	0.78	75.50	135.61	68.07
**14**	BA2	12.445	5.07	0.93	72.85	142.57	72.40
**15**	BA2	8.165	5.23	0.73	79.85	137.32	71.91

[a] D: S_γ of C153; A: N_τ of H180; H: H_γ of C153; X: Cβ of C153. [b]Selected complex.

Table S28. Non-bond interaction energies (kcal/mol), Cα RMSD (Å) of C153 and H180 calculated with respect to the starting position. χ1 torsion angle of C153 (degrees) and angle values of the hydrogen bond between C153 and H180 (degrees) of the **4b**/PfGAPDH complexes obtained by Monte Carlo/Minimization procedure.

Complex	Binding approach	Nonbond interaction energies (kcal/mol)	RMSD (C153)	RMSD (H180)	χ_1 (C153)	Angle value of the hydrogen bond between C153 and H180	
						DHA[a]	XDA[a]
**1** [b]	BA1	-9.210	0.24	0.52	-60.716	109.973	133.558
**2**	BA1	1.238	0.32	0.51	-59.441	117.474	133.722
**3**	BA1	2.808	0.44	0.48	-71.937	130.285	86.974
**4**	BA1	-1.802	1.22	0.45	-70.121	132.247	71.701
**5**	BA1	-1.053	2.42	0.43	-68.219	126.322	74.058
**6**	BA1	54.368	5.89	1.54	42.036	129.141	54.820
**7** [b]	BA2	-8.829	0.15	0.48	-61.117	113.253	134.609
**8**	BA2	6.424	0.21	0.63	-50.689	96.497	140.972
**9**	BA2	-5.552	1.12	0.58	-70.661	121.288	75.045
**10**	BA2	14.907	1.98	0.54	-65.283	102.210	85.612
**11**	BA2	12.699	2.63	0.36	-171.189	134.414	66.610

[a] D: S_γ of C153; A: N_τ of H180; H: H_γ of C153; X: Cβ of C153. [b]Selected complex.

Table S29. Non-bond interaction energies (kcal/mol), Cα RMSD (Å) of C153 and H180 calculated with respect to the starting position. $\chi 1$ torsion angle of C153 (degrees) and angle values of the hydrogen bond between C153 and H180 (degrees) of the **4c**/PfGAPDH complexes obtained by Monte Carlo/Minimization procedure.

Complex	Binding approach	Nonbond interaction energies (kcal/mol)	RMSD (C153)	RMSD (H180)	χ_1 (C153)	Angle value of the hydrogen bond between C153 and H180	
						DHA[a]	XDA[a]
**1**[b]	BA1	-11.308	0.24	0.46	-68.298	106.515	135.050
**2**	BA1	6.045	0.73	0.47	-79.674	134.533	83.225
**3**	BA1	-7.754	0.79	0.46	-83.562	145.409	80.635
**4**	BA1	43.389	2.16	0.42	-91.101	129.520	78.582
**5**	BA1	-7.423	2.78	0.41	-64.444	122.684	75.675
**6**[b]	BA2	-10.031	0.23	0.58	-60.636	111.693	135.004
**7**	BA2	6.583	1.62	0.51	-84.190	129.563	65.748
**8**	BA2	33.100	1.16	0.55	-67.865	144.767	84.095
**9**	BA2	-2.830	3.18	0.55	-66.556	142.478	71.062
**10**	BA2	12.445	2.98	0.63	-51.666	90.754	100.242
**11**	BA2	39.554	5.15	1.12	72.671	141.922	68.779
**12**	BA2	39.481	6.02	1.30	68.903	136.415	68.834
**13**	BA2	26.735	6.25	1.29	66.143	124.229	66.560

[a] D: S_γ of C153; A: N_τ of H180; H: H_γ of C153; X: Cβ of C153. [b]Selected complex.

Table S30. Non-bond interaction energies (kcal/mol), Cα RMSD (Å) of C153 and H180 calculated with respect to the starting position. χ1 torsion angle of C153 (degrees) and angle values of the hydrogen bond between C153 and H180 (degrees) of the **4d**/PfGAPDH complexes obtained by Monte Carlo/Minimization procedure.

Complex	Binding approach	Nonbond interaction energies (kcal/mol)	RMSD (C153)	RMSD (H180)	χ_1 (C153)	Angle value of the hydrogen bond between C153 and H180	
						DHA[a]	XDA[a]
**1**[b]	BA1	-10.135	0.15	0.62	-62.88	114.49	127.58
**2**	BA1	-3.974	0.59	0.48	-75.60	128.80	85.74
**3**	BA1	1.163	1.65	0.40	-71.08	135.36	66.17
**4**	BA1	0.991	1.89	0.42	-68.27	135.55	66.47
**5**	BA1	3.749	3.41	1.04	-58.46	138.77	67.58
**6**	BA1	51.161	5.17	1.01	68.45	145.98	71.16
**7**	BA1	55.187	5.39	1.02	63.48	149.66	74.46
**8**	BA1	35.102	4.90	1.02	63.19	154.24	78.57
**9**[b]	BA2	-8.962	0.22	0.48	-60.42	110.96	137.97
**10**	BA2	14.339	1.15	0.51	-77.00	104.35	89.18
**11**	BA2	-5.076	0.55	0.53	-65.64	106.85	108.32
**12**	BA2	-0.527	1.10	0.49	-66.78	130.14	72.22
**13**	BA2	-2.602	2.15	0.71	-166.12	132.32	61.09
**14**	BA2	35.708	5.18	0.97	72.35	101.84	77.96
**15**	BA2	-3.830	5.31	1.50	60.05	133.06	82.38
**16**	BA2	26.130	5.82	0.95	69.20	111.57	83.88

[a] D: S_γ of C153; A: N_τ of H180; H: H_γ of C153; X: Cβ of C153. [b]Selected complex.

Table S31. Non-bond interaction energies (kcal/mol), Cα RMSD (Å) of C153 and H180 calculated with respect to the starting position, χ1 torsion angle of C153(degrees), and angles of the hydrogen bond between C153 and H180 (degrees) of the best docked complexes after unrestrained structure optimization.

Lig.	Binding approach	Nonbond interaction energies (kcal/mol)	RMSD (C153)	RMSD (H180)	χ_1 (C153)	Angle DHA[a]	Angle XDA[a]
**1a**	BA1	-22.590	0.52	0.74	-73.73	124.17	129.23
	BA2	-13.343	0.62	0.65	-68.64	115.89	125.47
**1b**	BA1	-17.717	0.47	0.86	-70.70	115.94	135.20
	BA2	-16.548	0.65	0.79	-74.40	116.61	131.37
**1c**	BA1	-19.845	0.51	0.66	-73.77	113.02	133.38
	BA2	-16.387	0.52	0.71	-73.81	117.95	130.53
**1d**	BA1	-18.308	0.16	0.18	-72.34	120.56	131.82
	BA2	-16.774	0.61	0.78	-75.43	119.16	131.23
**2a**	BA1	-17.205	0.37	0.81	-70.65	125.10	133.32
	BA2	-11.536	0.53	0.24	-71.44	113.63	128.58
**2b**	BA1	-17.427	0.66	0.79	-72.33	104.17	130.40
	BA2	-13.269	0.76	0.60	-76.07	118.43	127.47
**2c**	BA1	-13.921	0.56	0.95	-66.98	128.46	132.79
	BA2	-10.281	0.49	0.46	-68.15	128.04	129.50
**2d**	BA1	-14.750	0.46	0.76	-67.34	109.79	129.42
	BA2	-6.510	0.67	0.85	-69.67	117.43	133.81
**3a**	BA1	-18.374	0.48	0.76	-71.37	122.78	130.26
	BA2	-16.607	0.65	0.54	-72.08	116.39	130.52
**3b**	BA1	-19.049	0.41	0.67	-65.87	116.90	137.39
	BA2	-14.111	0.55	0.42	-74.55	120.22	128.86
**3c**	BA1	-21.182	0.53	0.96	-64.38	121.32	130.60
	BA2	-13.996	0.60	0.67	-69.63	123.43	129.39
**3d**	BA1	-24.553	0.51	1.06	-69.32	119.37	131.37
	BA2	-11.600	0.47	0.72	-70.48	124.35	131.32
**4a**	BA1	-22.191	0.48	0.83	-71.71	122.05	134.89
	BA2	-17.572	0.43	0.56	-72.41	119.55	130.34
**4b**	BA1	-23.495	0.30	0.51	-66.11	111.60	133.22
	BA2	-17.404	0.55	0.53	-71.56	121.53	124.83
**4c**	BA1	-22.441	0.40	0.92	-65.50	125.77	129.05
	BA2	-14.363	0.70	0.49	-69.16	121.15	130.51
**4d**	BA1	-24.176	0.31	0.76	-70.83	116.59	133.40
	BA2	-11.351	0.51	0.67	-71.98	120.05	131.71

[a] D: S_γ of C153; A: N_τ of H180; H: H_γ of C153; X: Cβ of C153.

Table S32. Summary of Procheck results obtained for the selected docked complexes.

Structure	Binding Approach	Residues favored regions (%)	Residues allowed regions (%)	Residues generously allowed regions (%)	Residues disallowed regions (%)	Poor rotamer (%)
**1YWG**	-	85.2	14.0	0.8	0.0	1.1
**1a**/PfGAPDH	BA1	83.1	15.7	0.7	0.5	0.9
**1a**/PfGAPDH	BA2	83.3	15.4	0.9	0.4	0.9
**1b**/PfGAPDH	BA1	83.1	15.6	0.9	0.4	0.9
**1b**/PfGAPDH	BA2	82.9	16.3	0.6	0.3	1.5
**1c**/PfGAPDH	BA1	83.0	15.7	0.9	0.4	0.9
**1c**/PfGAPDH	BA2	81.9	17.1	0.6	0.3	0.9
**1d**/PfGAPDH	BA1	83.2	15.6	0.7	0.5	0.9
**1d**/PfGAPDH	BA2	82.3	16.8	0.7	0.3	1.2
**2a**/PfGAPDH	BA1	83.2	15.3	0.9	0.5	0.9
**2a**/PfGAPDH	BA2	83.3	15.4	0.9	0.4	0.9
**2b**/PfGAPDH	BA1	83.0	15.8	0.8	0.4	0.9
**2b**/PfGAPDH	BA2	83.1	15.6	0.9	0.4	0.9
**2c**/PfGAPDH	BA1	82.9	15.9	0.7	0.5	0.9
**2c**/PfGAPDH	BA2	83.1	15.6	0.9	0.4	0.9
**2d**/PfGAPDH	BA1	83.3	15.3	0.9	0.4	0.9
**2d**/PfGAPDH	BA2	83.1	15.5	1.0	0.3	0.9
**3a**/PfGAPDH	BA1	83.4	15.2	0.9	0.4	0.9
**3a**/PfGAPDH	BA2	83.4	15.2	0.9	0.5	0.9
**3b**/PfGAPDH	BA1	83.0	15.8	0.7	0.5	0.9
**3b**/PfGAPDH	BA2	82.3	16.7	0.8	0.3	1.1
**3c**/PfGAPDH	BA1	83.3	15.3	0.9	0.4	0.9
**3c**/PfGAPDH	BA2	83.2	15.4	0.9	0.5	0.9
**3d**/PfGAPDH	BA1	83.1	15.6	0.8	0.5	0.9
**3d**/PfGAPDH	BA2	83.4	15.3	0.9	0.4	0.9
**4a**/PfGAPDH	BA1	83.4	15.3	0.8	0.5	0.9
**4a**/PfGAPDH	BA2	83.3	15.2	1.1	0.4	0.9
**4b**/PfGAPDH	BA1	83.1	15.6	0.8	0.5	0.9
**4b**/PfGAPDH	BA2	83.3	15.4	0.8	0.5	0.9
**4c**/PfGAPDH	BA1	83.6	15.0	0.9	0.5	0.9
**4c**/PfGAPDH	BA2	83.0	15.7	0.9	0.4	0.9
**4d**/PfGAPDH	BA1	83.0	15.7	0.9	0.4	0.9
**4d**/PfGAPDH	BA2	83.5	15.2	0.8	0.6	0.9

Table S33. Calculated RMSD (Å) of the ligand conformation in the docked complexes with respect to the conformers obtained by the conformational analysis. The energy difference (ΔE) from the global minimum energy conformer (GM) is also reported.

**Compound**	**Binding Approach**	**RMSD (Å)**	**ΔE_{GM} (kcal/mol)**
**1a**	BA1	0.52	0
**1a**	BA2	0.45	0
**1b**	BA1	0.36	1.92
**1b**	BA2	0.39	1.92
**1c**	BA1	0.48	1.92
**1c**	BA2	0.49	1.92
**1d**	BA1	0.19	1.51
**1d**	BA2	0.68	0
**2a**	BA1	0.16	1.09
**2a**	BA2	0.30	1.09
**2b**	BA1	0.18	1.53
**2b**	BA2	0.70	0.26
**2c**	BA1	0.49	1.53
**2c**	BA2	0.28	1.53
**2d**	BA1	0.47	1.25
**2d**	BA2	0.30	1.09
**3a**	BA1	0.28	0.08
**3a**	BA2	0.39	0.51
**3b**	BA1	0.48	1.77
**3b**	BA2	0.68	0.86
**3c**	BA1	0.29	0.81
**3c**	BA2	0.27	0.81
**3d**	BA1	0.23	0.26
**3d**	BA2	0.22	0
**4a**	BA1	0.28	0.47
**4a**	BA2	0.44	0.31
**4b**	BA1	0.49	1.85
**4b**	BA2	0.19	2.20
**4c**	BA1	1.01	15.9
**4c**	BA2	0.93	16.6
**4d**	BA1	0.28	0.73
**4d**	BA2	0.26	0.47

Table S34. Solvent accessible surface area (SASA) of bromine for the selected docked complexes.

**Structure**	**Binding Approach**	**SASA (Å^2)**
**1a**/PfGAPDH	BA1	9.420
**1a**/PfGAPDH	BA2	20.999
**1b**/PfGAPDH	BA1	14.658
**1b**/PfGAPDH	BA2	0
**1c**/PfGAPDH	BA1	5.332
**1c**/PfGAPDH	BA2	7.356
**1d**/PfGAPDH	BA1	0
**1d**/PfGAPDH	BA2	8.651
**2a**/PfGAPDH	BA1	5.739
**2a**/PfGAPDH	BA2	26.358
**2b**/PfGAPDH	BA1	13.929
**2b**/PfGAPDH	BA2	23.373
**2c**/PfGAPDH	BA1	0
**2c**/PfGAPDH	BA2	18.548
**2d**/PfGAPDH	BA1	5.224
**2d**/PfGAPDH	BA2	16.811
**3a**/PfGAPDH	BA1	5.559
**3a**/PfGAPDH	BA2	26.382
**3b**/PfGAPDH	BA1	0
**3b**/PfGAPDH	BA2	20.629
**3c**/PfGAPDH	BA1	0
**3c**/PfGAPDH	BA2	1.808
**3d**/PfGAPDH	BA1	3.003
**3d**/PfGAPDH	BA2	6.644
**4a**/PfGAPDH	BA1	0
**4a**/PfGAPDH	BA2	25.938
**4b**/PfGAPDH	BA1	0
**4b**/PfGAPDH	BA2	11.372
**4c**/PfGAPDH	BA1	0
**4c**/PfGAPDH	BA2	8.143
**4d**/PfGAPDH	BA1	0
**4d**/PfGAPDH	BA2	7.921

Table S35. Ligand-residue interactions of the *Pf*GAPDH/**1a**, *Pf*GAPDH/**1b**, *Pf*GAPDH/**1c**, and *Pf*GAPDH/**1d** docked complexes**.**

Cmpd	Binding approach	Ligand Group/Function	*Pf*GAPDH interacting residue	Substructure	Interaction type
**1a**	BA1	Carboxylic	R237	S11	Ionic
			T183	S-loop	Hydrogen bond
			N185	S-loop	Hydrogen bond
		Amine	D198	S-loop	Ionic
			S213	Active-site segment	Hydrogen bond
		4,5-dihydroisoxazole ring	A212	Active-site segment	Alkyl
			A235	S11	Alkyl
**1a**	BA2	Carboxylic	N185	S-loop	Hydrogen bond
			NAD (H_2O)	-	Hydrogen bond
		Amine	NAD (H_2O)	S9-H4-loop	Hydrogen bond
		4,5-dihydroisoxazole ring	C153	S9-H4-loop	Alkyl
			H180	S10	π-alkyl
			NAD	-	π-alkyl
**1b**	BA1	Carboxylic	T214	Active-site segment	Hydrogen bond
			R237	S11	Ionic
		Amine	H180	S10	Cation-π
		4,5-dihydroisoxazole ring	NAD	-	π-alkyl
**1b**	BA2	Carboxylic	N185	S-loop	Hydrogen bond
			T214 (H_2O)	Active-site segment	Hydrogen bond
			R237	S11	Ionic
		Amine	T183	S-loop	Hydrogen bond
			NAD (H_2O)	-	Hydrogen bond
		4,5-dihydroisoxazole ring	C153	S9-H4-loop	Alkyl
			H180	S10	π-alkyl
**1c**	BA1	Carboxylic	R237	S11	Ionic
			T214	Active-site segment	Hydrogen bond
		Amine	T183	S-loop	Hydrogen bond
			H180	S10	Cation-π
		4,5-dihydroisoxazole ring	NAD	-	π-alkyl
**1c**	BA2	Carboxylic	R237	S11	Ionic
			N185	S-loop	Hydrogen bond
			NAD (H_2O)	-	Hydrogen bond
		Amine	T183	S-loop	Hydrogen bond
			NAD (H_2O)	-	Hydrogen bond
		4,5-dihydroisoxazole ring	C153	S9-H4-loop	Alkyl
			H180	S10	π-alkyl
**1d**	BA1	Carboxylic	R237	S11	Ionic
			N185	S-loop	Hydrogen bond
		Amine	T183	S-loop	Hydrogen bond
		4,5-dihydroisoxazole ring	NAD	-	π-alkyl
**1d**	BA2	Carboxylic	T214	Active-site segment	Hydrogen bond
			R237	S11	Ionic
		Amine	NAD (H_2O)	-	Hydrogen bond
		4,5-dihydroisoxazole ring	C153	S9-H4-loop	Alkyl
			H180	S10	π-alkyl

Table S36. Ligand-residue interactions of the *Pf*GAPDH/**2a**, *Pf*GAPDH/**2b**, *Pf*GAPDH/**2c**, and *Pf*GAPDH/**2d** docked complexes**.**

Cmpd	Binding approach	Ligand Group/Function	*Pf*GAPDH interacting residue	Substructure	Interaction type
**2a**	BA1	Ester	T214	Active-site segment	CHO bond
		Amine	T214	Active-site segment	Hydrogen bond
			S152	S9-H4-loop	Hydrogen bond
		4,5-dihydroisoxazole ring	C153	S9-H4-loop	Alkyl
			H180	S10	π-alkyl
**2a**	BA2	Ester	N185	S-loop	Hydrogen bond
		Amine	T183	S-loop	Hydrogen bond
			NAD	-	Ionic
		4,5-dihydroisoxazole ring	C153	S9-H4-loop	Alkyl
			H180	S10	π-alkyl
**2b**	BA1	Amine	T154	S9-H4-loop	Hydrogen bond
			T214	Active-site segment	Hydrogen bond
		4,5-dihydroisoxazole ring	C153	S9-H4-loop	Alkyl
			H180	S10	π-alkyl
**2b**	BA2	Ester	R237	S11	Hydrogen bond
		Amine	T183	S-loop	Hydrogen bond
			NAD (H_2O)	-	Hydrogen bond
		4,5-dihydroisoxazole ring	C153	S9-H4-loop	Alkyl
			H180	S10	π-alkyl
			NAD (H_2O)	-	Hydrogen bond
**2c**	BA1	Ester	T183	S-loop	CHO bond
			H180	S10	π-alkyl
		Amine	T154	S9-H4-loop	Hydrogen bond
			H180	S10	Cation-π
		4,5-dihydroisoxazole ring	C153	S9-H4-loop	Alkyl
			H180	S10	π-alkyl
			NAD (H_2O)	-	Hydrogen bond
**2c**	BA2	Ester	R237	S11	Hydrogen bond
			N185	S-loop	Hydrogen bond
		Amine	NAD (H_2O)	-	Hydrogen bond
		4,5-dihydroisoxazole ring	C153	S9-H4-loop	Alkyl
			H180	S10	π-alkyl
			NAD	-	π-alkyl
**2d**	BA1	Ester	S213	Active-site segment	CHO bond
		Amine	T154	S9-H4-loop	Hydrogen bond
			T214	Active-site segment	Hydrogen bond
		4,5-dihydroisoxazole ring	C153	S9-H4-loop	Alkyl
			H180	S10	π-alkyl
			NAD	-	π-alkyl
**2d**	BA2	Ester	N185	S-loop	Hydrogen bond
		Ammine	NAD (H_2O)	-	Hydrogen bond
		4,5-dihydroisoxazole ring	C153	S9-H4-loop	Alkyl
			H180	S10	π-alkyl
			NAD	-	π-alkyl

Table S37. Ligand-residue interactions of the *Pf*GAPDH/**3a**, *Pf*GAPDH/**3b**, *Pf*GAPDH/**3c**, and *Pf*GAPDH/**3d** docked complexes**.**

**Cmpd**	**Binding approach**	**Ligand Group/Function**	***Pf*GAPDH interacting residue**	**Substructure**	**Interaction type**
**3a**	BA1	Amine	T214	Active-site segment	Hydrogen bond
			S152	S9-H4-loop	CHO bond
		4,5-dihydroisoxazole ring	C153	S9-H4-loop	Alkyl
			H180	S10	π-alkyl
**3a**	BA2	Amine	NAD (H_2O)	-	Hydrogen bond
			C153	S9-H4-loop	Alkyl
		4,5-dihydroisoxazole ring	H180	S10	π-alkyl
			NAD	-	π-alkyl
**3b**	BA1	Amine	T154	S9-H4-loop	Hydrogen bond
			H180	S10	Cation-π
			C153	S9-H4-loop	Alkyl
		4,5-dihydroisoxazole ring	H180	S10	π-alkyl
			NAD (H_2O)	-	Hydrogen bond
**3b**	BA2	Amine	T183	S-loop	Hydrogen bond
			S182	S-loop	Hydrogen bond
			NAD (H_2O)	-	Hydrogen bond
			E320 (H_2O)	S16-H8-loop	Hydrogen bond
			C153	S9-H4-loop	Alkyl
		4,5-dihydroisoxazole ring	H180	S10	π-alkyl
			NAD (H_2O)	-	Hydrogen bond
**3c**	BA1	Amine	T154	S9-H4-loop	Hydrogen bond
			H180	S10	Cation-π
		Phenyl	N185	S-loop	π- π
		4,5-dihydroisoxazole ring	C153	S9-H4-loop	Alkyl
			H180	S10	π-alkyl
**3c**	BA2	Ester	N185	S-loop	Hydrogen bond
			R237	S11	Hydrogen bond
		Amine	NAD	-	Cation-π
		Phenyl	A235	S11	π-alkyl
			C153	S9-H4-loop	Alkyl
		4,5-dihydroisoxazole ring	H180	S10	π-alkyl
			NAD	-	π-alkyl
**3d**	BA1	Ester	S213	Active-site segment	CHO bond
			T214	Active-site segment	CHO bond
		Amine	H180	S10	Cation-π
		4,5-dihydroisoxazole ring	C153	S9-H4-loop	Alkyl
			H180	S10	π-alkyl
**3d**	BA2	Ester	R237	S11	Hydrogen bond
			N185	S-loop	Hydrogen bond
		Amine	NAD	-	Ionic
		Phenyl	A235	S11	π-alkyl
			C153	S9-H4-loop	Alkyl
		4,5-dihydroisoxazole ring	H180	S10	π-alkyl
			NAD	-	π-alkyl

Table S38. Ligand-residue interactions of the *Pf*GAPDH/**4a**, *Pf*GAPDH/**4b**, *Pf*GAPDH/**4c**, and *Pf*GAPDH/**4d** docked complexes.

Cmpd	Binding approach	Ligand Group	*Pf*GAPDH interacting residue	Substructure	Interaction type
**4a**	BA1	Amine	T214	Active-site segment	Hydrogen bond
			S152	S9-H4-loop	CHO bond
		4,5-dihydroisoxazole ring	C153	S9-H4-loop	Hydrogen bond
			H180	S10	π-alkyl
**4a**	BA2	Amide	R237	S11	Hydrogen bond
			NAD	-	Hydrogen bond
			NAD (H_20)	-	Hydrogen bond
		Amine	S182	S-loop	Hydrogen bond
			T183	S-loop	Hydrogen bond
		Phenyl	A123	S7-S8-loop	π-alkyl
		4,5-dihydroisoxazole ring	H180	S10	π-alkyl
			C153	S9-H4-loop	Alkyl
**4b**	BA1	Amide	N185	S-loop	Hydrogen bond
			H180	S10	Hydrogen bond
		Amine	A181	S10	Hydrogen bond
			T183	S-loop	Hydrogen bond
			Q186	S-loop	Cation-π
		Phenyl	A212	Active-site segment	π-alkyl
			C153	S9-H4-loop	Alkyl
		4,5-dihydroisoxazole ring	H180	S10	π-alkyl
			NAD	-	π-alkyl
**4b**	BA2	Amide	N185	S-loop	Hydrogen bond
			NAD (H_2O)	-	Hydrogen bond
		Amine	S182	S-loop	Hydrogen bond
			T183	S-loop	Hydrogen bond
		Phenyl	P124	S7-S8-loop	π-alkyl
			A216	Active-site segment	π-alkyl
		4,5-dihydroisoxazole ring	H180	S10	π-alkyl
**4c**	BA1	Amine	T154	S9-H4-loop	Hydrogen bond
			H180	S10	Cation-π
		4,5-dihydroisoxazole ring	C153	S9-H4-loop	Alkyl
			H180	S10	π-alkyl
**4c**	BA2	Amide	N185	S-loop	Hydrogen bond
		Amine	NAD	-	Hydrogen bond
		Phenyl	A235	S11	π-alkyl
			R237	S11	Cation-π
			C153	S9-H4-loop	Alkyl
		4,5-dihydroisoxazole ring	H180	S10	π-alkyl
			NAD	-	π-alkyl
**4d**	BA1	Amide	T214	Active-site segment	Hydrogen bond
		Amine	H180	S10	Cation-π
		Phenyl	P124	S7-S8-loop	π-alkyl
		4,5-dihydroisoxazole ring	C153	S9-H4-loop	Alkyl
			H180	S10	π-alkyl
**4d**	BA2	Amide	NAD	-	Hydrogen bond
		Amine	NAD	-	Ionic
			N185	S-loop	Hydrogen bond
		Phenyl	P124	S7-S8-loop	π-alkyl
			A216	Active-site segment	π-alkyl
		4,5-dihydroisoxazole ring	NAD	-	π-alkyl

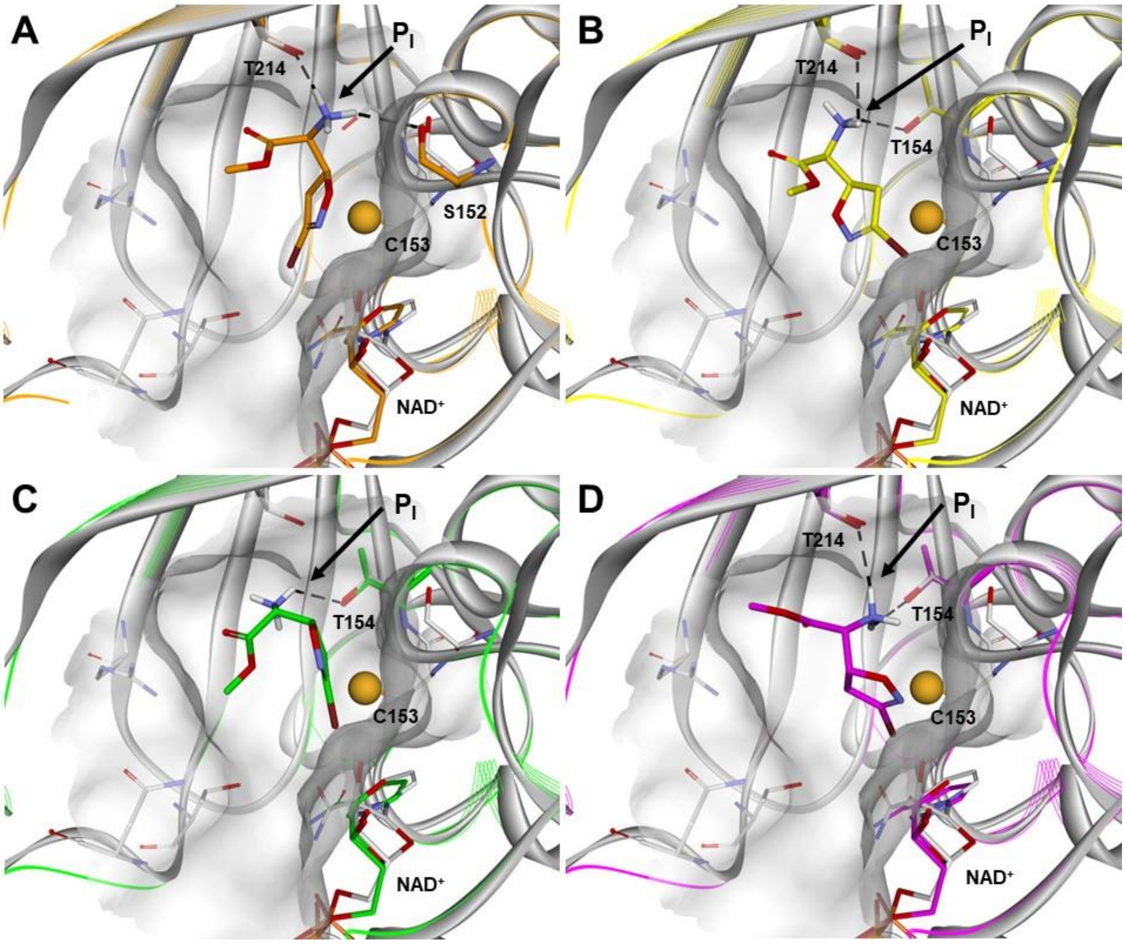

Figure S1. Docked complexes of **2a** (orange), **2b** (yellow), **2c** (green) and **2d** (magenta) bound to P_I site of *Pf*GAPDH. The starting protein (gray) is displayed as Connolly surface and solid ribbons. The key interaction residues are displayed and labelled. The ligands, NAD^+ and C153, as well as the residues involved in the interactions with ligands (stick) are colored by atom type (N, blue; O, red; S, yellow; Br, brown). The sulfur atom of C153 is displayed in CPK (scaled by 50%). Hydrogen atoms are omitted for clarity, except those involved in hydrogen bond interactions (black dashed lines).

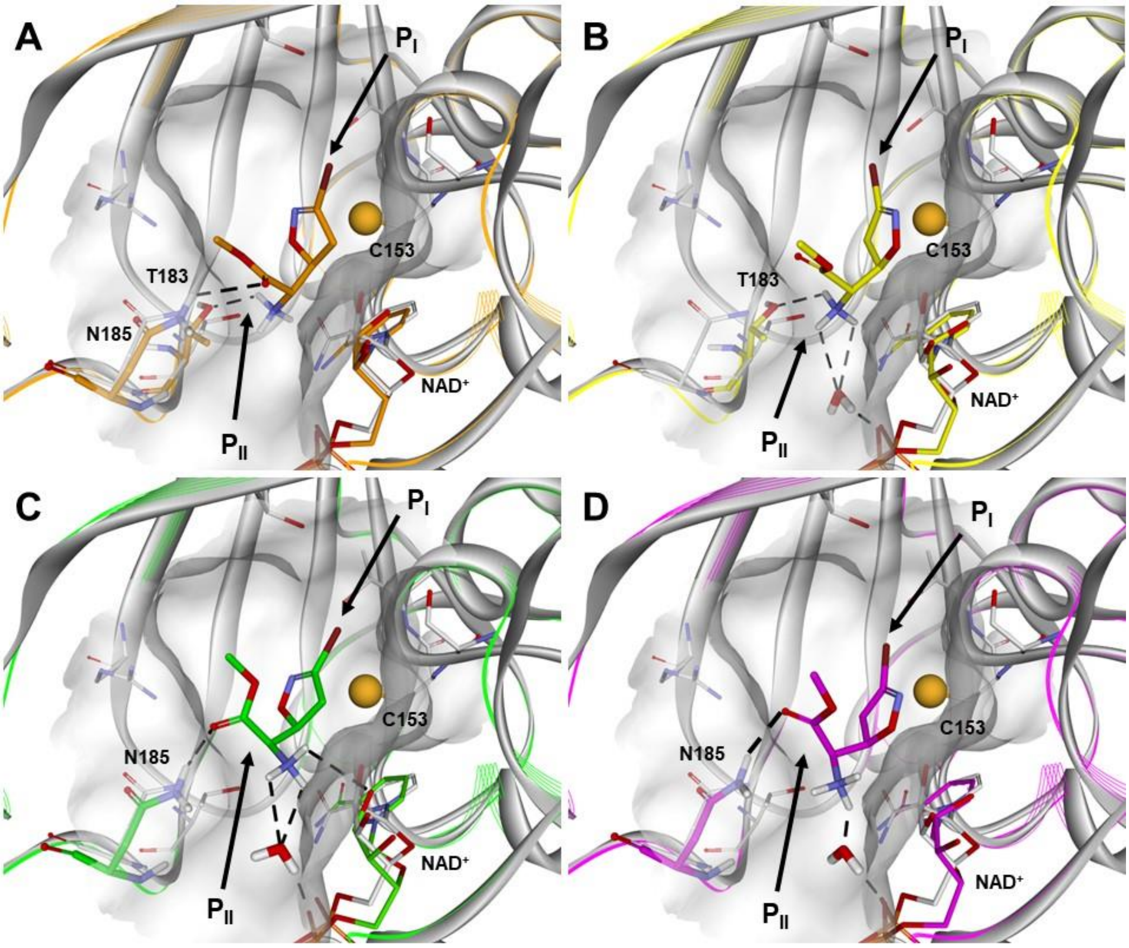

Figure S2. Docked complexes of **2a** (orange), **2b** (yellow), **2c** (green) and **2d** (magenta) bound to P_{II} site of *Pf*GAPDH. The starting protein (gray) is displayed as Connolly surface and solid ribbons. The key interaction residues are displayed and labelled. The ligands, NAD^+ and C153, as well as the residues involved in the interactions with ligands (stick) are colored by atom type (N, blue; O, red; S, yellow; Br, brown). The sulfur atom of C153 is displayed in CPK (scaled by 50%). Hydrogen atoms are omitted for clarity, except those involved in hydrogen bond interactions (black dashed lines).

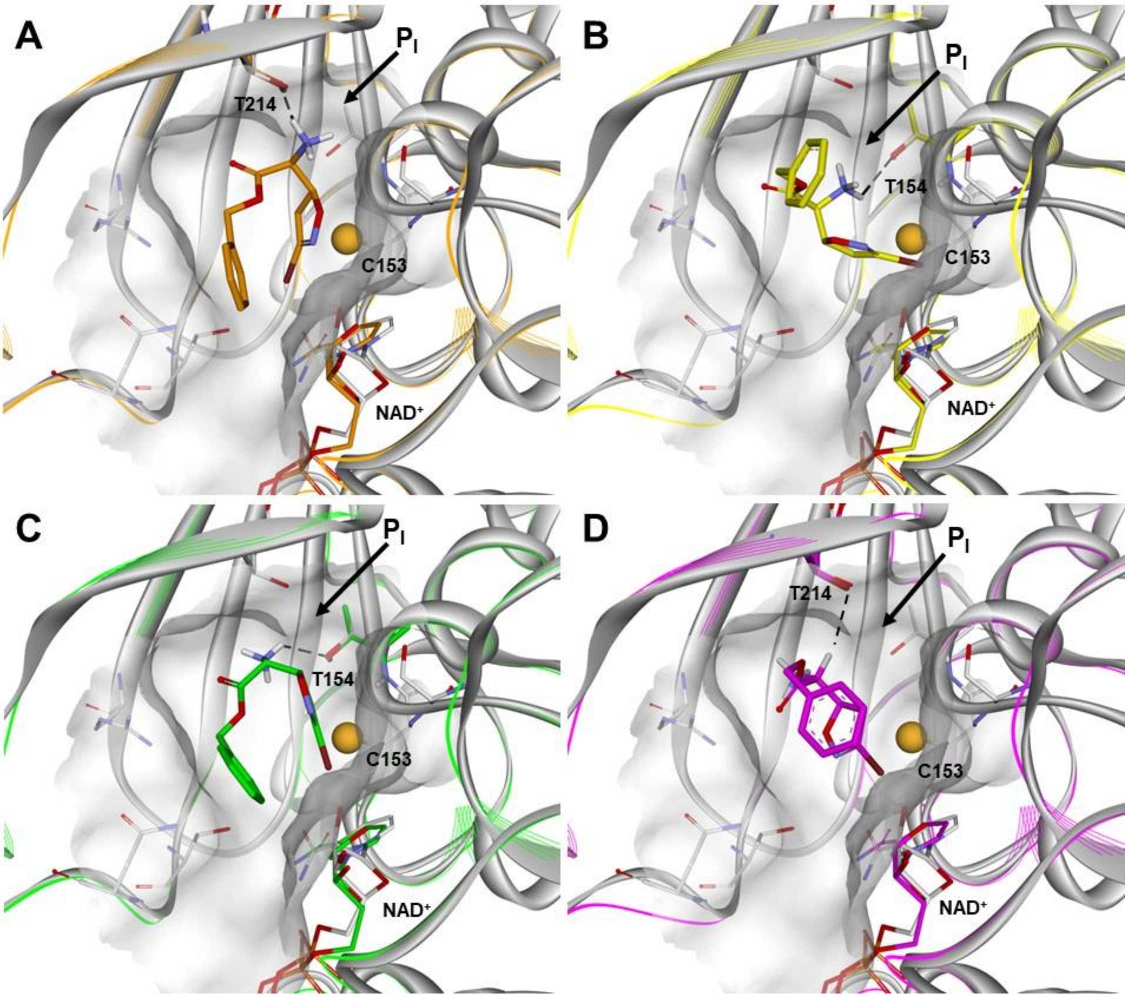

Figure S3. Docked complexes of **3a** (orange), **3b** (yellow), **3c** (green) and **3d** (magenta) bound to P_I site of *Pf*GAPDH. The starting protein (gray) is displayed as Connolly surface and solid ribbons. The key interaction residues are displayed and labelled. The ligands, NAD^+ and C153, as well as the residues involved in the interactions with ligands (stick) are colored by atom type (N, blue; O, red; S, yellow; Br, brown). The sulfur atom of C153 is displayed in CPK (scaled by 50%). Hydrogen atoms are omitted for clarity, except those involved in hydrogen bond interactions (black dashed lines).

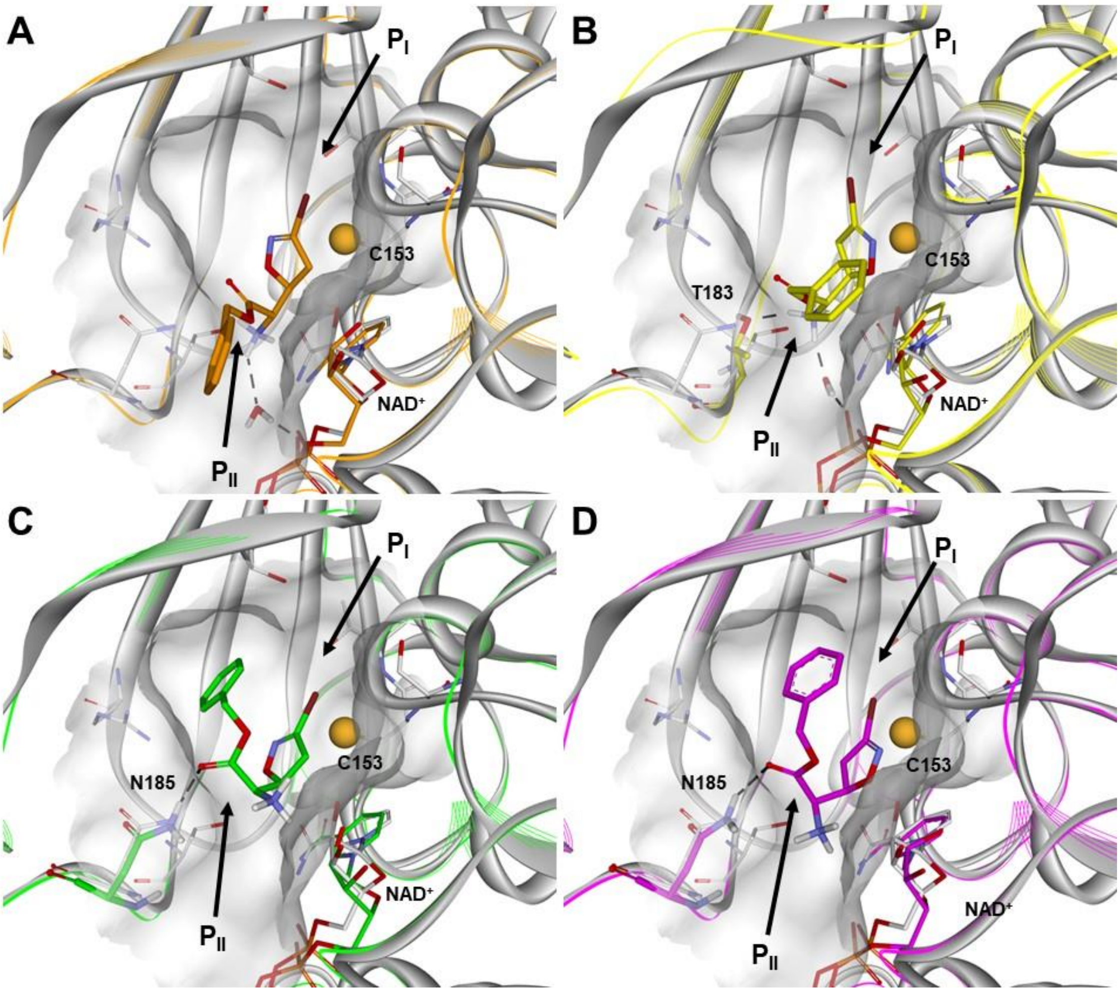

Figure S4. Docked complexes of **3a** (orange), **3b** (yellow), **3c** (green) and **3d** (magenta) bound to P$_{II}$ site of *Pf*GAPDH. The starting protein (gray) is displayed as Connolly surface and solid ribbons. The key interaction residues are displayed and labelled. The ligands, NAD^{+} and C153, as well as the residues involved in the interactions with ligands (stick) are colored by atom type (N, blue; O, red; S, yellow; Br, brown). The sulfur atom of C153 is displayed in CPK (scaled by 50%). Hydrogen atoms are omitted for clarity, except those involved in hydrogen bond interactions (black dashed lines).

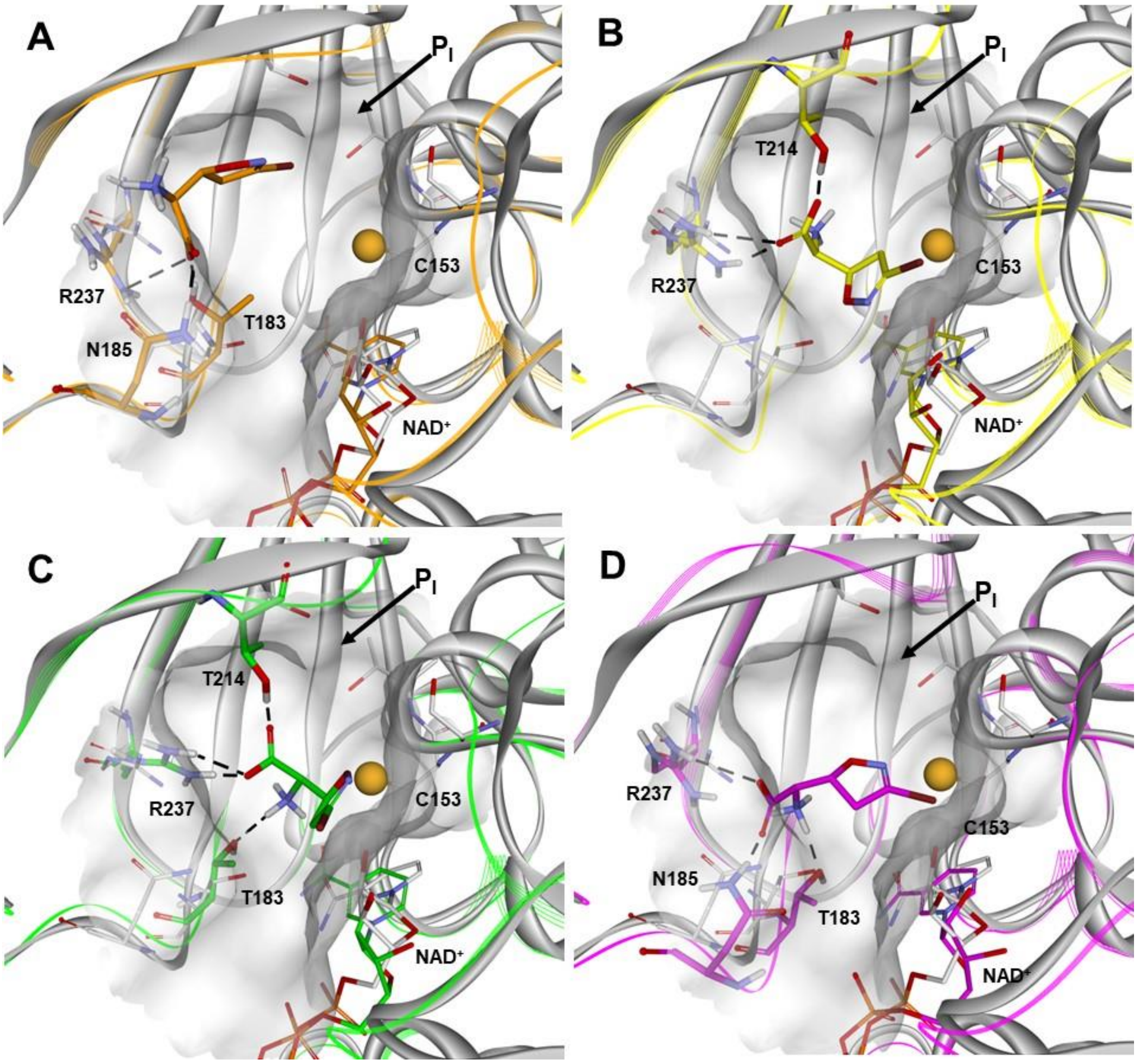

Figure S5. Docked complexes of **1a** (orange), **1b** (yellow), **1c** (green) and **1d** (magenta) bound to P$_I$ site of *Pf*GAPDH. The starting protein (gray) is displayed as Connolly surface and solid ribbons. The key interaction residues are displayed and labelled. The ligands, NAD^+ and C153, as well as the residues involved in the interactions with ligands (stick) are colored by atom type (N, blue; O, red; S, yellow; Br, brown). The sulfur atom of C153 is displayed in CPK (scaled by 50%). Hydrogen atoms are omitted for clarity, except those involved in hydrogen bond interactions (black dashed lines).

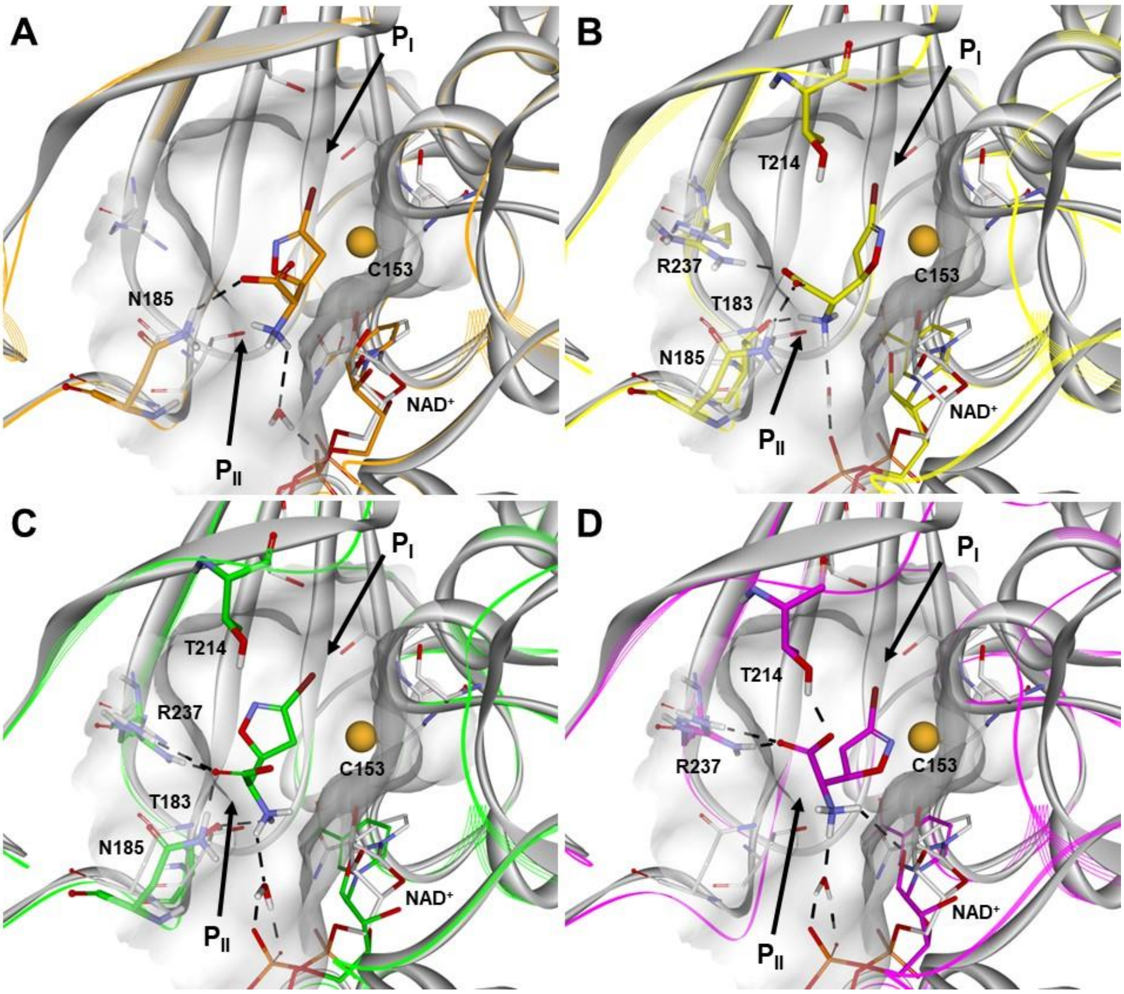

Figure S6. Docked complexes of **1a** (orange), **1b** (yellow), **1c** (green) and **1d** (magenta) bound to P_{II} site of *Pf*GAPDH. The starting protein (gray) is displayed as Connolly surface and solid ribbons. The key interaction residues are displayed and labelled. The ligands, NAD^+ and C153, as well as the residues involved in the interactions with ligands (stick) are colored by atom type (N, blue; O, red; S, yellow; Br, brown). The sulfur atom of C153 is displayed in CPK (scaled by 50%). Hydrogen atoms are omitted for clarity, except those involved in hydrogen bond interactions (black dashed lines).

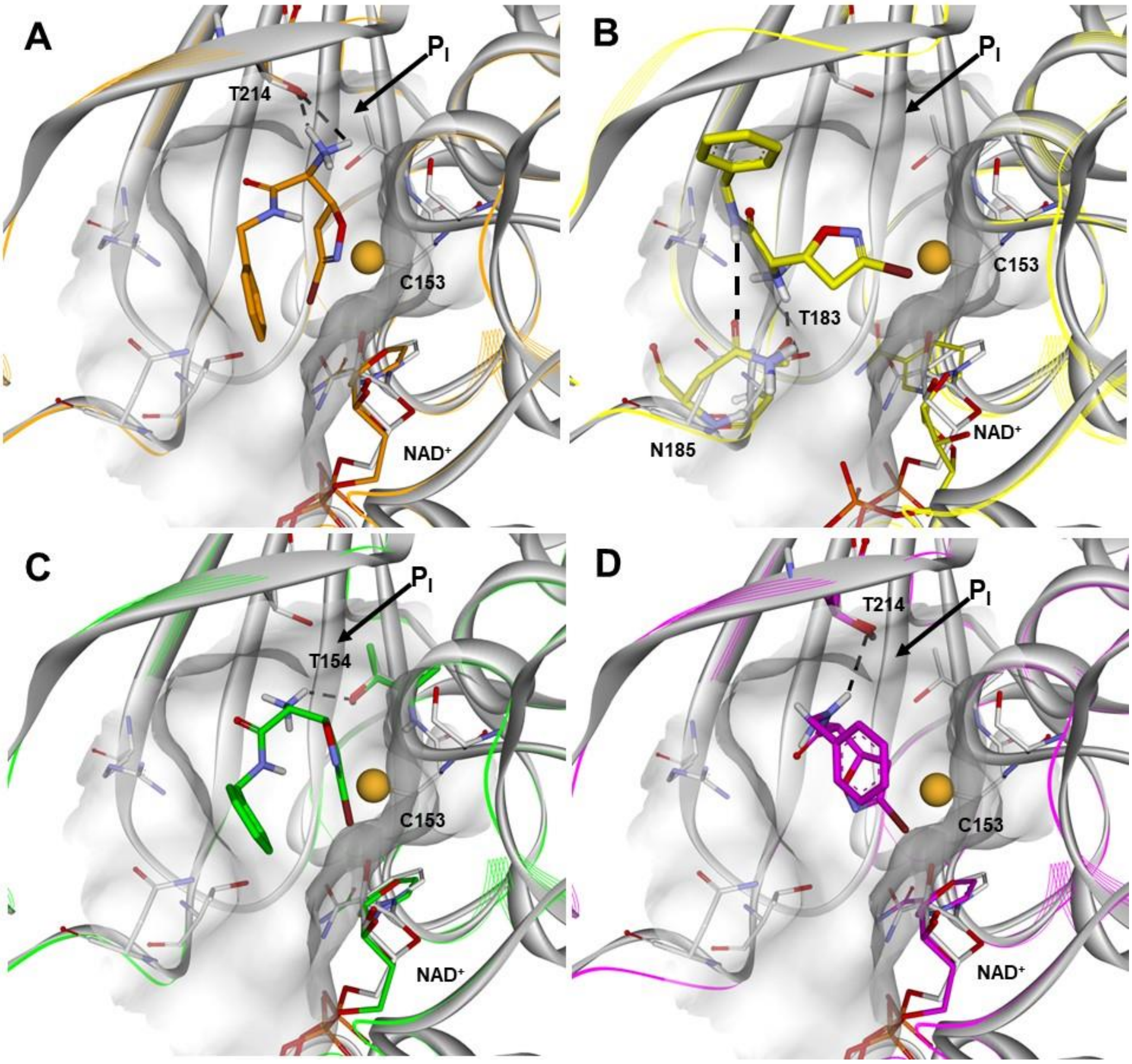

Figure S7. Docked complexes of **4a** (orange), **4b** (yellow), **4c** (green) and **4d** (magenta) bound to P_I site of *Pf*GAPDH. The starting protein (gray) is displayed as Connolly surface and solid ribbons. The key interaction residues are displayed and labelled. The ligands, NAD^+ and C153, as well as the residues involved in the interactions with ligands (stick) are colored by atom type (N, blue; O, red; S, yellow; Br, brown). The sulfur atom of C153 is displayed in CPK (scaled by 50%). Hydrogen atoms are omitted for clarity, except those involved in hydrogen bond interactions (black dashed lines).

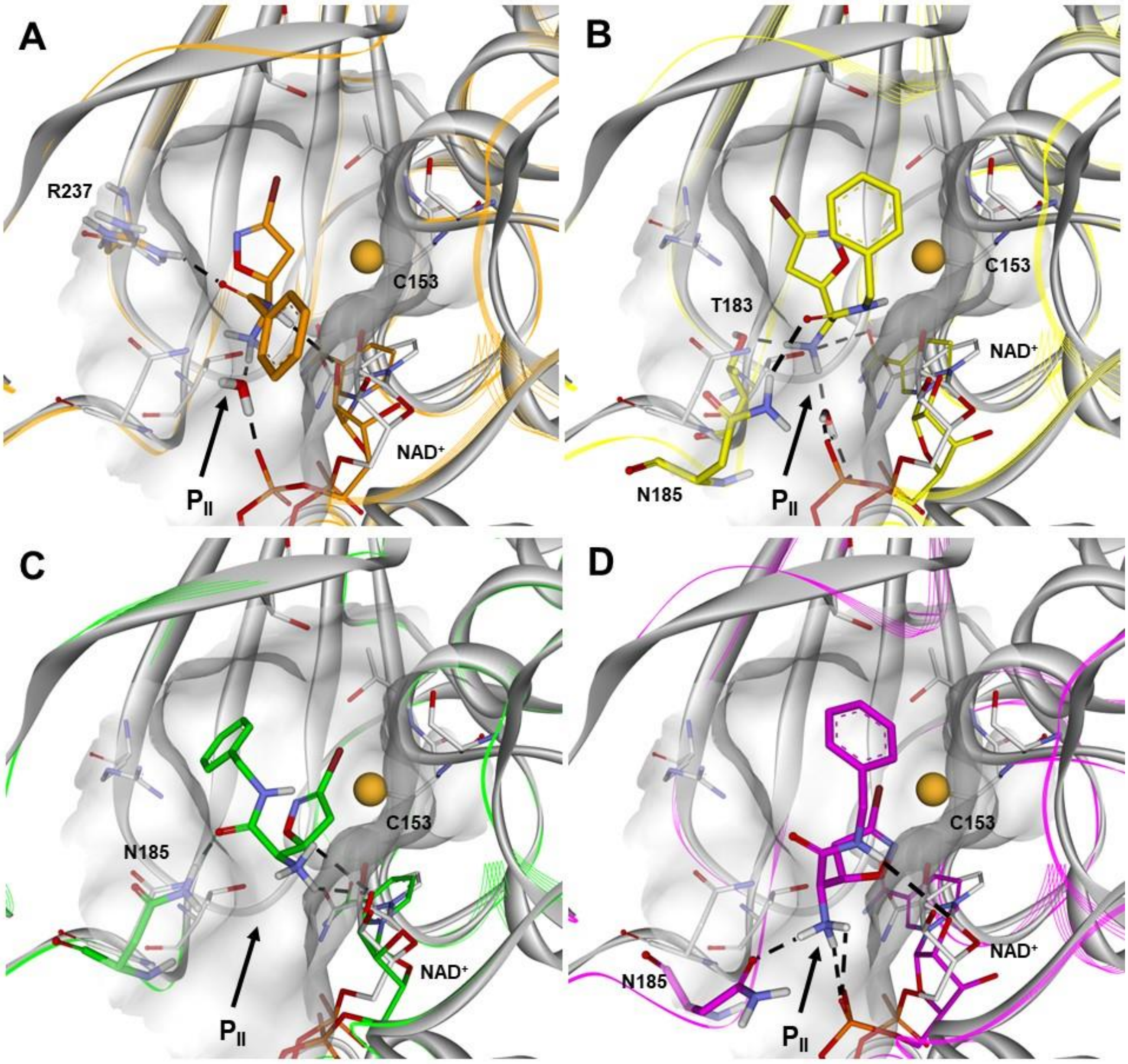

Figure S8. Docked complexes of **4a** (orange), **4b** (yellow), **4c** (green) and **4d** (magenta) bound to P_{II} site of *Pf*GAPDH. The starting protein (gray) is displayed as Connolly surface and solid ribbons. The key interaction residues are displayed and labelled. The ligands, NAD^+ and C153, as well as the residues involved in the interactions with ligands (stick) are colored by atom type (N, blue; O, red; S, yellow; Br, brown). The sulfur atom of C153 is displayed in CPK (scaled by 50%). Hydrogen atoms are omitted for clarity, except those involved in hydrogen bond interactions (black dashed lines).

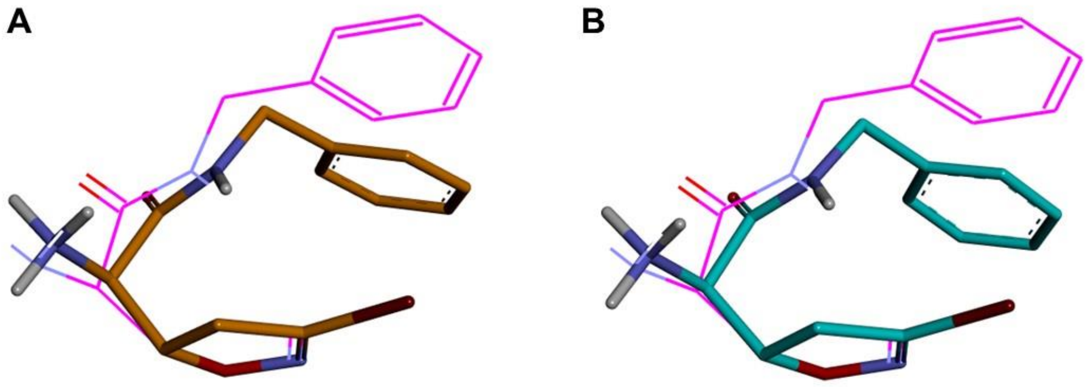

Figure S9. Superimposition of the two **4c** conformations obtained from docking studies (orange BA1; cyan BA2) on the corresponding local energy minimum conformer obtained from conformational analysis (magenta and lines). The structures are superimposed by of 4,5-dihydroisoxazole ring heavy atoms and colored by atom type (N, blue; O, red; Br, brown).

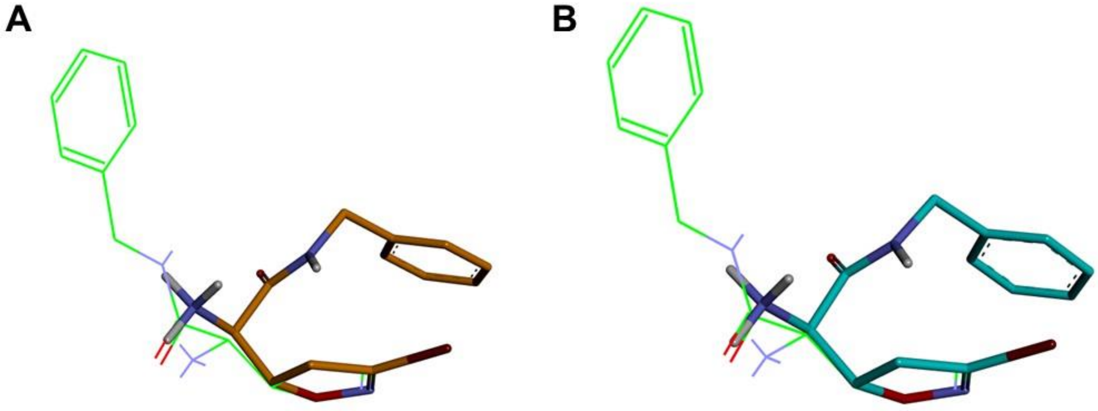

Figure S10. Superimposition of the two **4c** conformations obtained from docking studies (orange BA1; cyan BA2) on the global energy minimum conformer obtained from the conformational analysis (green and lines). The structures are superimposed by the 4,5-dihydroisoxazole ring heavy atoms and colored by atom type (N, blue; O, red; Br, brown).

^{1}H and $^{13}C\{^{1}H\}$ NMR Spectra

(*S*)-2-amino-2-((*R*)-3-bromo-4,5-dihydroisoxazol-5-yl)acetic acid (**1b**):

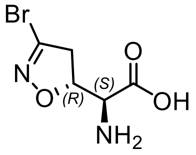

(5*R*,α*S*)-**1b**

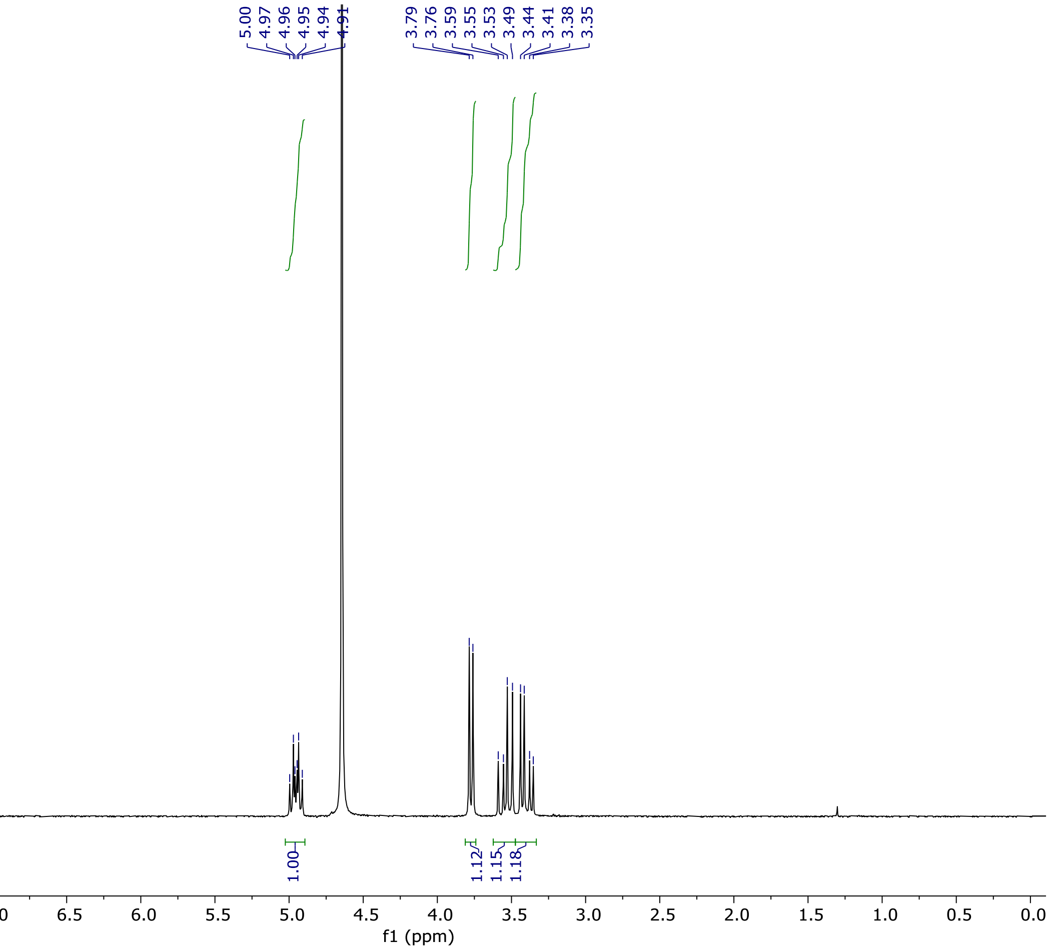

(*S*)-2-amino-2-((*R*)-3-bromo-4,5-dihydroisoxazol-5-yl)acetic acid (**1b**):

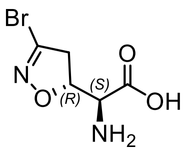

(5*R*,α*S*)-**1b**

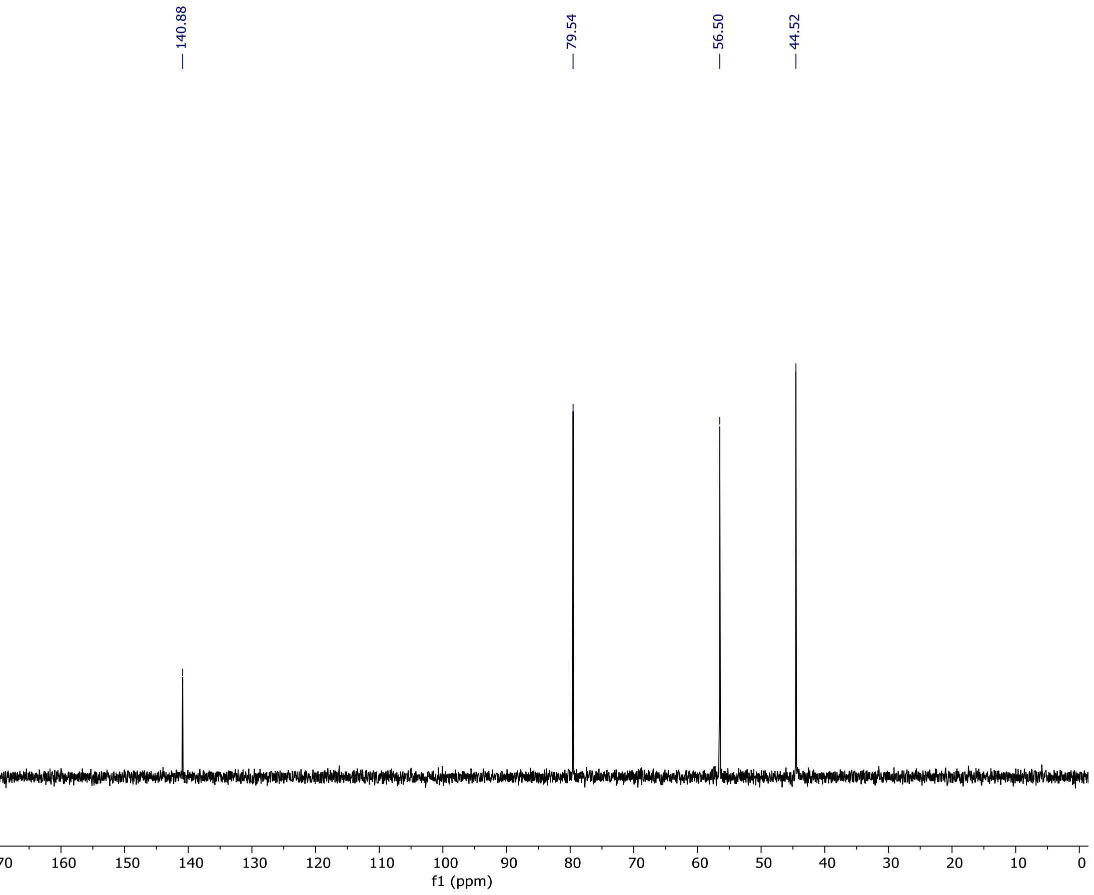

(*R*)-2-amino-2-((*R*)-3-bromo-4,5-dihydroisoxazol-5-yl)acetic acid (**1d**):

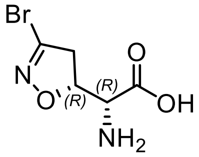

(5*R*,α*R*)-**1d**

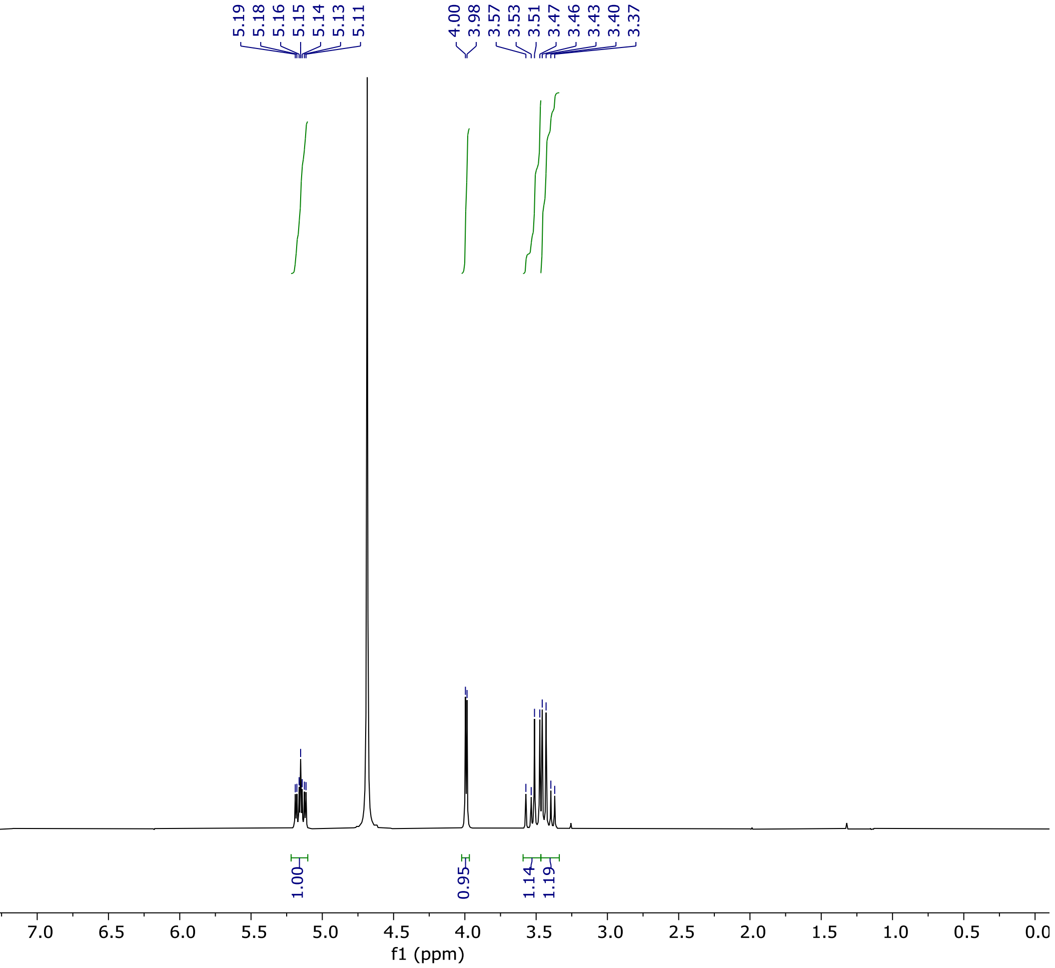

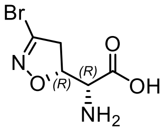

(5*R*,α*R*)-**1d**

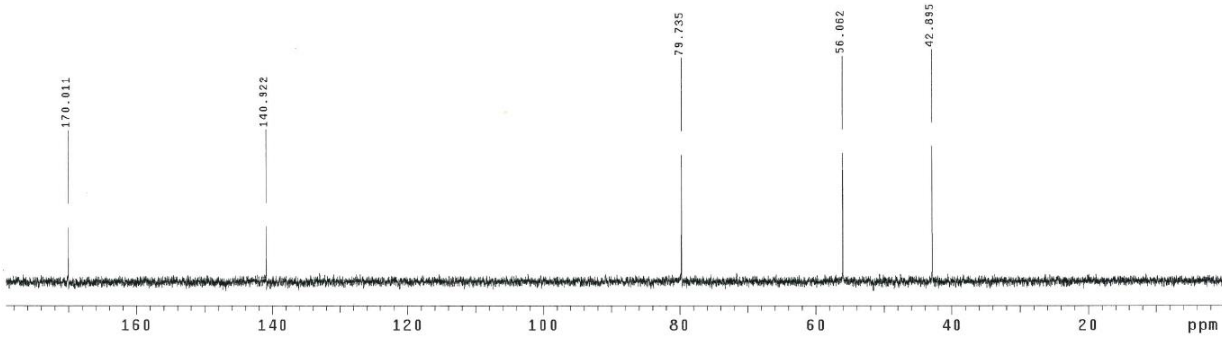

Methyl (*S*)-2-amino-2-((*R*)-3-bromo-4,5-dihydroisoxazol-5-yl)acetate (**2b**):

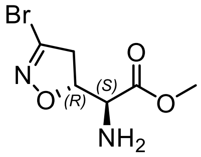

(5*R*,α*S*)-**2b**

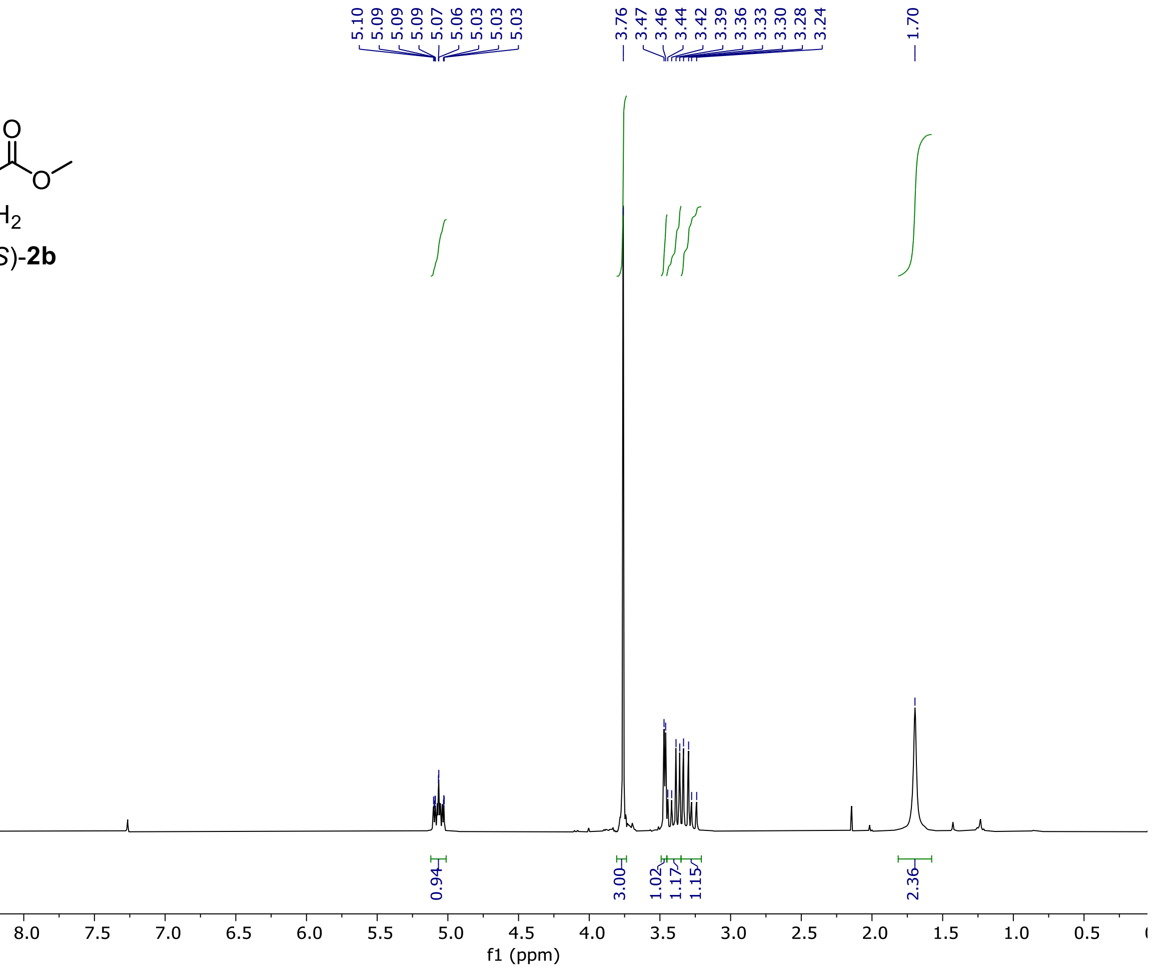

Methyl (*S*)-2-amino-2-((*R*)-3-bromo-4,5-dihydroisoxazol-5-yl)acetate (**2b**):

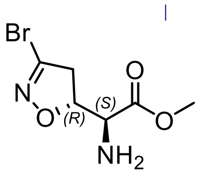

(5*R*,α*S*)-**2b**

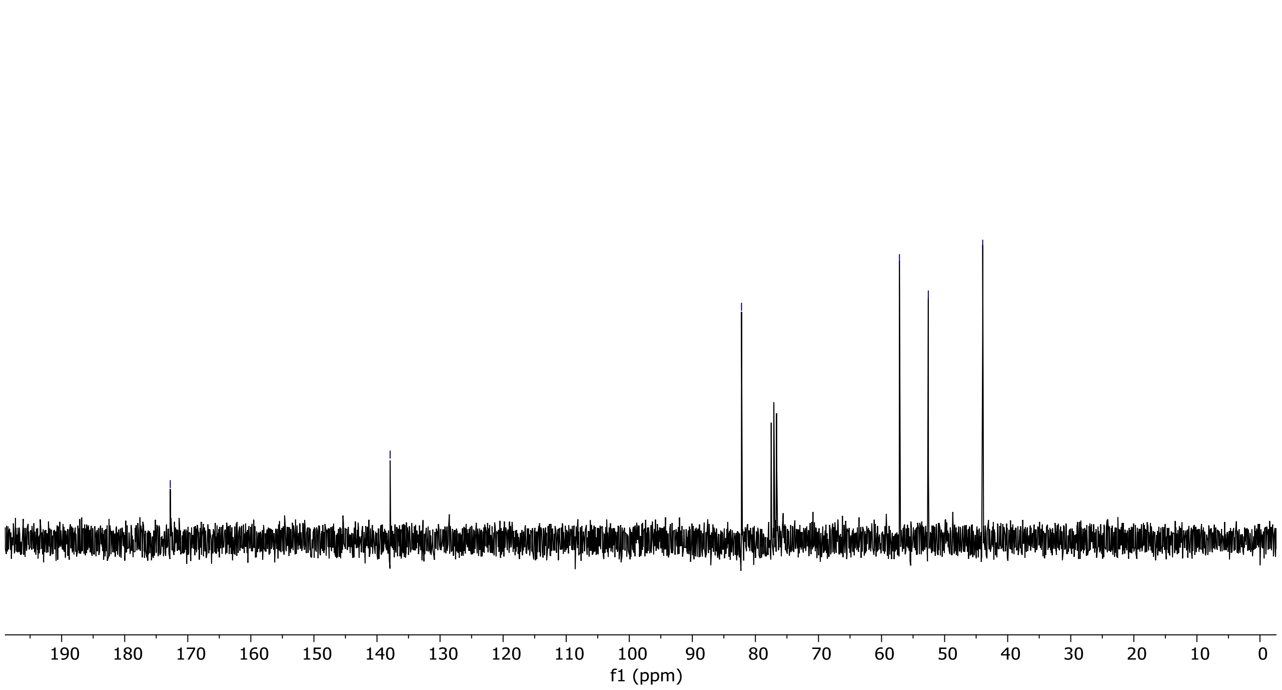

Methyl (*R*)-2-amino-2-((*R*)-3-bromo-4,5-dihydroisoxazol-5-yl)acetate (**2d**):

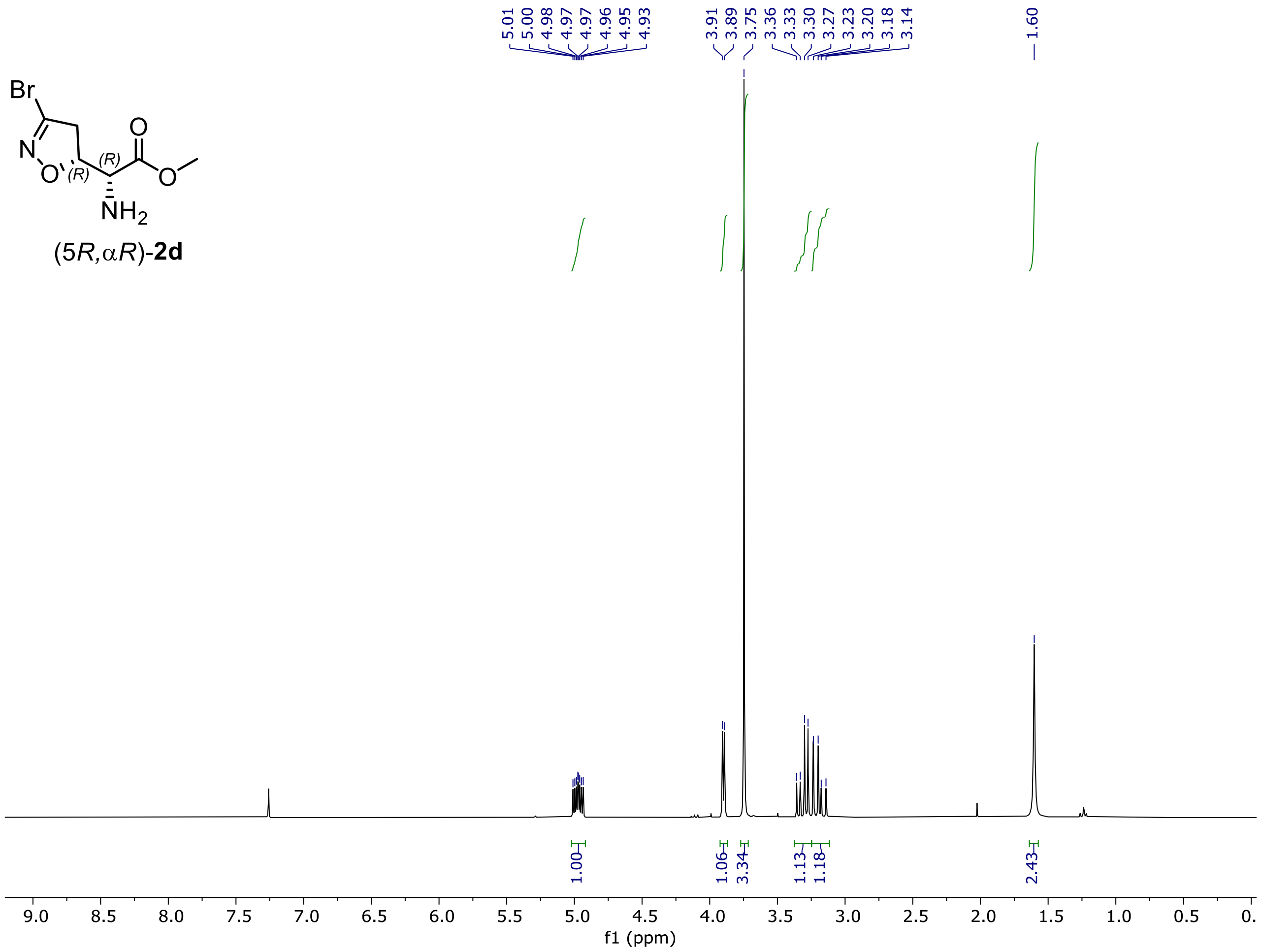

Methyl (*R*)-2-amino-2-((*R*)-3-bromo-4,5-dihydroisoxazol-5-yl)acetate (**2d**):

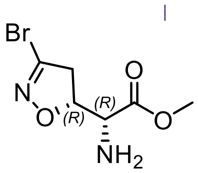

(5*R*,α*R*)-**2d**

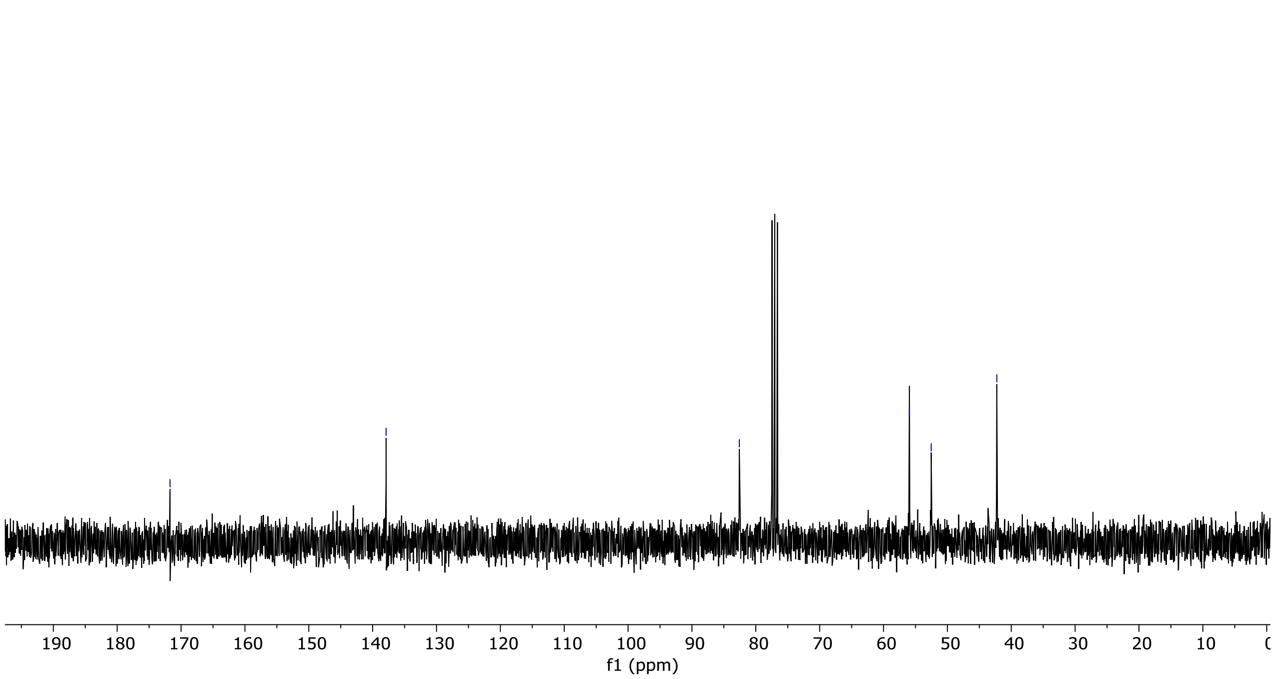

Benzyl (*S*)-2-amino-2-((*R*)-3-bromo-4,5-dihydroisoxazol-5-yl)acetate (**3b**):

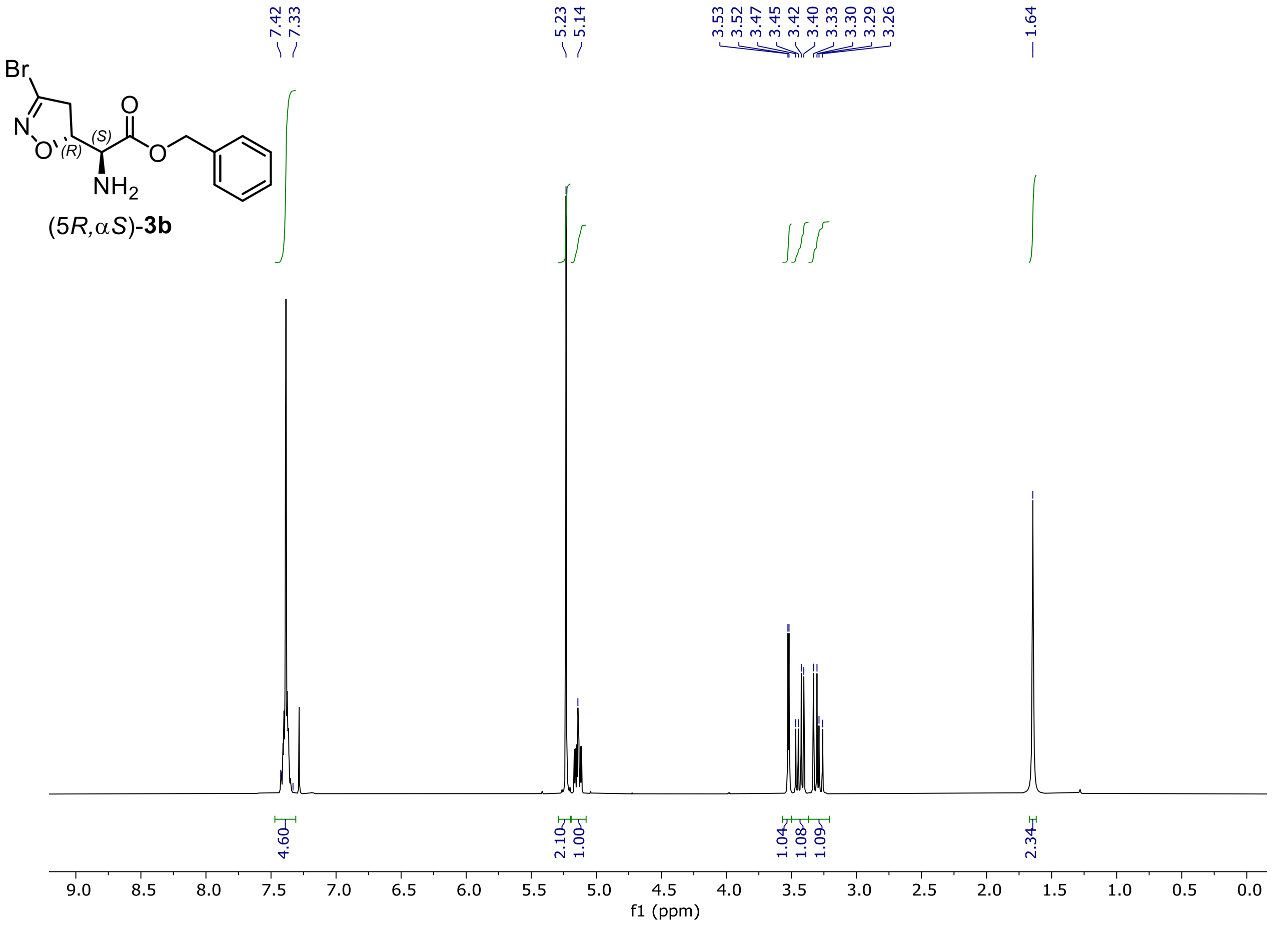

Benzyl (*S*)-2-amino-2-((*R*)-3-bromo-4,5-dihydroisoxazol-5-yl)acetate (**3b**):

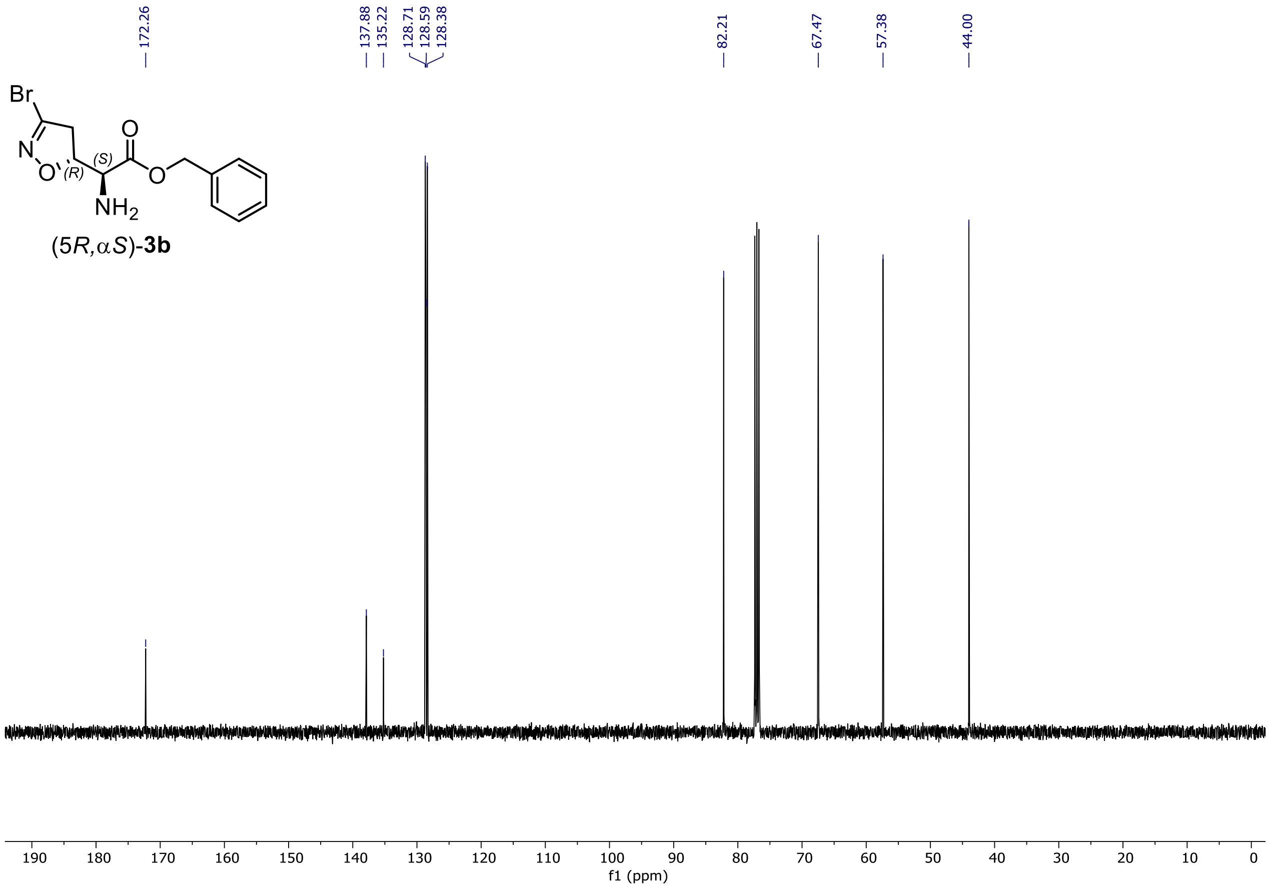

Benzyl (*R*)-2-amino-2-((*R*)-3-bromo-4,5-dihydroisoxazol-5-yl)acetate (**3d**):

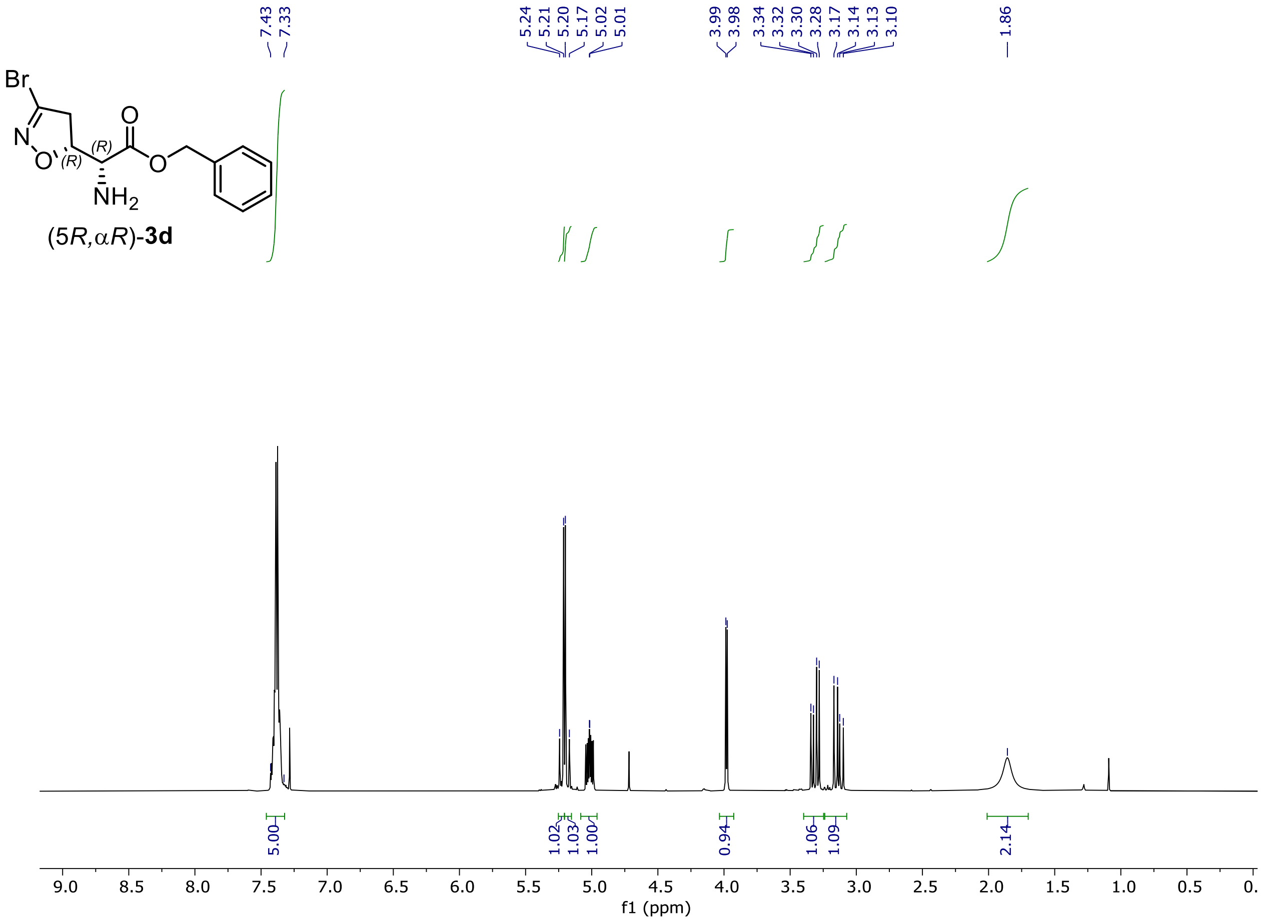

Benzyl (*R*)-2-amino-2-((*R*)-3-bromo-4,5-dihydroisoxazol-5-yl)acetate (**3d**):

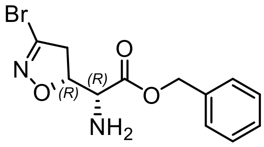

(5*R*,α*R*)-**3d**

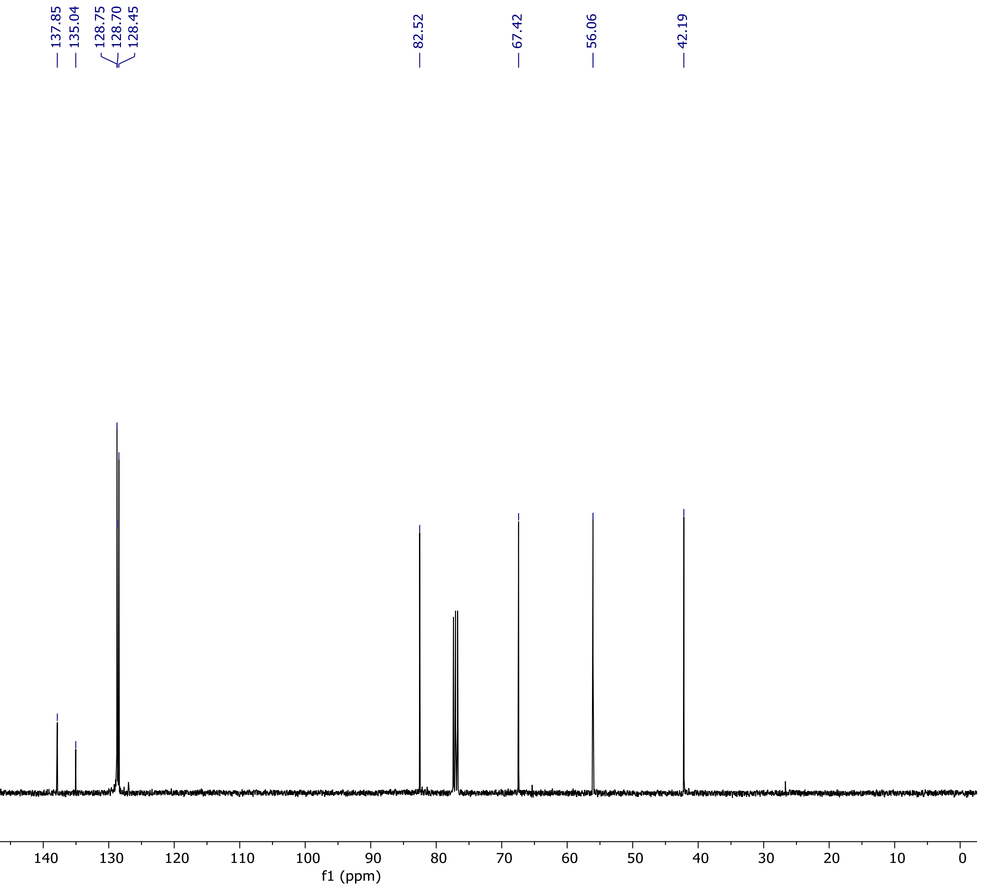

(*S*)-2-amino-*N*-benzyl-2-((*R*)-3-bromo-4,5-dihydroisoxazol-5-yl)acetamide (**4b**):

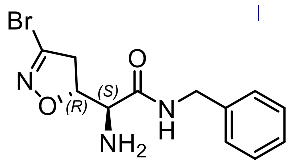

(5*R*,α*S*)-**4b**

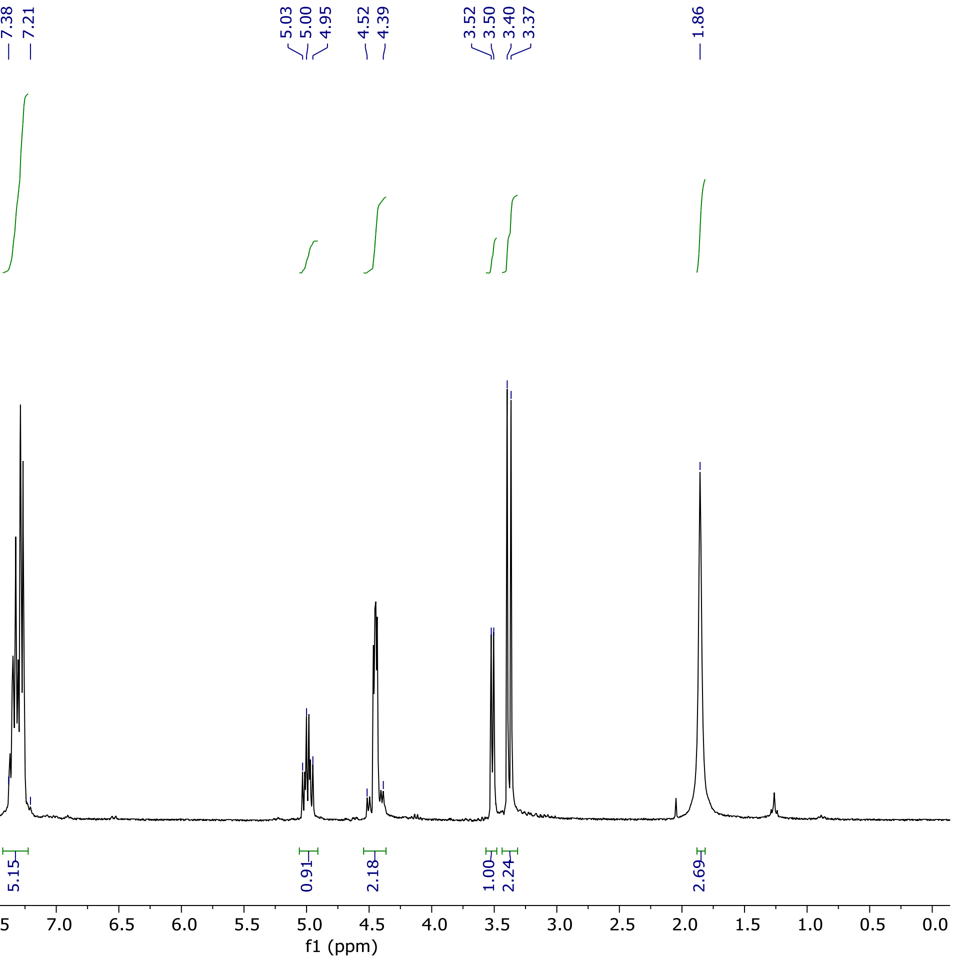

(*S*)-2-amino-*N*-benzyl-2-((*R*)-3-bromo-4,5-dihydroisoxazol-5-yl)acetamide (**4b**):

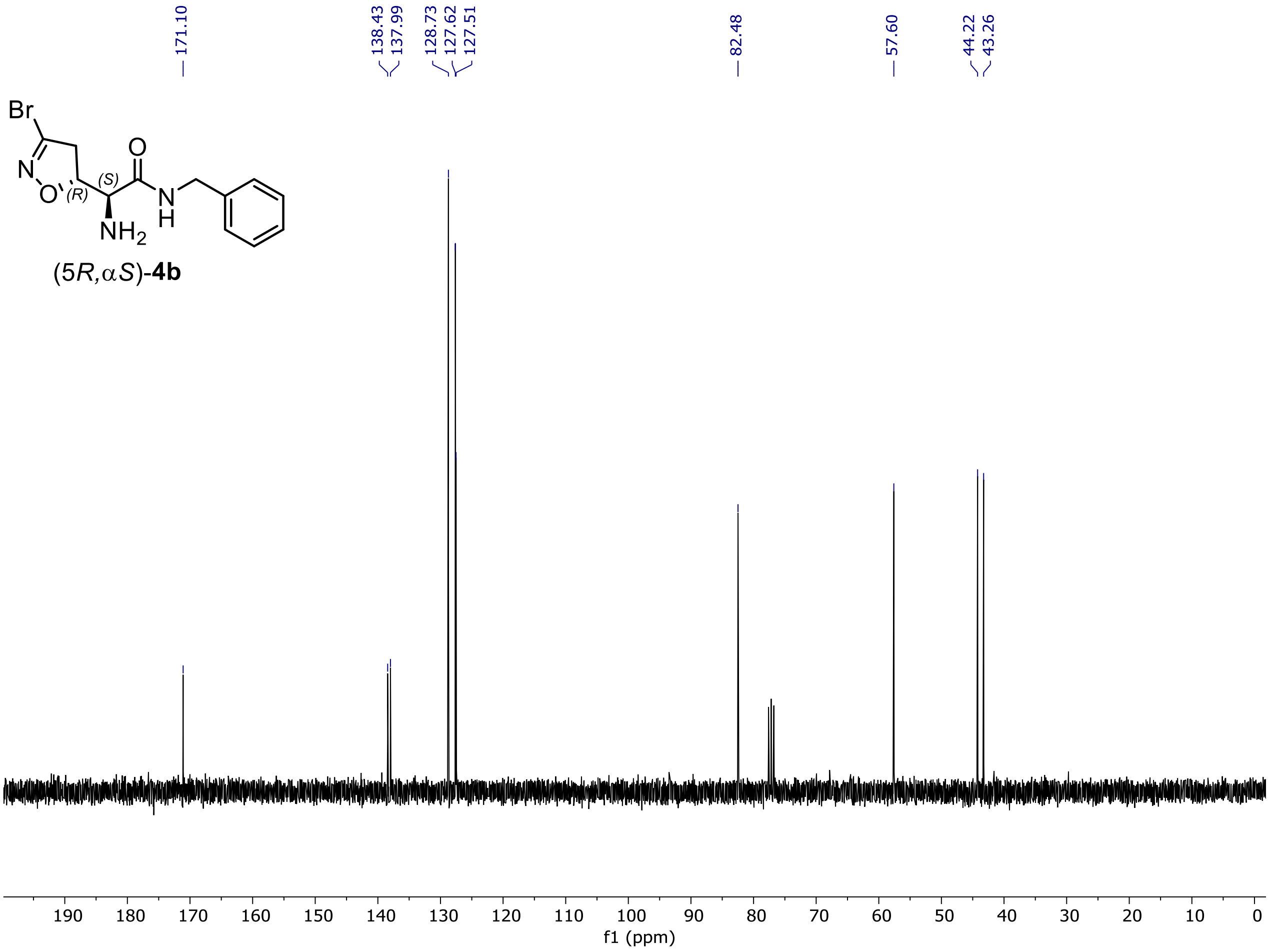

(5*R*,α*S*)-**4b**

(*R*)-2-amino-*N*-benzyl-2-((*R*)-3-bromo-4,5-dihydroisoxazol-5-yl)acetamide (**4d**):

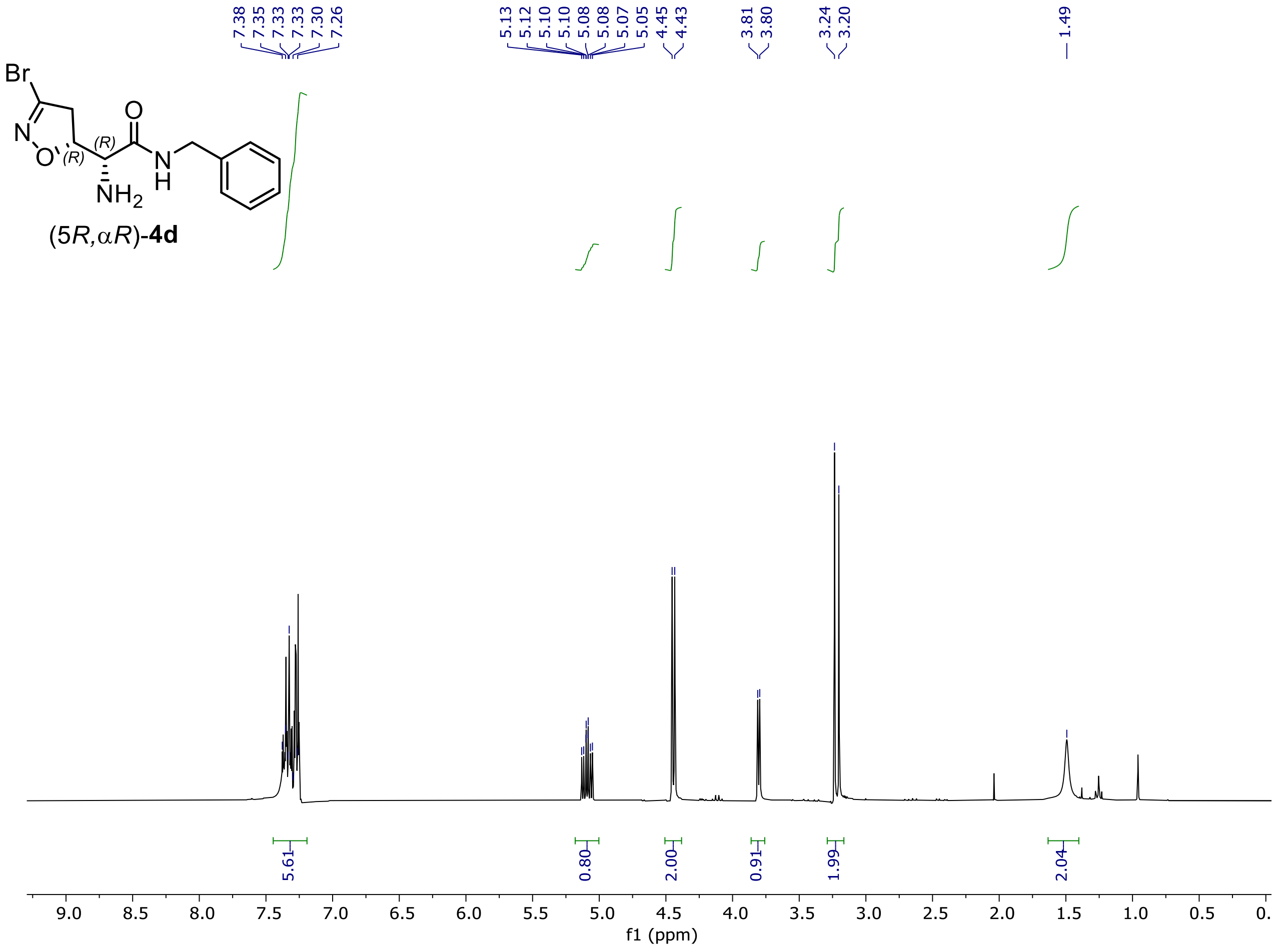

(*R*)-2-amino-*N*-benzyl-2-((*R*)-3-bromo-4,5-dihydroisoxazol-5-yl)acetamide (**4d**):

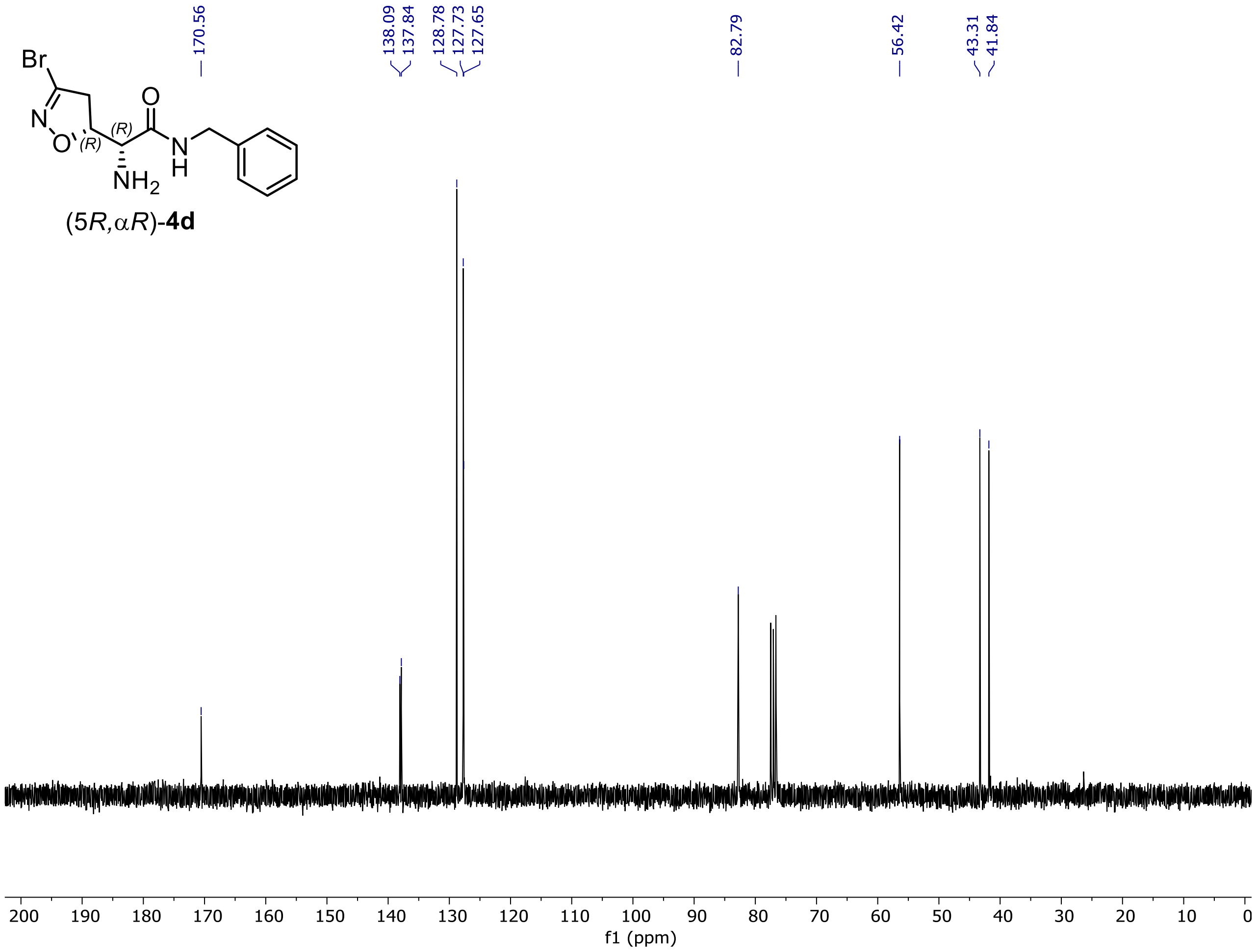

Supplement: Supplementary file 1 [file molecules-28-03172-s001.zip › molecules-2298726-supplementary.pdf]
